# Antihypertensive drug effects on long-term blood pressure: an individual-level data meta-analysis of randomised clinical trials

**DOI:** 10.1136/heartjnl-2021-320171

**Published:** 2022-01-20

**Authors:** Dexter Canoy, Emma Copland, Milad Nazarzadeh, Rema Ramakrishnan, Ana-Catarina Pinho-Gomes, Abdul Salam, Jamie P Dwyer, Farshad Farzadfar, Johan Sundström, Mark Woodward, Barry R Davis, Kazem Rahimi, Blood Pressure Lowering Treatment Trialists Collaboration, Dexter Canoy

**Affiliations:** 1 Deep Medicine, Nuffield Department of Women's and Reproductive Health, University of Oxford, Oxford, UK; 2 NIHR Oxford Biomedical Research Centre, Oxford University Hospitals NHS Foundation Trust, Oxford, Oxfordshire, UK; 3 National Perinatal Epidemiology Unit, Nuffield Department of Population Health, University of Oxford, Oxford, UK; 4 School of Population Health and Environmental Sciences, Faculty of Life Sciences and Medicine, King's College London, London, UK; 5 Department of Community Medicine, Centre for Health Technology and Services Research, University of Porto, Porto, Portugal; 6 The George Institute for Global Health, University of New South Wales, Sydney, New South Wales, Australia; 7 The George Institute for Global Health India, Hyderabad, India; 8 Vanderbilt University Medical Center, Nashville, Tennessee, USA; 9 Endocrinology and Metabolism Institute, Tehran University of Medical Sciences, Tehran, Iran (the Islamic Republic of); 10 Department of Medical Sciences, Uppsala Universitet, Uppsala, Sweden; 11 The George Institute for Global Health UK, Imperial College London, London, UK; 12 Department of Biostatistics, University of Texas School of Public Health, Houston, Texas, USA

**Keywords:** hypertension, meta-analysis, pharmacology, clinical

## Abstract

**Objective:**

Evidence from randomised trials of pharmacological treatments on long-term blood pressure (BP) reduction is limited. We investigated the antihypertensive drug effects on BP over time and across different participant characteristics.

**Methods:**

We conducted an individual patient-level data meta-analysis of 52 large-scale randomised clinical trials in the Blood Pressure Lowering Treatment Trialists’ Collaboration using mixed models to examine treatment effects on BP over 4 years of mean follow-up.

**Results:**

There were 363 684 participants (42% women), with baseline mean age=65 years and mean systolic/diastolic BP=152/87 mm Hg, and among whom 19% were current smokers, 49% had cardiovascular disease, 28% had diabetes and 69% were taking antihypertensive treatment at baseline. Drugs were effective in lowering BP showing maximal effect after 12 months and gradually attenuating towards later years. Based on measures taken ≥12 months postrandomisation, mean systolic/diastolic BP difference (95% CI) between more and less intense BP-lowering treatment was −11.1 (−11.3 to −10.8)/−5.6 (−5.7 to −5.4) mm Hg; between active treatment and placebo was −5.1 (−5.3 to −5.0)/−2.3 (−2.4 to −2.2) mm Hg; and between active and control arms for drug comparison trials was −1.4 (−1.5 to −1.3)/−0.6 (−0.7 to −0.6) mm Hg. BP reductions were observed across different baseline BP values and ages, and by sex, history of cardiovascular disease and diabetes and prior antihypertensive treatment use.

**Conclusion:**

These findings suggest that BP-lowering pharmacotherapy is effective in lowering BP, up to 4 years on average, in people with different characteristics. Appropriate treatment strategies are needed to sustain substantive long-term BP reductions.

## Introduction

Clinical guidelines for managing hypertension have invariably lowered the recommended blood pressure (BP) targets for patients at high risk of cardiovascular disease,^1–7^ informed by evidence from large-scale randomised clinical trials (RCTs) and their meta-analyses showing substantial reductions in cardiovascular risk with more intensive BP-lowering treatment and independently of baseline BP values.^8–14^ For most hypertensive patients, the lowered BP targets inevitably lead to a larger gap between their usual BP and the recommended target value,^15 16^ requiring more intensive pharmacological treatment.

Attributing changes to treatment based on repeated measures of BP of an individual patient can be unreliable since measurements are subject to random fluctuations, regression to the mean, non-pharmacological effects and other sources of variability that can exceed true variability in treatment response.^17–19^ However, it would be useful to have reliable information about the expected effects of drug treatment on BP levels over time from randomised comparisons to help interpret BP readings such as those obtained during clinical encounters. To date, randomised evidence on the effect of antihypertensive drugs on BP has come from efficacy trials with small numbers of highly selected participants and short follow-up durations.^20^ Pooled evidence from RCTs using information from individual participants’ repeated BP measurements currently does not exist, which might explain why there is no guidance on the expected magnitudes of BP reduction with the various proposed treatment strategies and whether these reductions are expected to vary among people with different characteristics.

We addressed this evidence gap by using information from 52 trials involving 363 684 participants with individual-level data on repeated BP measurements over several years^21^ to conduct a meta-analysis to quantify the unconfounded effects of BP-lowering drugs on BP over time and examine these effects across different subgroups.

## Methods

The design of the current phase of the Blood Pressure Lowering Treatment Trialists’ Collaboration (BPLTTC) (www.bplttc.org), including the identification of eligible trials as well as data collection and harmonisation, has been described previously,^21^ with the protocol registered with PROSPERO (CRD42018099283). Briefly, RCTs were eligible for inclusion if there was randomisation of patients between a BP-lowering agent and a placebo arm or inactive control, between various BP-lowering intensities or between various BP-lowering drugs. RCTs should have a follow-up of ≥1000 person-years in each randomly allocated arm to minimise the risk of small-study effects.^22^ Trials without a drug comparison arm or without description of randomisation process were not eligible, nor were those conducted exclusively in patients with heart failure or investigating short-term interventions (eg, in acute care settings). The protocol for the current analyses was reviewed and approved by the BPLTTC Steering Committee prior to data analysis.

To analyse the data, we assigned each participant according to their random allocation in the individual trials, either to the active (or treatment) arm or to the control group separately for each trial design, as described in online supplemental methods and table S1, and compared BP levels between these comparison groups. Our study outcomes were mean systolic and diastolic BP differences between comparison arms.

10.1136/heartjnl-2021-320171.supp1 Supplementary data

### Statistical analysis

We used a one-stage approach to conduct the meta-analysis of repeated BP measurements over time and applied linear mixed models to estimate the effect of treatment on BP between comparison arms. We developed and compared models that accounted for clustering by trial and potential variability due to baseline BP and other trial-level and participant-level sources of heterogeneity, and determined the best fitting model for our data (online supplemental methods 1). Our primary model was based on fixed treatment effect and fixed time effect but allowing for random intercepts at trial and participant levels and a random slope for follow-up duration at participant level.

We estimated BP values and their difference between comparison groups during the course of follow-up, separately by trial design. As the early phase of the treatment may involve adjustments to optimise treatment regimens such as dosage titration,^23^ BP difference between treatment arms may not be maximally achieved until after this period. We therefore also analysed results with and without inclusion of BP measurements taken <12 months after randomisation. We used published aggregate information on achieved BP for each comparison arm to estimate individual-level follow-up values where follow-up BP measurements were not accessible (online supplemental methods 1). We then investigated treatment effects stratified by participants’ baseline BP, age, sex, body mass index, history of cardiovascular disease and diabetes and prior use of antihypertensive medication, and assessed any heterogeneity by comparing models with and without an interaction term for the characteristic of interest and treatment allocation. Models were adjusted for baseline BP, age at recruitment and sex (except when used as stratification factors). We also ran sensitivity analyses that excluded data from each trial and examined results by study period (based on the year the trial has ended).

We used likelihood ratio test (for nested models) and the Akaike information criterion (for non-nested models) to compare models and reported estimates with their 95% CI and p values that were tested at 5% significance level (two tailed). We used R (V.3.4.4)^24^ to analyse the data.

### Patient and public involvement

There was no patient or public engagement in the design or conduct of this study.

## Results

### Characteristics of trials and participants in the BPLTTC

The 52 included trials comprised of nine BP-lowering intensity trials, 21 placebo-controlled trials and 29 drug class comparison trials (table 1), mostly conducted between 1990 and 2009 (eight trials conducted after 2009). Seven trials included both comparisons between drug classes as well as either intensity of BP-lowering or between active treatment and placebo. On average, the trials had 4 years of follow-up and eight BP measurements collected after baseline.

**Table 1:** Characteristics of trials and participants

Characteristics	Blood pressure (BP) difference trials			Drug class comparison trials	All trials
	BP-lowering intensity	Placebo controlled	All BP difference trials		
Trials					
No. of trials	9	21	30	29	52*
No. of trials by year of end of study					
Before 1990	1	2	3	0	3
1990–1999	2	7	9	7	14
2000–2009	2	10	12	19	27
After 2009	4	2	6	3	8
Mean (SD) trial duration (years)	4 (2)	4 (2)	4 (2)	4 (2)	4 (2)
Mean (median) no. of follow-up BP measures	14 (13)	7 (6)	8 (8)	8 (7)	8 (8)
Participants					
No. of participants (% women)	35 934 (45)	112 934 (35)	148 873 (38)	224 038 (44)	363 684 (42)
% (n/N) Caucasian/European ethnicity	46 (15 863/34 823)	68 (58 851/86 908)	61 (74 714/121 731)	64 (118 128/185 351)	63 (188 948/297 852)
% (N) current smoker at baseline^b^	22 (8238/35 908)	16 (17 702/111 190)	18 (25 940/147 098)	20 (44 173/220 708)	19 (68 360/359 719)
Mean (SD) baseline SBP/DBP	151 (21)/88 (15)	146 (21)/83 (11)	147 (21)/84 (12)	156 (21)/90 (12)	152 (21)/87 (12)
% (N) participants by baseline SBP (mm Hg)					
<120/<70	4 (2870)/11 (3806)	8 (9176)/9 (10 037)	7 (10 650)/9 (13 843)	3 (7027)/5 (10 410)	5 (17 128)/7 (23 803)
120–129/70–79	9 (6228) / 17 (6075)	13 (15 063)/24 (26 927)	13 (18 448)/22 (33 002)	6 (12 969)/14 (31 330)	9 (30 720)/17 (63 091)
130–139/80–89	18 (11,289) / 24 (8593)	17 (19 674)/39 (43 738)	18 (26 077)/34 (50 623)	11 (23 906)/28 (62 292)	14 (48 820)/30 (109 589)
140–149/90–99	19 (11 393)/29 (14 890)	18 (20 590)/21 (24 043)	19 (27 386)/23 (34 324)	18 (41 220)/30 (67 403)	19 (66 928)/27 (98 994)
150–159/100–109	17 (10 050)/14 (6342)	14 (16 246)/7 (7355)	14 (21 107)/8 (12 264)	19 (42 509)/18 (39 839)	17 (61 495)/14 (51 014)
≥160/≥110	33 (19 050)/6 (2696)	28 (32 114)/1 (750)	30 (43 396)/2 (2994)	43 (95 833)/5 (12 188)	38 (136 226)/4 (14 810)
Mean (SD) age (years) at baseline	61 (12)	65 (10)	64 (11)	65 (9)	65 (10)
% (N) of participants by age at baseline					
<50 years	16 (7146)	5 (5596)	8 (11 256)	4 (9542)	5 (19 122)
50–59 years	34 (18 465)	22 (24 668)	25 (36 978)	24 (52 819)	24 (86 699)
60–69 years	27 (18 005)	39 (44 374)	36 (54 016)	39 (87 144)	38 (137 849)
70–79 years	18 (13 313)	26 (28 921)	24 (35 264)	28 (63 119)	27 (97 290)
≥80 years	6 (3999)	8 (9342)	8 (11 321)	5 (11 369)	6 (22 638)
% (N) with condition at baseline†					
Cardiovascular disease	16 (5617/35 934)	66 (72 209/110 020)	54 (78 738/145 945)	45 (98 944/221 993)	49 (175 519/359 357)
Coronary heart disease	11 (4120/35 934)	41 (45 591/110 008)	34 (49 711/145 942)	38 (67 766/177 363)	37 (115 562/316 125)
Stroke	3 (966/34,840)	34 (32 650/95 800)	28 (36 521/130 643)	11 (17 830/168 003)	18 (51 320/292 559)
Diabetes	24 (8540/35 934)	36 (36 179/100 697)	33 (44 719/136 631)	26 (58 404/223 654)	28 (99 375/351 357)
Chronic kidney disease	33 (4854/14 799)	9 (2845/25 789)	19 (7699/40 588)	17 (18 917/108 612)	17 (24 289/145 895)
% (N) previously on BP-lowering medication†	34 (12 141/35 934)	71 (73 833/103 766)	65 (73 237/126 502)	77 (119 454/155 069)	69 (202 428/293281)
Mean (SD) body mass index† (kg/m^2^)	29 (6)	28 (5)	28 (5)	28 (5)	28 (5)

*Some trials provided data to more than one trial design.

†Data limited to those with relevant information and N refers to the denominator for number of participants with information on the relevant variable.

SBP, systolic blood pressure; DBP, diastolic blood pressure.

The trials included 363 684 randomised participants (42% women) with a mean age of 65 years at baseline, including 6% aged ≥80 years. The mean baseline systolic/diastolic BP was 152/87 mm Hg (73% with ≥140 mm Hg systolic and 46% with ≥90 mm Hg diastolic BP) across all trials, with higher values for drug class comparison trials than the other designs (table 1). At baseline, 49% of all participants had had a history of cardiovascular disease and a third a history of diabetes. Baseline BP was higher for older persons, in women and among those with lower body mass index, without cardiovascular disease or diabetes and no prior use of antihypertensive medications, as compared with their counterparts (online supplemental table S2). Further details about study methods, design and risk of bias assessment for each trial are shown in online supplemental table S3 to S7).

### Temporal BP patterns by treatment allocation

**Figure 1:**
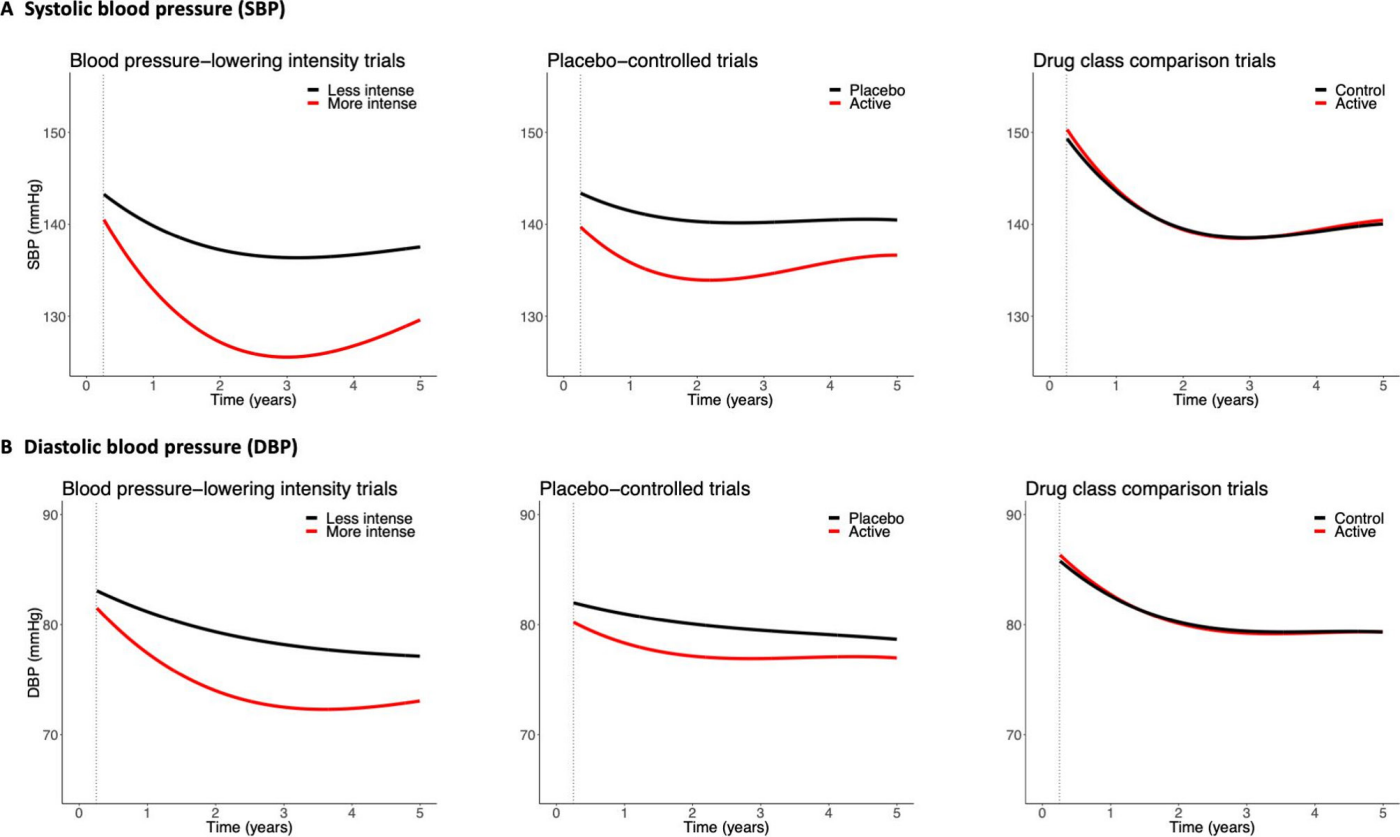
Blood pressure (BP) trajectories according to different trial designs. Results are in red for active group and black for control group, from 3 months to 5 years of follow-up. Estimates were based on separate models for treatment and control groups, with random intercepts at individual and trial levels, a random slope for time at the individual level (see Method for details) and adjusted for baseline BP, age and sex. Baseline systolic/diastolic BP for active and control groups were: BP-lowering trials=151/88 mm Hg; placebo-controlled trials=146/83 mm Hg and drug class comparison trials=156/90 mm Hg. Estimated BP at specific time points are shown in online supplemental table S8). Results for all BP difference trials are shown in online supplemental figure S2.

The temporal patterns of BP are shown in figure 1 (additional information in online supplemental table S8). Across all trial designs, BP fell during the first few months of follow-up in both study arms. For BP-lowering intensity and placebo-controlled trials, there was divergence in BP in the early follow-up period that increased over time—BP levels in the active arm were lowest at around 2 years after baseline. For drug class comparison trials, BP levels in both comparison arms remained similar during follow-up. The mean BP achieved in the active arm of BP-lowering intensity trials was substantially lower than those achieved in the active arms of the other trial designs. Results for all BP difference trials are shown in online supplemental figure S2.

**Figure 2:**
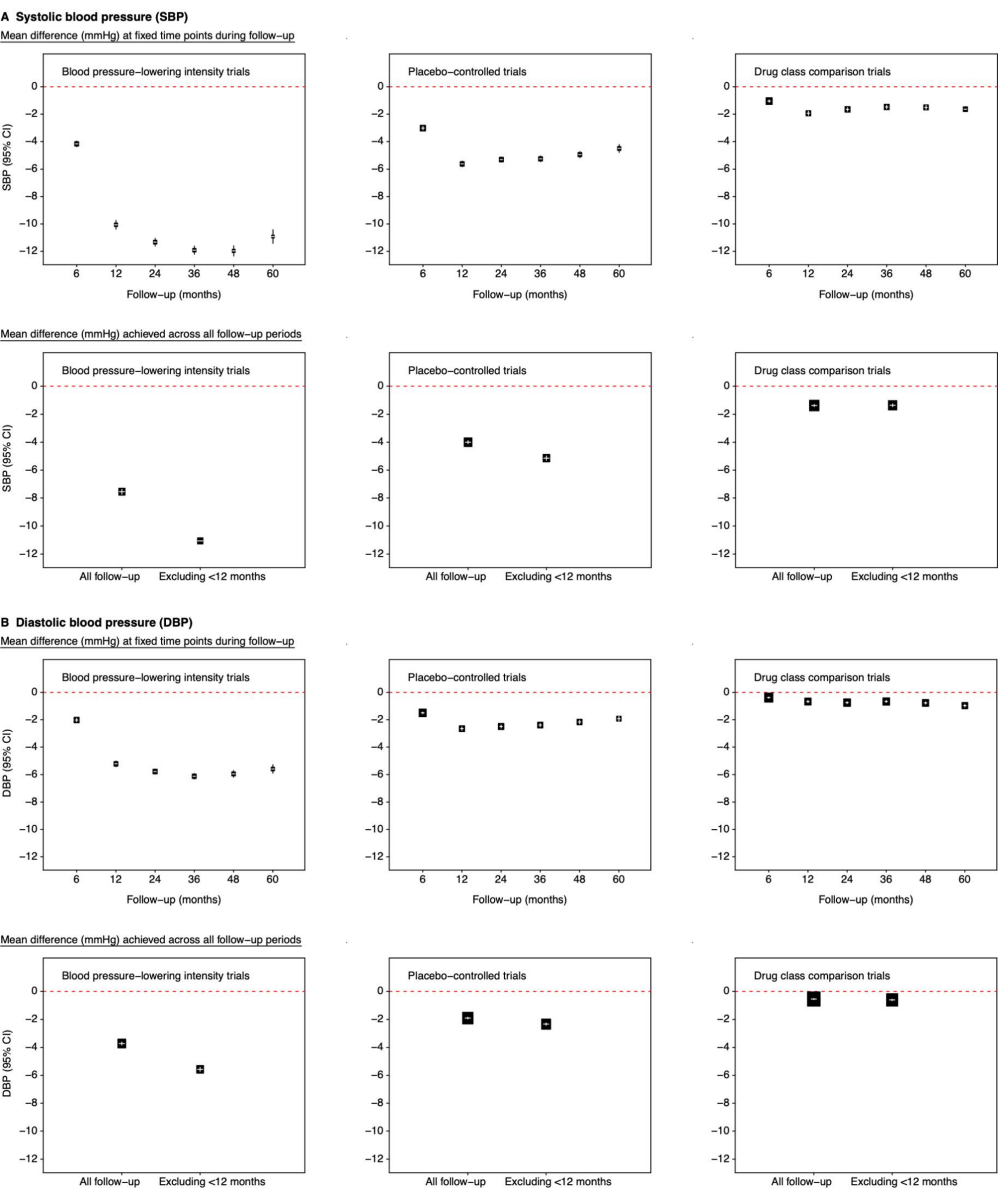
Effects of blood pressure (BP)-lowering treatment on mean BP at fixed follow-up time points and across all follow-up period. (A) Systolic BP; (B) Diastolic BP. For mean difference at fixed follow-up time periods, estimates were based on separate models for each time period with a fixed treatment effect and random intercept for individuals. For mean difference achieved across all time period (showing results based on all follow-up BP measures and measures obtained from 12 months until end of follow-up), estimates were based on fixed treatment effect and random intercepts at individual and trial levels, a random slope for time at the individual level. All mean difference values were adjusted for baseline BP, age and sex. The area of the square is inversely proportional to the variance of the estimated difference. Negative values indicate lower BP in the active than in the control group. Additional information provided in online supplemental table S9 and S10), and results for all BP difference trials are in online supplemental figure S3.

### Achieved net BP reduction by follow-up period

Figure 2 (additional details in online supplemental table S9) illustrates the varying estimates of the difference in BP between comparison groups at specific follow-up times. Consistent with the patterns of absolute BP levels, the estimated difference in BP achieved between the active and control groups tended to be lower in earlier than in later follow-up periods. For BP-lowering intensity trials, the difference in mean reductions in systolic and diastolic BP within 6 months from baseline were −4.2 (95% CI −4.4 to −4.0) mm Hg and −2.0 (95% CI −2.2 to −1.9) mm Hg, respectively, and over −10 mm Hg and −5 mm Hg reductions, respectively, based on measures taken at later follow-up periods. Similar patterns were seen for placebo-controlled trials (and BP difference trials, details shown in online supplemental figure S3), although this group achieved smaller magnitudes in mean BP reduction across all follow-up periods. Mean reductions were least for drug class comparison trials.

**Figure 3:**
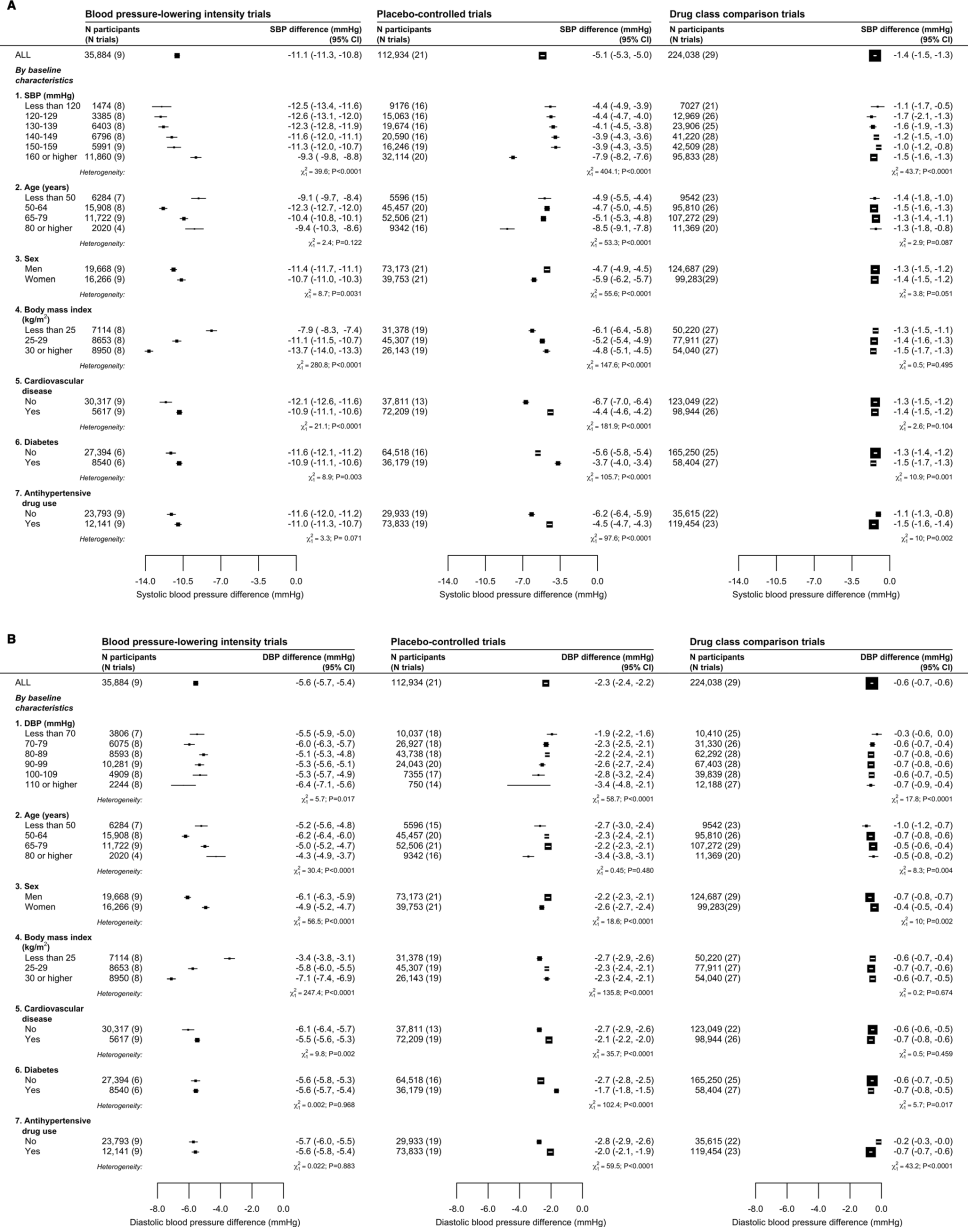
Effects of blood pressure (BP)-lowering treatment on mean BP, by baseline characteristics. (A) mean systolic BP difference; (B) mean diastolic BP difference. Estimates based on fixed treatment effect and random intercepts at individual and trial levels, a random slope for time at the individual level (see Method for details) and adjusted for baseline BP), age and sex except when these variables are used as stratification factors. The area of the square is inversely proportional to the variance of the estimated difference. Negative values indicate lower BP in the active than in the control group. Results for all BP difference trials are in online supplemental figure S4. To provide context of background BP levels, baseline BP by these subgroups are shown in online supplemental table S2.

### Estimating overall achieved BP reduction between comparison groups

The time-related BP differences between comparison groups affected the overall achieved reduction in BP. Estimates based on BP measures obtained across all follow-up period were relatively smaller in magnitude than when the treatment phase of <12 months was excluded (figure 2, further details in online supplemental figure S3 and table S10). For example, for BP-lowering intensity and placebo-controlled trials, the overall mean systolic/diastolic BP reductions across the whole follow-up time were −7.6 (95% CI −7.7 to −7.4)/−3.7 (95% CI −3.8 to −3.6) mm Hg and −4.0 (95% CI −4.1 to −3.9)/−1.9 (95% CI −2.0 to −1.8) mm Hg, respectively; when using measurements taken ≥12 months from baseline, the achieved reductions were −11.1 (95% CI −11.3 to −10.8)/−5.6 (95% CI −5.7 to −5.4) mm Hg and −5.1 (95% CI −5.3 to −5.0)/−2.3 (95% CI −2.4 to −2.2) mm Hg, respectively.

### Effects of treatment on BP reduction across different subgroups

Focusing on BP differences from ≥12 months from baseline, figure 3 and online supplemental figure S4 show treatment effects by different baseline characteristics. There were some variations in the magnitudes of BP reductions, notably by body mass index categories in BP-lowering intensity trials. Some trials disproportionately contributed more data in some subgroups so the results reflected features of these trial characteristics and design. For example, the Systolic Blood Pressure Intervention Trial (SPRINT) and the Action to Control Cardovascular Risk in Diabetes (ACCORD) trial, which achieved substantive BP reductions, included participants with higher mean baseline body mass index whereas Hypertension Objective Treatment Based on Measurement by Electrical Devices of Blood Pressure (HOMED-BP) and Valsartan in Elderly Isolated Systolic Hypertensioon Study (VALISH) trials, which achieved modest BP reductions, included those with lower mean baseline body mass index (online supplemental table S6). While there were variations in treatment effects across different subgroups, BP reductions were evident across these subgroups, even among those with baseline systolic BP <120 mm Hg and diastolic BP <70 mm Hg. For drug class comparison trials, BP differences overall and across subgroups were consistently small (figure 3).

### Sensitivity analyses

BP differences achieved by each trial are reported in online supplemental figure S5. Results after excluding one trial at a time largely showed similar results as the overall estimates within each trial type (online supplemental figure S6). There were little differences in the achieved BP reductions by study period except in placebo-controlled trials that achieved greater reductions in trials that ended before 2000 than in newer trials (online supplemental table S11), due to some older trials that had far higher starting mean baseline BP values than the newer trials but with comparable treatment goals (online supplemental table S6 and figure S5). Finally, online supplemental table S12 shows how the models we used fitted the data better and gave more conservative estimates than models that did not take into account time-related variations and other individual-level factors in treatment effects.

## Discussion

The analysis of individual-level data of 363 684 randomised participants of 52 large-scale RCTs, the largest of such meta-analysis to date, provides evidence to the overall and stratified effects of antihypertensive treatment on relatively long-term BP reduction. The magnitude of BP reduction varied by time after randomisation and the intended trial intervention. The predicted maximum effect of intervention became apparent about a year from randomisation, with some gradual attenuation several years later during follow-up. The net achieved BP reduction varied by trial design, with BP-lowering intensity trials achieving the largest mean reduction of over 11 mm Hg systolic BP after the first year of treatment. The effects were evident across patient subgroups, as defined by their baseline BP, age, sex, body size, history of cardiovascular disease or diabetes and prior use of antihypertensive treatment.

Randomised evidence on the expected effect of antihypertensive drugs on BP has been largely based on published information from efficacy trials. In a meta-analysis of 354 trials (N≈56 000),^20^ half-standard dosages of one, two or three antihypertensive drugs led to systolic BP reductions of 6.7 mm Hg, 13.3 mm Hg and 19.9 mm Hg, respectively, from a pretreatment systolic/diastolic BP of 150/90 mm Hg. Our study is not directly comparable with this work, but it is notable that, in our study, the mean BP reductions were less pronounced than their estimates and that the full effects became evident only after a several months after initiating therapy. This discrepancy could be due to a number of reasons. Their meta-analysis included trials with relatively short follow-up duration (around 2–16 weeks), with some trials having potentially restricted their analysis to fully adherent participants. In contrast, we included large-scale trials with 4-year mean follow-up and performed analysis as per intention to treat. By design, many trials included in our study focused on achieving a target BP level or reduction, so the maximal physiologically feasible effect on BP reduction may not have been achieved. A substantial proportion of participants were on antihypertensive drugs at baseline, which could have further underestimated the magnitude of achieved BP reduction, although it should not have an impact on the net between-group BP reductions.

Current clinical practice guidelines typically recommend a gradual intensification of antihypertensive treatment over several weeks and monitoring of its response for the treated individual.^1–6^ However, there is no clear guidance as to the expected change in BP on initiating treatment. To gauge treatment response without a counterfactual or ‘standard’ to compare against is difficult because of the multitude of other causes of BP change.^17 18^ Estimates of longitudinal BP changes in our study may help mitigate exaggerated attributions of change in BP to treatment, while providing reassurance about achievable reductions in various groups of ‘at-risk’ individuals. Clinical guidelines also typically define specific BP targets that should be achieved for hypertension to be considered as ‘controlled’, although target levels set by different national guidelines vary.^1–6^ While setting a target has practical advantages, it assumes that it is achievable on full implementation of the guidelines. However, population BP follows a distribution, with mean systolic BP≈130 mm Hg in Western populations and over 60% by age ≥60 years have values >140 mm Hg.^25 26^ Among the most intensive treatment strategies in the clinical outcome trials were able to achieve an average of 10–15 mm Hg systolic BP reductions within a few months to several years (eg, SPRINT achieved 15 mm Hg systolic BP reduction (online supplemental figure S5). With current evidence-based treatment recommendations, achieving a controlled BP for people with very high BP (eg, >150 mm Hg systolic), would be difficult to attain with the trialled regimens of pharmacologic treatment.^27^ We do not imply that physiologically larger BP reductions are unachievable but rather intend to flag the limited evidence on pharmacological BP reductions of over 20 mm Hg in the long term. The achieved BP reduction estimated in our pooled analysis has implications not just for patients but also for healthcare providers whose performance will be assessed based on their patients achieving ‘controlled’ BP. Alternative monitoring strategies, such as the number of prescribed antihypertensives^28^ for an individual as opposed to using a single BP target for all, are needed. Some translational implications of this study are described further in the online supplemental file 1– clinical perspectives.

Recommendations for BP management in specific patient groups also remains controversial. The US guidelines suggest similar recommendations for people with and without pre-existing cardiovascular disease,^1^ but the UK guidelines use a higher BP threshold for people without cardiovascular disease due to lack of any direct evidence of efficacy in this patient group.^29^ Although there were some variations in the treatment effects in our stratified analyses, which were likely an artefact of trial design, BP reductions were evident across a wide range of baseline BP and other personal characteristics. Unsurprisingly, there was little difference in magnitude of BP reduction between comparison arms of drug comparison trials (overall and across subgroups). The BP values substantially fell from baseline in both arms, which is likely due to regression to the mean given the high baseline BP of patients in these trials.^17^ The extent to which the estimated BP reductions will have an impact on existing evidence base, which have either been based on published information on average BP differences for each trial^8^ or have not adjusted for achieved BP differences between trials,^30^ requires further investigation.

A number of limitations need to be considered when interpreting our findings. Investigators or data custodians of some eligible trials could not be contacted (particularly for older trials) or were unwilling to take part in the collaboration. Nevertheless, the trials included in our collaboration generally have low risk of bias. Short-term effects of BP-lowering agents are well established,^20^ and our findings extend these effects over a relatively longer period of follow-up of 4 years on average (few trials had over 5 years of follow-up). We could not compare drug classes based on standardised dosages, as most treatment interventions allowed titration or addition of other drug classes to achieve specific treatment goals (online supplemental table S3). Investigators were allowed to add non-study antihypertensive treatment in some trials, which could have led to the dilution of treatment effects between trial arms or subgroups. Adherence to treatment had fallen towards the end of follow-up in most trials (online supplemental table S5), which could partly explain why treatment effects were lower in these latter follow-up periods. We did not have full access to individual-level information about use of non-study drugs nor on adherence to treatment to be able to quantify their effects. Yet an important strength of our study is that it permitted comparison across subgroups while maintaining the advantage of the random allocation to treatment groups.

Our study highlights the role of pharmacological agents in effectively reducing BP over several years across individuals with a wide range of characteristics, although the achieved between-group reductions, even with the intensive BP-lowering regimens, were relatively modest. Given that large-scale trials have shown the effects of pharmacological BP reduction on improving clinical outcomes, the modest BP reductions estimated in our study should still be clinically meaningful.^14^ Indeed, the estimates of long-term BP reduction in this study could inform treatment strategies and help in setting realistic treatment goals in the pharmacologic management of raised BP.

**Key messages**

**What is already known on this subject?**

- Randomised evidence of the effects of antihypertensive drugs on achievable blood pressure reduction has been based on trials with small sample sizes and short treatment periods of several weeks; pooled analysis of randomised evidence to provide reliable estimates of achievable long-term blood pressure reduction from pharmacological treatment is lacking.

**What might this study add?**

- This large-scale individual participant-level data meta-analysis has shown that the patterns of blood pressure reduction differed over time, with the maximum effect seen in intensive treatment strategies that achieved 11 mm Hg systolic blood pressure reduction on average after the first year of treatment. Beneficial effects were demonstrable over wide ranges of baseline blood pressure, ages and body sizes, in women and men, by history of cardiovascular disease or diabetes and by prior use of antihypertensive treatment.

**How might this impact on clinical practice?**

- The efficacy of antihypertensive drugs was demonstrable across different population subgroups, although the achieved blood pressure reductions, even with trialled intensive regimens, were relatively modest. These findings could guide setting realistic treatment goals in the pharmacological management of raised blood pressure.

## Data Availability

Data may be obtained from a third party and are not publicly available. The governance of the BPLTTC have been reported previously. The BPLTTC is governed by the University of Oxford’s policies on research integrity and codes of practice and follows the university’s policy on the management of research data and records. Scientific activities using the BPLTTC data resource are overseen by the Steering Committee of the collaboration. All data shared with the BPLTTC are considered confidential and will not be provided to any third party. Requests for data should be made directly to the data custodians of individual trials.
