# Supplementary material for: Antihypertensive drug effects on long-term blood pressure: an individual-level data meta-analysis of randomised clinical trials
Source: Heart. 2022 Jan 20;108(16):1281–9. doi: 10.1136/heartjnl-2021-320171 (PMC9340038; doi:10.1136/heartjnl-2021-320171)
Supplement: Supplementary data [file heartjnl-2021-320171supp001.pdf]

Supplementary Materials

Antihypertensive drug effects on long-term blood pressure: an individual-level data meta-analysis of randomised clinical trials

Table of contents

The Blood Pressure Lowering Treatment Trialists' Collaboration ……………………………….... **p 2**
Supplementary Methods …………………………………………………………………….…... **p 4**

Figure S1. Schematic diagram for model development and comparison ……………………………... **p 7**
Figure S2. Long-term mean blood pressure in the BPLTTC for all blood pressure difference trials (blood pressure-lowering intensity and placebo-controlled trials combined) ……………………………… **p 8**
Figure S3. Effects of blood pressure-lowering treatment on mean blood pressure at fixed follow-up time points and across all follow-up period for all blood pressure difference trials (blood pressure-lowering intensity and placebo-controlled trials combined) …………………………………………………………… **p 9**
Figure S4. Effects of blood pressure-lowering treatment on long-term mean blood pressure, by baseline characteristics in all blood pressure difference trials (blood pressure-lowering intensity and placebo-controlled trials combined) …………………………………………………………………………….. **p 10**
Figure S5. Effects of blood pressure-lowering treatment on mean long-term blood pressure for each trial …………………………………………………………………………………………………… **p 12**
Figure S6. Effects of blood pressure-lowering treatment on long-term mean blood pressure after excluding one trial at a time ……………………………………………………………… …………………… **p 14**

Table S1. List of trials and interventions assigned to the active and control arms in the analyses …… **p 15**
Table S2. Mean baseline blood pressure according to baseline characteristics and trial designs .... **p 17**
Table S3. Characteristics of trials included in the BPLTTC ……………………………………………….. **p 19**
Table S4. Risk of bias assessment of each trial ……………………………..……………………….. **p 28**
Table S5. Indicators of numbers of blood pressure-lowering drug classes given and adherence to assigned treatment, separately by trial design ……………………………………………………………… **p 29**
Table S6. Characteristics of participants at baseline for each trial ……………………………………….. **p 35**
Table S7. Blood pressure measurement methods used in the trials included in the BPLTTC study….. **p 37**
Table S8. Supplementary data for Figure 1 and Figure S2, showing estimated mean blood pressure separately for each comparison arm at specific time points during follow-up, by trial design ………… **p 39**
Table S9. Supplementary data for Figure 2 and Figure S3 showing mean blood pressure difference between comparison groups over follow-up time …………………………………………………………. **p 40**
Table S10. Supplementary data for Figure 2 and Figure S3 showing achieved mean blood pressure difference between active and control groups with and without taking into account early follow-up measurements ………………………………………………………………………………………… **p 41**
Table S11. Achieved mean blood pressure difference between active and control groups stratified by time period of conducting the trial ……………………………………………………………….. **p 42**
Table S12. Comparison of models estimating mean blood pressure difference between comparison arms ………………………………………………………………………………………………. **p 43**

CLINICAL PERSPECTIVES ………………………………………………………………………… **p 44**

TRIAL ACRONYM LEGEND ……………………………………………………………………… **p 45**

REFERENCES ……………………………………………………………………………………… **p 46**

Canoy D, *et al. Heart* 2022; 108:1281–1289. doi: 10.1136/heartjnl-2021-320171

The Blood Pressure Lowering Treatment Trialists' Collaboration

Working Group:
Dexter Canoy,[a,b] Emma Copland,[a,b] Milad Nazarzadeh,[a,b] Rema Ramakrishnan,[c] Ana-Catarina Pinho-Gomes,[d,e] Abdul Salam,[f,g] Jamie P Dwyer,[h] Farshad Farzadfar,[i] Johan Sundström,[j] Mark Woodward,[f,k,l] Barry R Davis,[m] and Kazem Rahimi,[a,b] on behalf of the Blood Pressure Lowering Treatment Trialists' Collaboration*

[a]Deep Medicine, Nuffield Department of Women's and Reproductive Health, University of Oxford, UK
[b]NIHR Oxford Biomedical Research Centre, Oxford University Hospitals NHS Foundation Trust, Oxford, UK
[c]National Perinatal Epidemiology Unit, Nuffield Department of Population Health, University of Oxford, Oxford, UK
[d]School of Population Health & Environmental Sciences, Faculty of Life Sciences & Medicine, King's College London, London, UK
[e]Department of Community Medicine, Centre for Health Technology and Services Research, University of Porto, Porto, Portugal
[f]The George Institute for Global Health, University of New South Wales, Sydney, Australia
[g7]The George Institute for Global Health, Hyderabad, India
[h]Vanderbilt University Medical Center, Nashville, Tennessee, USA
[i]Endocrinology and Metabolism Institute, Tehran University of Medical Sciences, Tehran, Iran
[j]Department of Medical Sciences, Uppsala University, Uppsala, Sweden
[k]The George Institute for Global Health UK, Imperial College London, London, UK
[l]Department of Epidemiology, Johns Hopkins University, Baltimore, Maryland, USA
[m]The University of Texas School of Public Health, Houston, Texas, USA.

Steering Committee:

Kazem Rahimi (Chair) (Nuffield Department of Women's and Reproductive Health, University of Oxford, Oxford, UK), Koon Teo (Population Health Research Institute, McMaster University, Hamilton, Ontario, Canada) Barry R Davis (The University of Texas School of Public Health, Houston, Texas, USA), John Chalmers (The George Institute for Global Health, University of New South Wales, Sydney, Australia), Carl J Pepine (Department of Medicine, University of Florida, Gainesville, Florida, USA).

Collaborating Trialists:

A Adler (UKPDS [UK Prospective Diabetes Study]),
L Agodoa (AASK [African-American Study of Kidney Disease and Hypertension]),
A Algra (Dutch TIA Study [Dutch Transient Ischemic Attack Study]),
F W Asselbergs (PREVEND-IT [Prevention of Renal and Vascular End- stage Disease Intervention Trial]),
N S Beckett (HYVET [Hypertension in the Very Elderly Trial]),
E Berge (deceased) (VALUE trial [Valsartan Antihypertensive Long-term Use Evaluation trial]),
H Black (CONVINCE [Controlled Onset Verapamil Investigation of Cardiovascular End Points]),
F P J Brouwers (PREVEND-IT),
M Brown (INSIGHT [International Nifedipine GITS Study: Intervention as a Goal in Hypertension]),
C J Bulpitt (EWPHE [European Working Party on High Blood Pressure in the Elderly], HYVET),
R P Byington (PREVENT [Prospective Randomized Evaluation of the Vascular Effects of Norvasc Trial]),
J Chalmers (ADVANCE [Action in Diabetes and Vascular Disease: Preterax and Diamicron MR Controlled Evaluation], PROGRESS [Perindopril protection against recurrent stroke]),
W C Cushman (ACCORD [Action to Control Cardiovascular Risk in Diabetes], ALLHAT, SPRINT [Systolic Blood Pressure Intervention Trial]),
J Cutler (ALLHAT [Antihypertensive and Lipid-Lowering Treatment to Prevent Heart Attack Trial]),
B R Davis (ALLHAT),
R B Devereaux (LIFE [Losartan Intervention For Endpoint reduction in hypertension]),
J P Dwyer (IDNT [Irbesartan Diabetic Nephropathy Trial]),
R Estacio (ABCD [Appropriate Blood Pressure Control in Diabetes]),
R Fagard (SYST-EUR [SYSTolic Hypertension in EURope]),
K Fox (EUROPA [European trial on Reduction Of cardiac events with Perindopril among patients with stable coronary Artery disease]),
T Fukui (CASE-J [Candesartan Antihypertensive Survival Evaluation in Japan]),
A K Gupta (ASCOT-BPLA [Anglo-Scandinavian Cardiac Outcomes Trial – Blood Pressure Lowering Arm]),
R R Holman (UKPDS),
Y Imai (HOMED-BP [Hypertension Objective Treatment Based on Measurement by Electrical Devices of Blood Pressure]),

Canoy D, *et al. Heart* 2022; 108:1281–1289. doi: 10.1136/heartjnl-2021-320171

M Ishii (JMIC-B [Japan Multicenter Investigation for Cardiovascular Diseases-B]),
S Julius (VALUE),
Y Kanno (E-COST [Efficacy of Candesartan on Outcome in Saitama Trial]),
S E Kjeldsen (VALUE, LIFE),
J Kostis (SHEP [Systolic Hypertension in the Elderly Program]),
K Kuramoto (NICS-EH [National Intervention Cooperative Study in Elderly Hypertensives]),
J Lanke (STOP Hypertension-2 [Swedish Trial in Old Patients with Hypertension-2], NORDIL [Nordic Diltiazem]),
E Lewis (IDNT),
J B Lewis (IDNT),
M Lievre (DIABHYCAR [Non-insulin-dependent diabetes, hypertension, microalbuminuria or proteinuria, cardiovascular events, and ramipril study]),
L H Lindholm (CAPPP [Captopril Prevention Project], STOP Hypertension-2, NORDIL),
S Lueders (MOSES [The Morbidity and Mortality After Stroke, Eprosartan Compared With Nitrendipine for Secondary Prevention]),
S MacMahon (ADVANCE),
G Mancia (INSIGHT),
M Matsuzaki (COPE [The Combination Therapy of Hypertension to Prevent Cardiovascular Events]),
M H Mehlum (VALUE),
S Nissen (CAMELOT [Comparison of Amlodipine vs Enalapril to Limit Occurrences of Thrombosis]),
H Ogawa (HIJ-CREATE [Heart Institute of Japan Candesartan Randomized Trial for Evaluation in Coronary Heart Disease]),
T Ogihara (CASE-J, COLM [Combinations of OLMesartan], COPE),
T Ohkubo (HOMED-BP),
C R Palmer (INSIGHT),
A Patel (ADVANCE),
C J Pepine (INVEST [International Verapamil SR-Trandolapril Study]),
M A Pfeffer (PEACE [Prevention of Events with Angiotensin- Converting Enzyme Inhibition]),
B Pitt (PREVENT),
N R Poulter (ASCOT [Anglo-Scandinavian Cardiac Outcomes Trial]),
H Rakugi (CASE-J, VALISH [Valsartan in Elderly Isolated Systolic Hypertension Study]),
G Reboldi (Cardio-Sis [CARDIOvascolari del Controllo della Pressione Arteriosa SIStolica]),
C Reid (ANBP2 [The Second Australian National Blood Pressure Study]),
G Remuzzi (BENEDICT [BErgamo NEphrologic DIabetes Complications Trial]),
P Ruggenenti (BENEDICT),
T Saruta (CASE-J),
J Schrader (MOSES),
R Schrier (deceased) (ABCD),
P Sever (ASCOT-BPLA),
P Sleight (deceased) (CONVINCE, HOPE [Heart Outcomes Prevention Evaluation], ONTARGET [Ongoing Telmisartan Alone and in Combination with Ramipril Global Endpoint Trial], TRANSCEND [Telmisartan Randomized AssessmeNt Study in ACE iNtolerant subjects with cardiovascular Disease]),
J A Staessen (SYST-EUR),
H Suzuki (E-COST),
L Thijs (SYST-EUR),
K Ueshima (CASE-J, VALISH),
S Umemoto (COPE),
W H van Gilst (PREVEND-IT),
P Verdecchia (Cardio-Sis),
K Wachtell (LIFE),
P Whelton (SPRINT),
L Wing (ANBP2),
M Woodward (ADVANCE, PROGRESS),
Y Yui (JMIC-B),
S Yusuf (HOPE, ONTARGET, PRoFESS, TRANSCEND),
A Zanchetti (deceased) (ELSA [European Lacidipine Study on Atherosclerosis], VHAS [Verapamil in Hypertension and Atherosclerosis Study])
Z Y Zhang (SYST-EUR)

Other members: C Anderson, C Baigent, BM Brenner, R Collins, D de Zeeuw, J Lubsen, E Malacco, B Neal, V Perkovic, A Rodgers, P Rothwell, G Salimi-Khorshidi, J Sundström, F Turnbull, G Viberti, J Wang

Canoy D, *et al. Heart* 2022; 108:1281–1289. doi: 10.1136/heartjnl-2021-320171

Supplementary Methods

Comparison groups

For trials that compared drug(s) with placebo, we assigned those in the drug(s) and placebo arms to active and control groups, respectively. For interventions that compared effects based on blood pressure (BP)-lowering targets (e.g. more versus less intense treatment to reduce baseline BP below a pre-specified threshold or to achieve a pre-defined magnitude of reduction in BP), participants allocated to treatment arms aiming to achieve greater BP reduction were assigned to the active group, and those allocated to achieve less reduction to the control group. Both these placebo-controlled trials and trials comparing effects based on BP reduction intensity were also collectively classified as '*BP difference*' trials. Trials that compared effects between different drug classes on clinical outcomes were classified as '*drug class comparison*' trials. For these trials, we retrospectively assigned treatment allocations for the drug class achieving greater BP reduction to the active group; where the difference was null, we assigned treatment arms to the active group for those randomised to receive the novel or newer drug class and to the control group if randomised to receive standard or usual drug class therapy.

Estimating follow-up BP level and difference between comparison arms in trials without follow-up measurements

Individual-level follow-up BP measurements were not accessible to the collaboration for two trials (IDNT[1] and Cardio-SiS[2]), and baseline BP measurements were also not available for IDNT Mean BP values at baseline for IDNT were extracted from published literature for the treatment and control groups. For each participant of this trial, the baseline BP was imputed as the mean value for the group they were randomised to. For both Cardio-Sis and IDNT, the mean follow-up BP values at the end of the trials in the treatment and control groups were extracted from published literature. These mean values were imputed as the follow-up BP values for the relevant groups at one-year intervals for the length of the study duration. For the sensitivity analysis investigating BP difference achieved by individual trials, we extracted data on the mean BP difference (and 95% confidence interval) between treatment and control groups also from the published literature for Cardio-Sis. As such data were not available for IDNT, we estimated these values by using simple linear regression models, with follow-up BP as the outcome, and baseline BP, treatment group, age and gender as covariates.

Canoy D, *et al. Heart* 2022; 108:1281–1289. doi: 10.1136/heartjnl-2021-320171

Model development and selection

Because the BPLTTC has individual participant-level data, we therefore made use of the full information on individual-level BP measurements in the dataset for characterising longitudinal patterns of BP, and estimating BP difference between comparison groups. Using linear mixed models, we compared models to determine potential sources of heterogeneity, and checked residual plots to help assessthe best fitting model for our data.[3-6] Figure S1 shows the scheme for model specification and selection, using likelihood ratio test (for nested models) and the Akaike information criterion (for non-nested models), and implemented using the *nlme* package in R. The model based on random intercepts (trial and participant), fixed treatment effect and random time (participant) fitted the data better than the base model (no treatment effect), or models with random treatment effects or fixed time effect. Further model assessment showed that the best fitting model included specifying a polynomial term for time and adjusting for baseline BP, age and sex, which formed the basis for our final model as shown in Equation (1). Residual plots showed no apparent non-random patterns and indicated a good fit of the final model. We then implemented this model, and modified as appropriate, in subsequent analyses.

$$BP_{tij} = [\Upsilon_{000} + \Upsilon_{100}(time)_{tij}] + [r_{0ij} + r_{1i}(time)_{tij}] + \beta1(treatment) + \beta2(time) + \beta3(time)^2 + \beta4(time)^3 + e_{ij} \quad (1)$$

where:

i=individual

j=trial

BP_{tij} =blood pressure outcome

Υ_{000}=grand mean of the intercept over all individuals and group

$\Upsilon_{100}(time)_{tij}$ =grand mean slope over all individuals and groups

r_{0ij} =random intercept (trial and individual)

$r_{1i}(time)_{ti}$ =random slope (individual)

Estimating follow-up BP level and difference between comparison arms from the linear mixed models

How to precisely measure differences in BP between treatment arms across different trials is not clear. For example, BP differences have been based on measures taken at a fixed timepoint during follow-up,[7] the average of all follow-up measures,[8-10] or the final follow-up measures.[11] In our analysis, we first examined how BP differed between treatment arms across specific time points during follow-up. To estimate this blood pressure difference, we first split the data into specific time periods, and ran models on each time period separately. In developing the best-fitting model for the data used in these specific analyses, Equation (1) was modified by retaining a random intercept for

Canoy D, *et al. Heart* 2022; 108:1281–1289. doi: 10.1136/heartjnl-2021-320171

individuals but excluded a random intercept for trials and random slope for time. To investigate how BP differed across all follow-up periods across all participants, by patient subgroups and trial design, we used Equation (1) to estimate this difference by showing analyses that included all follow-up BP measurements as well as by excluding follow-up measures taken <12 months from baseline, and modified the polynomial term for follow-up time accordingly. We have also illustrated how our model that took into account various potential sources of heterogeneity compared with a conventional approach based on simply obtaining the difference in BP change from baseline and follow-up between treatment arms, which assumes fixed effects for treatment over time (both at trial and participant levels) albeit allowing for random intercept at trial level, in a sensitivity analysis.

Canoy D, *et al. Heart* 2022; 108:1281–1289. doi: 10.1136/heartjnl-2021-320171

Figure S1. Schematic diagram for model development and comparison.

Model 0 (base model)
Random intercepts (trial and individual levels*)
No treatment effect
Fixed time effect (linear)

**Trial and individual levels tested separately and together; all models compared with each other as well as with model without random intercepts; best fitting model included random intercepts for both trial and individual levels*

↓

Model 1
Random intercepts (trial and individual levels)
Fixed treatment effect
Fixed time effect (linear)

Model 1 vs model 2: Treatment effect significant

↓

Model 2
Random intercepts (trial and individual levels)
Fixed treatment effect
Fixed time effect (non-linear or polynomial* transformation for time)

Model 2 vs 1: Cubic transformation for time significant
**Models using quadratic and cubic transformation for time were tested separately; model that included cubic term for time fitted better than with its quadratic transformation. However, for some analyses, this polynomial time transformation was not necessary.*

↓

Model 3
Random intercepts (trial and individual levels)
Fixed treatment effect
Fixed time effect (cubic term)
Random slope for time (individual level*)

Model 3 vs 2: Random slope for time at individual level significant
**Models tested for trial and individual levels separately and together; best fitting model included random slope for time at individual level*

↓

Model 4
Random intercepts (trial and individual levels)
Fixed time effect (cubic term)
Random slope for time (individual-level)
Fixed treatment effect
Random slope for treatment effect

Lack of convergence for models that included random slope for treatment effect (trial and individual levels)

FINAL MODEL: ***Random intercepts (trial and individual levels), fixed treatment effect, fixed time effect (cubic term) and random slope for time (individual level) (Model 3)***

Canoy D, *et al. Heart* 2022; 108:1281–1289. doi: 10.1136/heartjnl-2021-320171

Figure S2. Blood pressure trajectories for all blood pressure difference trials (blood pressure-lowering intensity and placebo-controlled trials combined). Results are in red for active group and black for control group, from three months to five years of follow-up. Estimates based on separate models for treatment and control groups, with random intercepts at individual and trial levels, a random slope for time at the individual level (see ***Method*** for details) and adjusted for baseline blood pressure, age and sex. Baseline systolic/diastolic blood pressure for active and control groups was 147/84 mmHg. Estimated blood pressure at specific time points shown in online ***Table S8***.

A. Systolic blood pressure

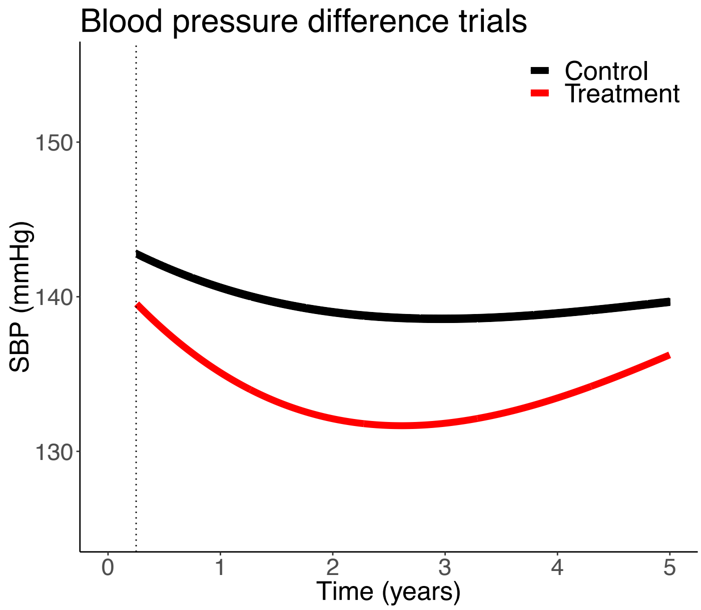

B. Diastolic blood pressure

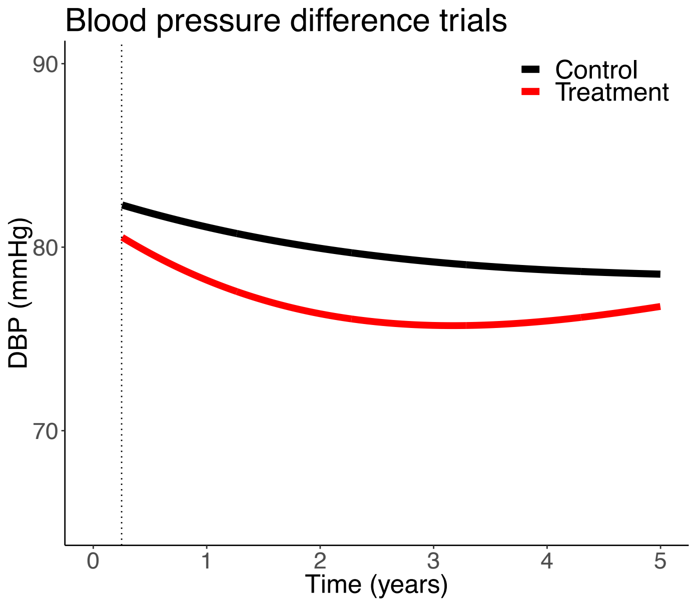

Canoy D, *et al. Heart* 2022; 108:1281–1289. doi: 10.1136/heartjnl-2021-320171

Figure S3. Effects of blood pressure-lowering treatment on mean blood pressure at fixed follow-up time points and across all follow-up period for all blood pressure difference trials (blood pressure-lowering intensity and placebo-controlled trials combined). For mean difference at fixed follow-up time periods, estimates based on separate models for each time period with a fixed treatment effect and random intercept for individuals. For mean difference achieved across all time period (showing results based on all follow-up blood pressure measures and measures obtained from 12 months until end of follow-up), estimates based on fixed treatment effect and random intercepts at individual and trial levels, a random slope for time at the individual level. All mean difference values were adjusted for baseline blood pressure, age and sex. The area of the square is inversely proportional to the variance of the estimated difference. Negative values indicate lower blood pressure in the active than in the control group. Additional information provided in online ***Table S9*** and ***Table S10***.

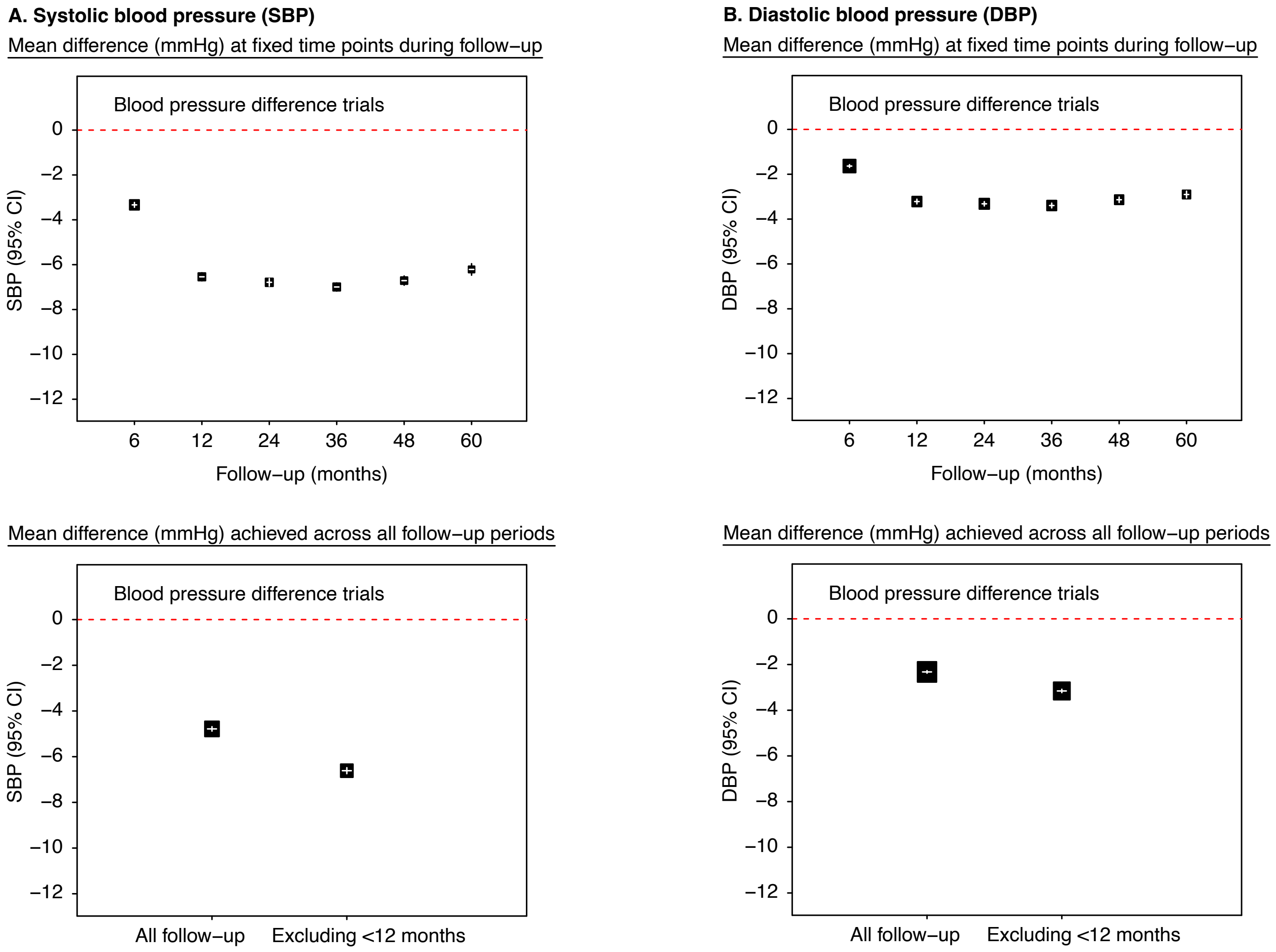

Canoy D, *et al. Heart* 2022; 108:1281–1289. doi: 10.1136/heartjnl-2021-320171

Figure S4. Effects of blood pressure-lowering treatment on long-term mean blood pressure, by baseline characteristics in all blood pressure difference trials (blood pressure-lowering intensity and placebo-controlled trials combined). Estimates based on fixed treatment effect and random intercepts at individual and trial levels, a random slope for time at the individual level (see ***Method*** for details) and adjusted for baseline blood pressure, age and sex except when these variables are used as stratification factors

A. Mean systolic blood pressure difference

All blood pressure difference trials

	N participants (N trials)	SBP difference (mmHg) (95% CI)
ALL	148,873 (30)	−6.6 (−6.7, −6.5)
By baseline characteristics		
**1. SBP (mmHg)**		
Less than 120	10,650 (24)	−5.7 (−6.1, −5.2)
120–129	18,448 (24)	−5.9 (−6.3, −5.6)
130–139	26,077 (24)	−6.2 (−6.5, −6.0)
140–149	27,386 (24)	−5.9 (−6.2, −5.6)
150–159	22,237 (28)	−5.8 (−6.1, −5.4)
160 or higher	43,974 (29)	−8.3 (−8.6, −8.0)
	Heterogeneity:	χ^2_1 = 369.8; P<0.0001
**2. Age (years)**		
Less than 50	11,880 (21)	−7.3 (−7.8, −6.9)
50–64	61,365 (27)	−6.8 (−7.0, −6.6)
65–79	64,228 (29)	−6.0 (−6.2, −5.8)
80 or higher	11,362 (19)	−8.5 (−9.1, −8.0)
	Heterogeneity:	χ^2_1 = 6.7; P=0.010
**3. Sex**		
Men	92,844 (30)	−7.3 (−7.6, −7.1)
Women	56,029 (30)	−6.2 (−6.3, −6.0)
	Heterogeneity:	χ^2_1 = 71.5; P<0.0001
**4. Body mass index (kg/m^2)**		
Less than 25	38,492 (27)	−6.4 (−6.6, −6.1)
25–29	53,960 (27)	−6.1 (−6.4, −5.9)
30 or higher	35,093 (27)	−7.3 (−7.6, −7.1)
	Heterogeneity:	χ^2_1 = 2; P=0.156
**5. Cardiovascular disease**		
No	68,128 (21)	−5.0 (−5.1, −4.8)
Yes	77,826 (28)	−8.7 (−8.8, −8.5)
	Heterogeneity:	χ^2_1 = 734.8; P<0.0001
**6. Diabetes**		
No	91,912 (22)	−5.4 (−5.6, −5.2)
Yes	44,719 (25)	−7.3 (−7.5, −7.1)
	Heterogeneity:	χ^2_1 = 172.5; P<0.0001
**7. Antihypertensive drug use**		
No	53,726 (28)	−5.3 (−5.5, −5.1)
Yes	85,974 (28)	−7.6 (−7.9, −7.4)
	Heterogeneity:	χ^2_1 = 268.8; P<0.0001

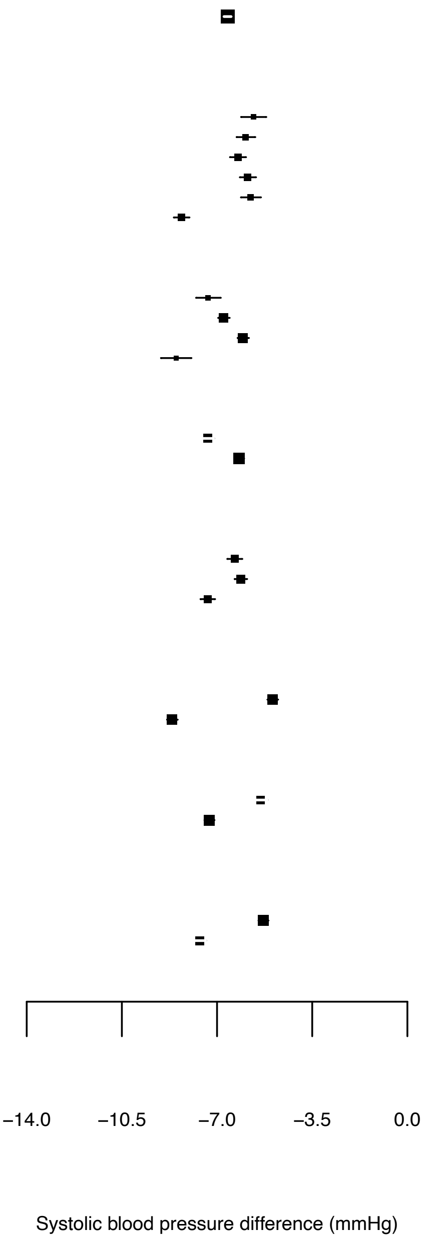

10

Figure S4. Effects of blood pressure-lowering treatment on long-term mean blood pressure (cont'd)
B. Mean diastolic blood pressure difference

All blood pressure difference trials

	N participants (N trials)	DBP difference (mmHg) (95% CI)
ALL	148,873 (30)	–3.2 (–3.2, –3.1)
By baseline characteristics		
**1. DBP (mmHg)**		
Less than 70	13,843 (25)	–3.0 (–3.3, –2.7)
70–79	33,002 (26)	–3.0 (–3.2, –2.9)
80–89	52,331 (26)	–2.7 (–2.9, –2.6)
90–99	34,324 (29)	–3.4 (–3.6, –3.2)
100–109	12,264 (25)	–4.2 (–4.5, –3.9)
110 or higher	2994 (22)	–5.9 (–6.6, –5.2)
	Heterogeneity:	χ^2_1 = 204.2; P<0.0001
**2. Age (years)**		
Less than 50	11,880 (21)	–4.1 (–4.4, –3.9)
50–64	61,365 (27)	–3.3 (–3.5, –3.2)
65–79	64,228 (29)	–2.7 (–2.8, –2.6)
80 or higher	11,362 (19)	–3.6 (–3.9, –3.3)
	Heterogeneity:	χ^2_1 = 99.5; P<0.0001
**3. Sex**		
Men	92,844 (30)	–3.1 (–3.2, –3.0)
Women	56,029 (30)	–3.3 (–3.4, –3.2)
	Heterogeneity:	χ^2_1 = 10.8; P=0.001
**4. Body mass index (kg/m^2)**		
Less than 25	38,492 (27)	–2.8 (–3.0, –2.7)
25–29	53,960 (27)	–2.9 (–3.0, –2.7)
30 or higher	35,093 (27)	–3.6 (–3.8, –3.5)
	Heterogeneity:	χ^2_1 = 3.9; P=0.048
**5. Cardiovascular disease**		
No	68,128 (21)	–2.4 (–2.5, –2.3)
Yes	77,826 (28)	–4.0 (–4.1, –3.9)
	Heterogeneity:	χ^2_1 = 434.1; P<0.0001
**6. Diabetes**		
No	91,912 (22)	–2.5 (–2.6, –2.4)
Yes	44,719 (25)	–3.6 (–3.7, –3.5)
	Heterogeneity:	χ^2_1 = 185.5; P<0.0001
**7. Antihypertensive drug use**		
No	53,726 (28)	–2.5 (–2.6, –2.4)
Yes	85,974 (28)	–3.7 (–3.8, –3.5)
	Heterogeneity:	χ^2_1 = 220.3; P<0.0001

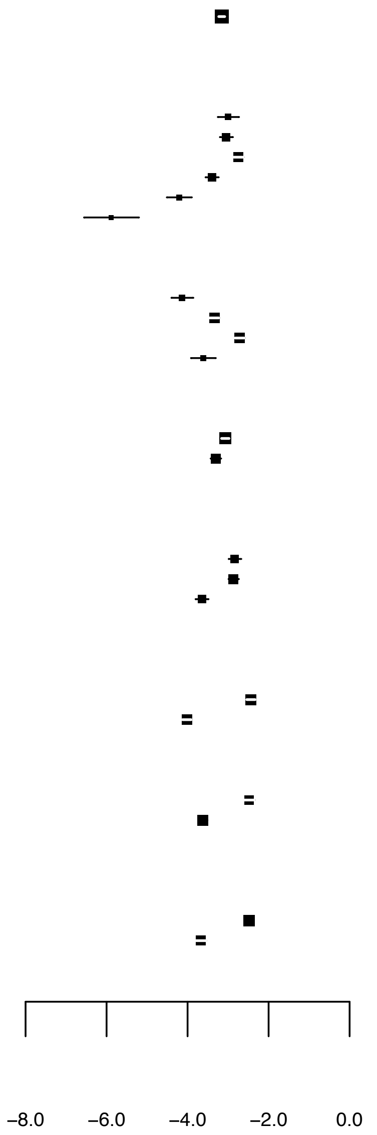

Diastolic blood pressure difference (mmHg)

Figure S5. Effects of blood pressure-lowering treatment on long-term blood pressure for each trial. Estimates based on fixed treatment effect, a random intercept at the individual level, a random slope for time at the individual level (excluded from some trials due to non-convergence) (see ***Method*** for details) and adjusted for baseline blood pressure, age and sex. Acronyms described in full in ***Trial acronym legend*** in the Supplement.

A. Mean systolic blood pressure difference

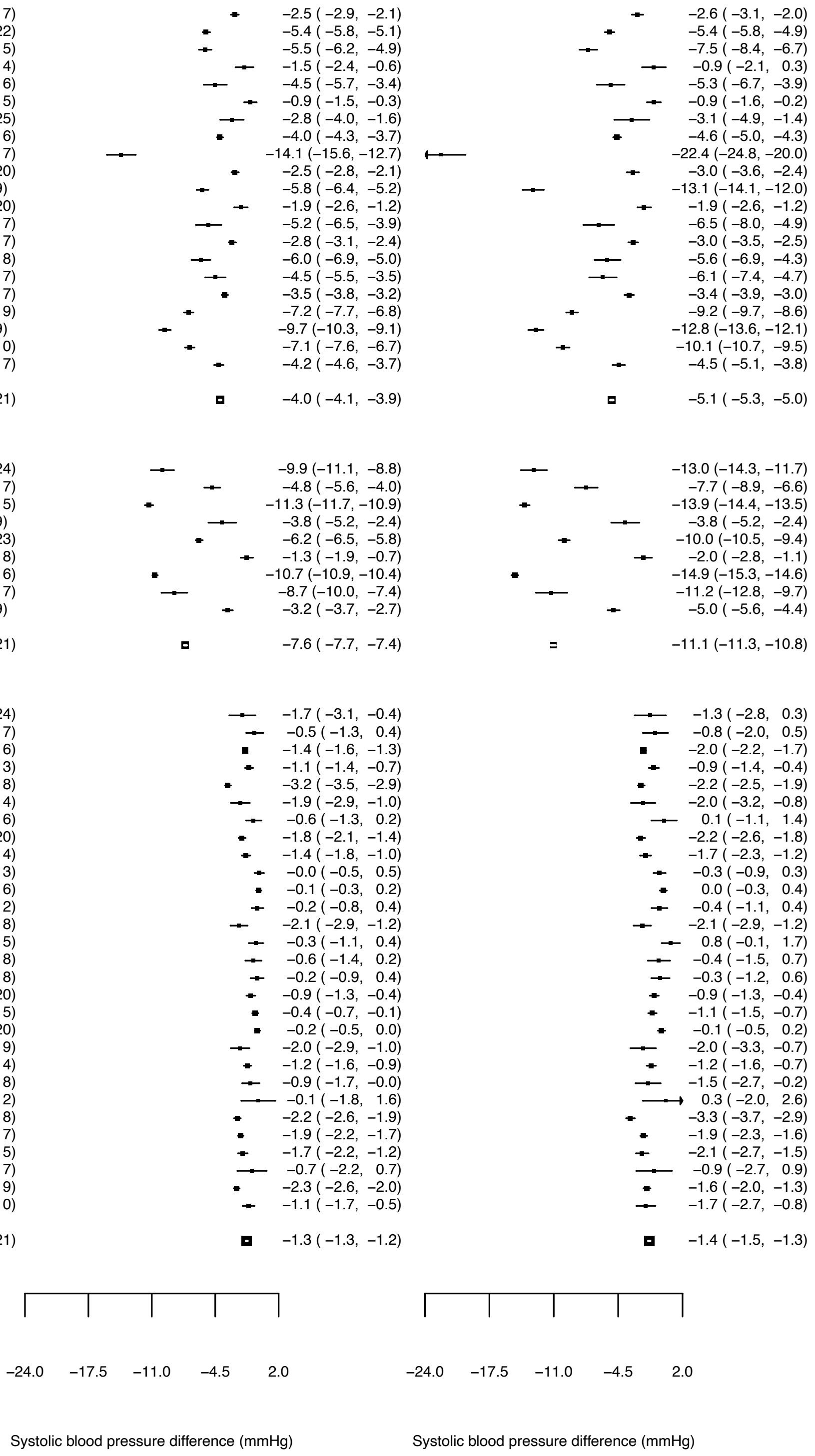

Trials	No. of participants Treatment / Control	Baseline SBP (SD)	SBP difference (mmHg) (95% CI) including total follow–up period	SBP difference (mmHg) (95% CI) excluding first 12 months
Placebo–controlled trials				
ACTIVE I	4518 / 4498	138 (17)	−2.5 (−2.9, −2.1)	−2.6 (−3.1, −2.0)
ADVANCE	5569 / 5571	145 (22)	−5.4 (−5.8, −5.1)	−5.4 (−5.8, −4.9)
ANBP	1721 / 1706	157 (15)	−5.5 (−6.2, −4.9)	−7.5 (−8.4, −6.7)
BENEDICT	904 / 300	151 (14)	−1.5 (−2.4, −0.6)	−0.9 (−2.1, 0.3)
CAMELOT	1336 / 655	129 (16)	−4.5 (−5.7, −3.4)	−5.3 (−6.7, −3.9)
DIABHYCAR	2443 / 2469	145 (15)	−0.9 (−1.5, −0.3)	−0.9 (−1.6, −0.2)
Dutch TIA	732 / 741	157 (25)	−2.8 (−4.0, −1.6)	−3.1 (−4.9, −1.4)
EUROPA	6110 / 6108	137 (16)	−4.0 (−4.3, −3.7)	−4.6 (−5.0, −4.3)
EWPHE	416 / 424	183 (17)	−14.1 (−15.6, −12.7)	−22.4 (−24.8, −20.0)
HOPE	4645 / 4652	139 (20)	−2.5 (−2.8, −2.1)	−3.0 (−3.6, −2.4)
HYVET	1933 / 1912	173 (9)	−5.8 (−6.4, −5.2)	−13.1 (−14.1, −12.0)
IDNT	1146 / 569	159 (20)	−1.9 (−2.6, −1.2)	−1.9 (−2.6, −1.2)
PART−2	308 / 309	133 (17)	−5.2 (−6.5, −3.9)	−6.5 (−8.0, −4.9)
PEACE	4158 / 4132	133 (17)	−2.8 (−3.1, −2.4)	−3.0 (−3.5, −2.5)
PREVEND IT	431 / 433	130 (18)	−6.0 (−6.9, −5.0)	−5.6 (−6.9, −4.3)
PREVENT	417 / 408	129 (17)	−4.5 (−5.5, −3.5)	−6.1 (−7.4, −4.7)
PROFESS	9873 / 9925	144 (17)	−3.5 (−3.8, −3.2)	−3.4 (−3.9, −3.0)
PROGRESS	3051 / 3054	147 (19)	−7.2 (−7.7, −6.8)	−9.2 (−9.7, −8.6)
SHEP	2365 / 2371	170 (9)	−9.7 (−10.3, −9.1)	−12.8 (−13.6, −12.1)
Syst−Eur	2398 / 2297	174 (10)	−7.1 (−7.6, −6.7)	−10.1 (−10.7, −9.5)
TRANSCEND	2954 / 2972	141 (17)	−4.2 (−4.6, −3.7)	−4.5 (−5.1, −3.8)
All placebo−controlled trials	57,430 / 55,504	145 (21)	−4.0 (−4.1, −3.9)	−5.1 (−5.3, −5.0)
BP−lowering intensity trials				
AASK	540 / 554	150 (24)	−9.9 (−11.1, −8.8)	−13.0 (−14.3, −11.7)
ABCD	474 / 476	155 (17)	−4.8 (−5.6, −4.0)	−7.7 (−8.9, −6.6)
ACCORD	2362 / 2371	139 (15)	−11.3 (−11.7, −10.9)	−13.9 (−14.4, −13.5)
Cardio−Sis	558 / 553	158 (9)	−3.8 (−5.2, −2.4)	−3.8 (−5.2, −2.4)
HDFP	5553 / 5387	159 (23)	−6.2 (−6.5, −5.8)	−10.0 (−10.5, −9.4)
HOMED−BP	1759 / 1759	154 (18)	−1.3 (−1.9, −0.7)	−2.0 (−2.8, −1.1)
SPRINT	4678 / 4683	140 (16)	−10.7 (−10.9, −10.4)	−14.9 (−15.3, −14.6)
UKPDS	758 / 390	159 (17)	−8.7 (−10.0, −7.4)	−11.2 (−12.8, −9.7)
VALISH	1545 / 1534	169 (9)	−3.2 (−3.7, −2.7)	−5.0 (−5.6, −4.4)
All BP−lowering intensity trials	18,227 / 17,707	151 (21)	−7.6 (−7.7, −7.4)	−11.1 (−11.3, −10.8)
Drug class comparison trials				
AASK	653 / 441	150 (24)	−1.7 (−3.1, −0.4)	−1.3 (−2.8, 0.3)
ABCD	235 / 235	155 (17)	−0.5 (−1.3, 0.4)	−0.8 (−2.0, 0.5)
ALLHAT	15,255 / 27,163	146 (16)	−1.4 (−1.6, −1.3)	−2.0 (−2.2, −1.7)
ANBP2	3039 / 3044	168 (13)	−1.1 (−1.4, −0.7)	−0.9 (−1.4, −0.4)
ASCOT−BPLA	9639 / 9618	164 (18)	−3.2 (−3.5, −2.9)	−2.2 (−2.5, −1.9)
BENEDICT	602 / 303	151 (14)	−1.9 (−2.9, −1.0)	−2.0 (−3.2, −0.8)
CAMELOT	663 / 673	129 (16)	−0.6 (−1.3, 0.2)	0.1 (−1.1, 1.4)
CAPPP	5493 / 5492	161 (20)	−1.8 (−2.1, −1.4)	−2.2 (−2.6, −1.8)
CASE−J	2349 / 2354	163 (14)	−1.4 (−1.8, −1.0)	−1.7 (−2.3, −1.2)
COLM	2568 / 2573	158 (13)	−0.0 (−0.5, 0.5)	−0.3 (−0.9, 0.3)
CONVINCE	8179 / 8297	150 (16)	−0.1 (−0.3, 0.2)	0.0 (−0.3, 0.4)
COPE	2183 / 1110	154 (12)	−0.2 (−0.8, 0.4)	−0.4 (−1.1, 0.4)
E−COST	1053 / 995	164 (18)	−2.1 (−2.9, −1.2)	−2.1 (−2.9, −1.2)
ELSA	1177 / 1157	160 (15)	−0.3 (−1.1, 0.4)	0.8 (−0.1, 1.7)
HIJ−CREATE	1024 / 1025	135 (18)	−0.6 (−1.4, 0.2)	−0.4 (−1.5, 0.7)
HOMED−BP	2347 / 1171	154 (18)	−0.2 (−0.9, 0.4)	−0.3 (−1.2, 0.6)
IDNT	579 / 567	160 (20)	−0.9 (−1.3, −0.4)	−0.9 (−1.3, −0.4)
INSIGHT	3164 / 3157	173 (15)	−0.4 (−0.7, −0.1)	−1.1 (−1.5, −0.7)
INVEST	10,648 / 10,672	151 (20)	−0.2 (−0.5, 0.0)	−0.1 (−0.5, 0.2)
JMIC−B	828 / 822	146 (19)	−2.0 (−2.9, −1.0)	−2.0 (−3.3, −0.7)
LIFE	4605 / 4588	174 (14)	−1.2 (−1.6, −0.9)	−1.2 (−1.6, −0.7)
MOSES	671 / 681	151 (18)	−0.9 (−1.7, −0.0)	−1.5 (−2.7, −0.2)
NICS−EH	204 / 210	172 (12)	−0.1 (−1.8, 1.6)	0.3 (−2.0, 2.6)
NORDIL	5471 / 5410	173 (18)	−2.2 (−2.6, −1.9)	−3.3 (−3.7, −2.9)
ONTARGET	8502 / 17,118	142 (17)	−1.9 (−2.2, −1.7)	−1.9 (−2.3, −1.6)
STOP HPN−2	2213 / 4401	194 (15)	−1.7 (−2.2, −1.2)	−2.1 (−2.7, −1.5)
UKPDS	400 / 358	159 (17)	−0.7 (−2.2, 0.7)	−0.9 (−2.7, 0.9)
VALUE	7596 / 7649	154 (19)	−2.3 (−2.6, −2.0)	−1.6 (−2.0, −1.3)
VHAS	707 / 707	169 (10)	−1.1 (−1.7, −0.5)	−1.7 (−2.7, −0.8)
All drug class comparison trials	100,972 / 123,066	156 (21)	−1.3 (−1.3, −1.2)	−1.4 (−1.5, −1.3)

Canoy D, *et al. Heart* 2022; 108:1281–1289. doi: 10.1136/heartjnl-2021-320171

Figure S5. Effects of blood pressure-lowering treatment on long-term blood pressure for each trial (cont'd).
B. Mean diastolic blood pressure difference

Trials	No. of participants Treatment / Control	Baseline DBP (SD)	DBP difference (mmHg) (95% CI) including total follow−up period	DBP difference (mmHg) (95% CI) excluding first 12 months
Placebo−controlled trials				
ACTIVE I	4518 / 4498	82 (10)	−1.5 (−1.7, −1.2)	−1.4 (−1.7, −1.1)
ADVANCE	5569 / 5571	81 (10)	−2.2 (−2.4, −2.0)	−2.1 (−2.3, −1.9)
ANBP	1721 / 1706	100 (10)	−2.8 (−3.1, −2.4)	−3.8 (−4.3, −3.3)
BENEDICT	904 / 300	88 (10)	−1.3 (−1.8, −0.8)	−1.3 (−1.9, −0.6)
CAMELOT	1336 / 655	78 (10)	−2.8 (−3.4, −2.1)	−3.3 (−4.2, −2.5)
DIABHYCAR	2443 / 2469	82 (10)	−0.4 (−0.7, −0.0)	−0.4 (−0.8, −0.1)
Dutch TIA	732 / 741	91 (10)	−1.7 (−2.3, −1.1)	−2.0 (−2.9, −1.2)
EUROPA	6110 / 6108	82 (10)	−1.9 (−2.1, −1.8)	−2.2 (−2.4, −2.0)
EWPHE	416 / 424	101 (10)	−6.0 (−6.7, −5.3)	−9.5 (−10.6, −8.4)
HOPE	4645 / 4652	79 (10)	−1.2 (−1.4, −1.0)	−1.4 (−1.8, −1.1)
HYVET	1933 / 1912	91 (10)	−2.8 (−3.1, −2.5)	−5.1 (−5.7, −4.6)
IDNT	1146 / 569	87 (11)	−2.8 (−3.0, −2.7)	−2.8 (−3.0, −2.7)
PART−2	308 / 309	79 (10)	−2.9 (−3.6, −2.1)	−3.6 (−4.5, −2.7)
PEACE	4158 / 4132	77 (10)	−1.4 (−1.6, −1.2)	−1.5 (−1.8, −1.2)
PREVEND IT	431 / 433	76 (10)	−3.3 (−3.8, −2.8)	−2.8 (−3.5, −2.1)
PREVENT	417 / 408	79 (10)	−2.5 (−3.0, −1.9)	−3.3 (−4.0, −2.6)
PROFESS	9873 / 9925	84 (11)	−1.9 (−2.0, −1.7)	−1.8 (−2.0, −1.6)
PROGRESS	3051 / 3054	86 (10)	−3.2 (−3.4, −2.9)	−4.0 (−4.3, −3.7)
SHEP	2365 / 2371	77 (10)	−3.2 (−3.5, −2.9)	−4.2 (−4.6, −3.9)
Syst−Eur	2398 / 2297	86 (10)	−2.9 (−3.2, −2.7)	−4.0 (−4.3, −3.7)
TRANSCEND	2954 / 2972	82 (10)	−2.2 (−2.5, −2.0)	−2.2 (−2.5, −1.8)
All placebo−controlled trials	57,430 / 55,504	83 (11)	−1.9 (−2.0, −1.8)	−2.3 (−2.4, −2.2)
BP−lowering intensity trials				
AASK	540 / 554	95 (10)	−6.5 (−7.3, −5.8)	−7.9 (−8.6, −7.1)
ABCD	474 / 476	98 (10)	−4.5 (−4.9, −4.1)	−6.9 (−7.4, −6.3)
ACCORD	2362 / 2371	76 (10)	−4.8 (−5.1, −4.6)	−6.2 (−6.5, −5.9)
Cardio−Sis	558 / 553	87 (10)	−1.5 (−2.4, −0.6)	−1.5 (−2.4, −0.6)
HDFP	5553 / 5387	101 (9)	−3.1 (−3.3, −2.9)	−5.0 (−5.3, −4.7)
HOMED−BP	1759 / 1759	90 (10)	−0.5 (−0.9, −0.1)	−0.9 (−1.5, −0.4)
SPRINT	4678 / 4683	78 (10)	−4.8 (−5.0, −4.6)	−7.7 (−7.9, −7.4)
UKPDS	758 / 390	93 (10)	−4.1 (−4.8, −3.4)	−5.2 (−6.0, −4.3)
VALISH	1545 / 1534	82 (10)	−0.8 (−1.1, −0.5)	−1.8 (−2.2, −1.3)
All BP−lowering intensity trials	18,227 / 17,707	88 (15)	−3.7 (−3.8, −3.6)	−5.6 (−5.7, −5.4)
Drug class comparison trials				
AASK	653 / 441	95 (10)	0.1 (−0.7, 0.9)	0.1 (−0.8, 1.1)
ABCD	235 / 235	98 (10)	0.1 (−0.3, 0.6)	−0.2 (−0.9, 0.5)
ALLHAT	15,255 / 27,163	84 (10)	0.1 (−0.0, 0.2)	0.1 (−0.1, 0.2)
ANBP2	3039 / 3044	91 (10)	−0.1 (−0.3, 0.1)	−0.0 (−0.3, 0.2)
ASCOT−BPLA	9639 / 9618	95 (10)	−1.8 (−1.9, −1.6)	−2.0 (−2.1, −1.8)
BENEDICT	602 / 303	88 (10)	−0.8 (−1.4, −0.3)	−1.0 (−1.8, −0.3)
CAMELOT	663 / 673	78 (10)	−0.1 (−0.6, 0.4)	0.4 (−0.4, 1.2)
CAPPP	5493 / 5492	99 (10)	−1.1 (−1.2, −0.9)	−1.3 (−1.5, −1.1)
CASE−J	2349 / 2354	92 (10)	−0.8 (−1.1, −0.5)	−0.9 (−1.3, −0.5)
COLM	2568 / 2573	87 (10)	0.4 (0.1, 0.7)	0.4 (−0.1, 0.8)
CONVINCE	8179 / 8297	87 (10)	−0.6 (−0.7, −0.4)	−0.7 (−0.9, −0.5)
COPE	2183 / 1110	89 (10)	0.3 (−0.1, 0.6)	0.4 (−0.2, 0.9)
E−COST	1053 / 995	94 (10)	−0.4 (−1.0, 0.2)	−0.4 (−1.0, 0.2)
ELSA	1177 / 1157	98 (10)	0.1 (−0.3, 0.5)	0.4 (−0.1, 0.9)
HIJ−CREATE	1024 / 1025	76 (10)	−0.5 (−1.0, 0.0)	−0.5 (−1.2, 0.2)
HOMED−BP	2347 / 1171	90 (10)	0.1 (−0.3, 0.5)	0.2 (−0.4, 0.8)
IDNT	579 / 567	87 (11)	0.0 (−0.2, 0.2)	0.0 (−0.2, 0.2)
INSIGHT	3164 / 3157	99 (10)	0.6 (0.4, 0.8)	0.9 (0.7, 1.1)
INVEST	10,648 / 10,672	87 (10)	−0.1 (−0.2, 0.0)	−0.2 (−0.4, 0.0)
JMIC−B	828 / 822	82 (10)	−1.3 (−1.9, −0.8)	−1.5 (−2.3, −0.7)
LIFE	4605 / 4588	98 (10)	1.1 (0.9, 1.3)	0.5 (0.3, 0.8)
MOSES	671 / 681	87 (10)	−0.4 (−0.9, 0.1)	−0.5 (−1.2, 0.1)
NICS−EH	204 / 210	94 (10)	0.5 (−0.5, 1.5)	0.7 (−0.5, 2.0)
NORDIL	5471 / 5410	106 (10)	0.0 (−0.1, 0.2)	−0.1 (−0.3, 0.1)
ONTARGET	8502 / 17,118	82 (10)	−1.1 (−1.2, −0.9)	−1.0 (−1.2, −0.8)
STOP HPN−2	2213 / 4401	98 (10)	−0.7 (−0.9, −0.4)	−0.3 (−0.6, 0.0)
UKPDS	400 / 358	93 (10)	−1.0 (−1.8, −0.3)	−1.2 (−2.2, −0.3)
VALUE	7596 / 7649	87 (10)	−1.6 (−1.7, −1.4)	−1.3 (−1.5, −1.1)
VHAS	707 / 707	102 (10)	0.1 (−0.2, 0.5)	0.2 (−0.3, 0.8)
All drug class comparison trials	100,972 / 123,066	89 (12)	−0.5 (−0.5, −0.5)	−0.6 (−0.7, −0.6)

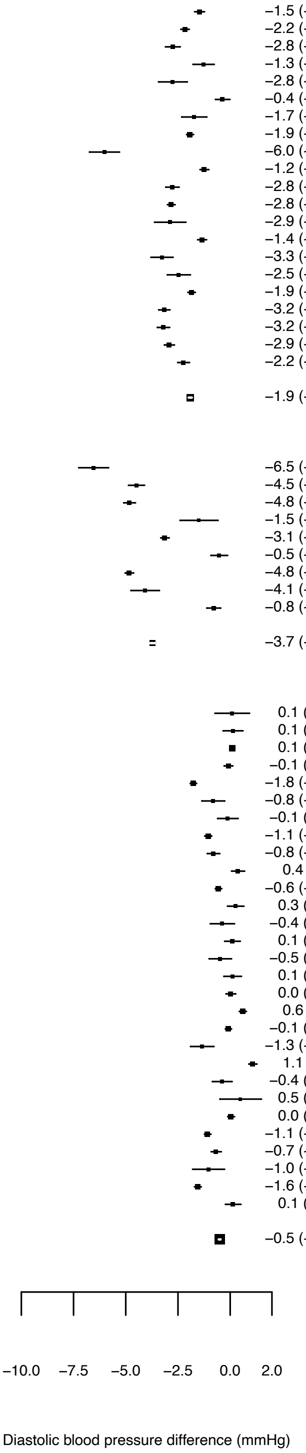

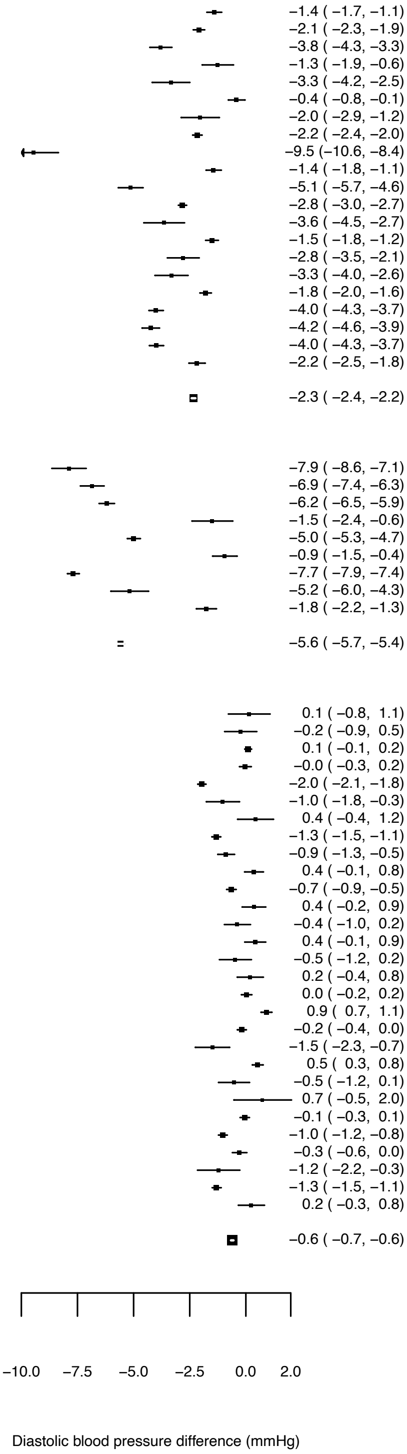

13

Figure S6. Effects of blood pressure-lowering treatment on long-term mean blood pressure after excluding one trial at a time. The estimate shown alongside a trial reflects the blood pressure difference after excluding that particular trial. Estimates based on fixed treatment effect, random intercepts at individual and trial levels, a random slope for time at the individual level (see ***Metho***d for details) and adjusted for baseline blood pressure, age and sex and using all follow-up blood pressure measurements. Acronyms described in full in ***Trial acronym legend*** in the Supplement.

Trials	No. of participants Treatment / Control	Baseline SBP (SD)	SBP difference (mmHg) (95% CI)	Baseline DBP (SD)	DBP difference (mmHg) (95% CI)
Placebo–controlled trials					
ACTIVE I	4518 / 4498	138 (17)	−4.2 (−4.3, −4.0)	82 (10)	−1.9 (−2.0, −1.9)
ADVANCE	5569 / 5571	145 (22)	−3.7 (−3.8, −3.6)	81 (10)	−1.9 (−1.9, −1.8)
ANBP	1721 / 1706	157 (15)	−4.0 (−4.1, −3.9)	100 (10)	−1.9 (−2.0, −1.8)
BENEDICT	904 / 300	151 (14)	−4.0 (−4.1, −3.9)	88 (10)	−1.9 (−2.0, −1.8)
CAMELOT	1336 / 655	129 (16)	−4.0 (−4.1, −3.9)	78 (10)	−1.9 (−1.9, −1.8)
DIABHYCAR	2443 / 2469	145 (15)	−4.1 (−4.2, −4.0)	82 (10)	−2.0 (−2.0, −1.9)
Dutch TIA	732 / 741	157 (25)	−4.0 (−4.1, −3.9)	91 (10)	−1.9 (−2.0, −1.8)
EUROPA	6110 / 6108	137 (16)	−4.0 (−4.1, −3.9)	82 (10)	−1.9 (−2.0, −1.8)
EWPHE	416 / 424	183 (17)	−3.9 (−4.0, −3.8)	101 (10)	−1.9 (−1.9, −1.8)
HOPE	4645 / 4652	139 (20)	−4.1 (−4.2, −4.0)	79 (10)	−2.0 (−2.0, −1.9)
HYVET	1933 / 1912	173 (9)	−3.9 (−4.0, −3.8)	91 (10)	−1.9 (−1.9, −1.8)
IDNT	1146 / 569	159 (20)	−4.0 (−4.1, −3.9)	87 (11)	−1.9 (−2.0, −1.8)
PART–2	308 / 309	133 (17)	−4.0 (−4.1, −3.9)	79 (10)	−1.9 (−2.0, −1.8)
PEACE	4158 / 4132	133 (17)	−4.1 (−4.2, −4.0)	77 (10)	−1.9 (−2.0, −1.9)
PREVEND IT	431 / 433	130 (18)	−4.0 (−4.1, −3.9)	76 (10)	−1.9 (−2.0, −1.8)
PREVENT	417 / 408	129 (17)	−4.0 (−4.1, −3.9)	79 (10)	−1.9 (−2.0, −1.8)
PROFESS	9873 / 9925	144 (17)	−4.1 (−4.3, −4.0)	84 (11)	−1.9 (−2.0, −1.9)
PROGRESS	3051 / 3054	147 (19)	−3.9 (−4.1, −3.8)	86 (10)	−1.9 (−1.9, −1.8)
SHEP	2365 / 2371	170 (9)	−3.9 (−4.0, −3.8)	77 (10)	−1.9 (−1.9, −1.8)
Syst–Eur	2398 / 2297	174 (10)	−4.0 (−4.1, −3.8)	86 (10)	−1.9 (−2.0, −1.8)
TRANSCEND	2954 / 2972	141 (17)	−4.0 (−4.1, −3.9)	82 (10)	−1.9 (−1.9, −1.8)
All placebo–controlled trials	57,430 / 55,504	145 (21)	−4.0 (−4.1, −3.9)	83 (11)	−1.9 (−2.0, −1.8)
BP–lowering intensity trials					
AASK	540 / 554	150 (24)	−8.0 (−8.1, −7.8)	95 (10)	−3.7 (−3.8, −3.5)
ABCD	474 / 476	155 (17)	−8.4 (−8.6, −8.2)	98 (10)	−3.9 (−4.0, −3.8)
ACCORD	2362 / 2371	139 (15)	−7.5 (−7.7, −7.3)	76 (10)	−3.7 (−3.9, −3.6)
Cardio–Sis	558 / 553	158 (9)	−8.5 (−8.7, −8.3)	87 (10)	−4.0 (−4.2, −3.9)
HDFP	5553 / 5387	159 (23)	−8.2 (−8.4, −8.0)	101 (9)	−3.7 (−3.9, −3.6)
HOMED–BP	1759 / 1759	154 (18)	−9.2 (−9.4, −9.1)	90 (10)	−4.4 (−4.5, −4.3)
SPRINT	4678 / 4683	140 (16)	−6.5 (−6.8, −6.2)	78 (10)	−3.1 (−3.3, −3.0)
UKPDS	758 / 390	159 (17)	−8.3 (−8.5, −8.1)	93 (10)	−3.9 (−4.1, −3.8)
VALISH	1545 / 1534	169 (9)	−9.0 (−9.2, −8.8)	82 (10)	−4.4 (−4.5, −4.2)
All BP–lowering intensity trials	12,674 / 12,320	148 (19)	−7.6 (−7.7, −7.4)	82 (12)	−3.7 (−3.8, −3.6)
Drug class comparison trials					
AASK	653 / 441	150 (24)	−1.4 (−1.5, −1.3)	95 (10)	−0.5 (−0.6, −0.5)
ABCD	235 / 235	155 (17)	−1.4 (−1.5, −1.3)	98 (10)	−0.5 (−0.6, −0.5)
ALLHAT	15,255 / 27,163	146 (16)	−1.4 (−1.5, −1.3)	84 (10)	−0.6 (−0.7, −0.6)
ANBP2	3039 / 3044	168 (13)	−1.5 (−1.5, −1.4)	91 (10)	−0.6 (−0.6, −0.5)
ASCOT–BPLA	9639 / 9618	164 (18)	−1.1 (−1.2, −1.0)	95 (10)	−0.4 (−0.5, −0.4)
BENEDICT	602 / 303	151 (14)	−1.4 (−1.5, −1.3)	88 (10)	−0.5 (−0.6, −0.5)
CAMELOT	663 / 673	129 (16)	−1.4 (−1.5, −1.3)	78 (10)	−0.5 (−0.6, −0.5)
CAPPP	5493 / 5492	161 (20)	−1.4 (−1.4, −1.3)	99 (10)	−0.6 (−0.6, −0.5)
CASE–J	2349 / 2354	163 (14)	−1.4 (−1.5, −1.3)	92 (10)	−0.6 (−0.7, −0.5)
COLM	2568 / 2573	158 (13)	−1.4 (−1.5, −1.3)	87 (10)	−0.6 (−0.7, −0.6)
CONVINCE	8179 / 8297	150 (16)	−1.5 (−1.6, −1.4)	87 (10)	−0.6 (−0.7, −0.5)
COPE	2183 / 1110	154 (12)	−1.4 (−1.5, −1.3)	89 (10)	−0.6 (−0.7, −0.6)
E–COST	1053 / 995	164 (18)	−1.4 (−1.5, −1.3)	94 (10)	−0.6 (−0.7, −0.6)
ELSA	1177 / 1157	160 (15)	−1.4 (−1.5, −1.3)	98 (10)	−0.6 (−0.7, −0.6)
HIJ–CREATE	1024 / 1025	135 (18)	−1.4 (−1.5, −1.3)	76 (10)	−0.6 (−0.7, −0.6)
HOMED–BP	2347 / 1171	154 (18)	−1.4 (−1.5, −1.3)	90 (10)	−0.6 (−0.7, −0.6)
IDNT	579 / 567	160 (20)	−1.4 (−1.5, −1.3)	87 (11)	−0.6 (−0.7, −0.6)
INSIGHT	3164 / 3157	173 (15)	−1.4 (−1.5, −1.4)	99 (10)	−0.6 (−0.7, −0.5)
INVEST	10,648 / 10,672	151 (20)	−1.5 (−1.6, −1.4)	87 (10)	−0.6 (−0.7, −0.6)
JMIC–B	828 / 822	146 (19)	−1.4 (−1.5, −1.3)	82 (10)	−0.6 (−0.7, −0.5)
LIFE	4605 / 4588	174 (14)	−1.4 (−1.5, −1.3)	98 (10)	−0.7 (−0.7, −0.6)
MOSES	671 / 681	151 (18)	−1.4 (−1.5, −1.3)	87 (10)	−0.6 (−0.7, −0.6)
NICS–EH	204 / 210	172 (12)	−1.4 (−1.5, −1.3)	94 (10)	−0.6 (−0.7, −0.6)
NORDIL	5471 / 5410	173 (18)	−1.3 (−1.4, −1.2)	106 (10)	−0.6 (−0.7, −0.6)
ONTARGET	8502 / 17,118	142 (17)	−1.3 (−1.4, −1.2)	82 (10)	−0.5 (−0.6, −0.5)
STOP HPN–2	2213 / 4401	194 (15)	−1.4 (−1.4, −1.3)	98 (10)	−0.6 (−0.7, −0.6)
UKPDS	400 / 358	159 (17)	−1.4 (−1.5, −1.3)	93 (10)	−0.6 (−0.7, −0.6)
VALUE	7596 / 7649	154 (19)	−1.3 (−1.4, −1.2)	87 (10)	−0.5 (−0.6, −0.5)
VHAS	707 / 707	169 (10)	−1.4 (−1.5, −1.3)	102 (10)	−0.6 (−0.7, −0.6)
All drug class comparison trials	100,972 / 123,066	156 (21)	−1.4 (−1.5, −1.3)	89 (12)	−0.5 (−0.6, −0.5)

−10.0 −8.0 −6.0 −4.0 −2.0 0.0

Systolic blood pressure difference (mmHg)

−5.0 −4.0 −3.0 −2.0 −1.0 0.0

Diastolic blood pressure difference (mmHg)

Canoy D, *et al. Heart* 2022; 108:1281–1289. doi: 10.1136/heartjnl-2021-320171

Table S1. **List of trials and interventions assigned to the active and control arms in the analyses**.

A. Blood pressure difference trials (9 trials)

Trial	Active group	Control group
**Trials comparing blood pressure-lowering targets (8 trials)**		
AASK	More intense treatment	Less intense treatment
ABCD	More intense treatment	Less intense treatment
ACCORD	More intense treatment	Less intense treatment
CARDIO-SIS	More intense treatment	Less intense treatment
HDFP	More intense treatment	Less intense treatment
HOMED-BP	More intense treatment	Less intense treatment
SPRINT	More intense treatment	Less intense treatment
UKPDS	More intense treatment	Less intense treatment
VALISH	More intense treatment	Less intense treatment
**Blood pressure-lowering trials comparing treatment versus placebo (21 trials)**		
ACTIVE-I	ARB	Placebo
ADVANCE	ACEI and diuretic	Placebo
ANBP	Diuretic	Placebo
BENEDICT	ACEI, CCB and ACEI/CCB	Placebo
CAMELOT	CCB and ACEI	Placebo
DIABHYCAR	ACEI	Placebo
DUTCH-TIA	ß-blocker	Placebo
EUROPA	ACEI	Placebo
EWPHE	Diuretic	Placebo
HOPE	ACEI	Placebo
HYVET	Diuretic	Placebo
IDNT	ARB and CCB	Placebo
PART 2	ACEI	Placebo
PEACE	ACEI	Placebo
PREVEND IT	ACEI	Placebo
PREVENT	CCB	Placebo
PROFESS	ARB	Placebo
PROGRESS	ACEI and/or diuretic	Placebo
SHEP	ß-blocker and diuretic	Placebo
SYST-EUR	CCB	Placebo
TRANSCEND	ARB	Placebo

Canoy D, *et al. Heart* 2022; 108:1281–1289. doi: 10.1136/heartjnl-2021-320171

Table S1. List of trials and interventions assigned to the active and control arms in the analyses (cont'd).

B. Drug comparison trials (29 trials)

Trial	Active group	Control group
AASK	ACEI and CCB	ß-blocker
ABCD	CCB	ACEI
ALLHAT	Diuretic	ACEI, CCB and α-blocker
ANBP2	Diuretic	ACEI
ASCOT-BPLA	CCB-based	ß-blocker-based
BENEDICT	ACEI and ACEI/CCB	CCB
CAMELOT	CCB	ACEI
CAPPP	ß-blocker and/or diuretic	ACEI
CASE-J	CCB	ARB
COLM	ARB and diuretic	ARB and CCB
CONVINCE	CCB	ß-blocker or diuretic
COPE	CCB/diuretic and CCB/ ß-blocker	CCB and ARB
E-COST	ARB	Conventional
ELSA	CCB	ß-blocker
HIJ-CREATE	ARB	non-ARB
HOMED-BP	CCB	ACEI and ARB
IDNT	ARB	CCB
INSIGHT	Diuretic	CCB
INVEST	CCB	non-CCB
JMIC-B	CCB	ACEI
LIFE	ARB	ß-blocker
MOSES	CCB	ARB
NICS-EH	Diuretic	CCB
NORDIL	ß-blocker and/or diuretic	CCB
ONTARGET	ARB/ACEI	ACEI and ARB
STOP HYPERTENSION-2	ß-blocker and/or diuretic	ACEI and CCB
UKPDS	ß-blocker	ACEI
VALUE	CCB-based	ARB-based
VHAS	Diuretic	CCB

Acronyms are described in full in the Trial acronym legend in the Supplement; CCB – calcium channel-blocker; ACEI – angiotensin-converting enzyme inhibitor; ARB – angiotensin II receptor blocker.

Table S2. Mean baseline blood pressure according to baseline characteristics and trial designs.

A. Systolic blood pressure (mmHg)

	**Blood pressure-lowering intensity trials**	**Placebo-controlled trials**	**All blood pressure difference trials**	**Drug class comparison trials**
	Mean (SD)	Mean (SD)	Mean (SD)	Mean (SD)
**ALL PARTICIPANTS**	**151 (21)**	**146 (20)**	**147 (21)**	**156 (21)**
By baseline characteristics				
1. Systolic blood pressure (mmHg)				
<120	113 (6)	112 (6)	112 (6)	112 (6)
120 to 129	125 (3)	124 (3)	124 (3)	124 (3)
130 to 139	135 (3)	133 (3)	134 (3)	134 (3)
140 to 149	144 (3)	143 (3)	143 (3)	144 (3)
150 to 159	154 (3)	153 (3)	153 (3)	154 (3)
≥160	174 (14)	171 (11)	172 (12)	175 (14)
2. Age (years)				
<50	150 (20)	138 (18)	144 (20)	155 (19)
50 to 65	151 (21)	142 (19)	144 (20)	154 (20)
65 to 79	151 (21)	147 (20)	148 (21)	157 (21)
≥80	154 (19)	160 (21)	159 (21)	160 (23)
3. Sex				
Men	149 (20)	143 (19)	144 (20)	154 (20)
Women	154 (22)	151 (21)	152 (22)	158 (21)
4. Body mass index (kg/m^2)				
<25	155 (19)	147 (21)	148 (21)	158 (22)
25 to 30	147 (18)	146 (20)	146 (20)	155 (21)
≥30	142 (18)	146 (20)	145 (19)	157 (21)
5. Cardiovascular disease history				
No	148 (19)	154 (21)	153 (21)	161 (20)
Yes	144 (19)	141 (19)	141 (19)	150 (20)
6. Diabetes history				
No	152 (21)	147 (22)	149 (21)	157 (21)
Yes	147 (20)	146 (19)	145 (20)	153 (19)
7. Antihypertensive drug use				
No	150 (21)	149 (21)	149 (21)	163 (19)
Yes	152 (21)	144 (20)	145 (20)	151 (20)

Canoy D, *et al. Heart* 2022; 108:1281–1289. doi: 10.1136/heartjnl-2021-320171

Table S2. Mean baseline blood pressure according to baseline characteristics and trial designs (cont'd).

B. Diastolic blood pressure (mmHg)

	**Blood pressure-lowering intensity trials**	**Placebo-controlled trials**	**All blood pressure difference trials**	**Drug class comparison trials**
	Mean (SD)	Mean (SD)	Mean (SD)	Mean (SD)
**ALL PARTICIPANTS**	**88 (15)**	**83 (11)**	**84 (12)**	**90 (12)**
By baseline characteristics				
1. Diastolic blood pressure (mmHg)				
<70	63 (5)	63 (5)	63 (5)	64 (5)
70 to 79	75 (3)	74 (3)	74 (3)	75 (3)
80 to 89	84 (3)	83 (3)	83 (3)	84 (3)
90 to 99	94 (3)	93 (3)	93 (3)	94 (3)
100 to 109	104 (3)	102 (3)	103 (3)	103 (3)
≥110	118 (8)	115 (7)	117 (7)	113 (5)
2. Age (years)				
<50	100 (11)	87 (12)	94 (13)	99 (11)
50 to 65	90 (13)	84 (11)	86 (12)	92 (12)
65 to 79	80 (13)	81 (10)	81 (11)	87 (12)
≥80	74 (11)	84 (11)	82 (12)	84 (12)
3. Sex				
Men	88 (15)	83 (11)	84 (12)	90 (12)
Women	88 (14)	83 (11)	85 (12)	90 (12)
4. Body mass index (kg/m^2)				
<25	83 (12)	82 (11)	82 (11)	89 (12)
25 to 30	82 (13)	83 (10)	83 (11)	90 (12)
≥30	82 (13)	84 (11)	83 (11)	90 (12)
5. Cardiovascular disease history				
No	83 (12)	84 (11)	86 (13)	93 (12)
Yes	79 (14)	82 (10)	82 (11)	86 (12)
6. Diabetes history				
No	89 (15)	84 (11)	85 (13)	91 (12)
Yes	83 (13)	82 (10)	82 (11)	86 (11)
7. Antihypertensive drug use				
No	89 (15)	84 (11)	86 (13)	95 (11)
Yes	86 (15)	82 (11)	83 (11)	87 (12)

Canoy D, *et al. Heart* 2022; 108:1281–1289. doi: 10.1136/heartjnl-2021-320171

Table S3. Characteristics of trials included in the BPLTTC study.

A. Trials comparing different blood pressure-lowering targets intensity

Trial	Setting	Inclusion criteria	Exclusion criteria	Recruitment period	Randomisation groups, No. of participants (% women)			Treatment goals for blood pressure control		Treatment	Follow-up duration (y)	No. of follow-up BP measures
					All	More intense	Less intense	More intense	Less intense			
AASK[12 13]	USA	Age 18-70 years, African-American, hypertension, renal disease (GFR=20-65 ml/min per 1.73m^2)	DBP <95 mmHg, diabetes, urine protein:creatinine ratio >25, recent malignant hypertension, secondary hypertension, non-blood pressure-related CKD, serious systemic disease, heart failure	Feb 1995 to Sept 1998	1094 (39)	540 (38)	554 (40)	Mean arterial pressure ≤92 mmHg	Mean arterial pressure 102-107 mmHg	1 of 3: β-blocker (Metoprolol), ACEI (Ramipril), CCB (Amlodipine); Plus furosemide, doxazosin, clonidine, and hydralazine or minoxidil sequentially	4.8	54
ABCD[14-16]	USA	Age 0-74 years, with T2D, DBP ≥80 mmHg, not on antihypertensive treatment	Recent CAD or CeVD, heart failure, renal disease	Mar 1991 to May 1993	950 (39)	474 (40)	476 (38)	DBP <75 mmHg for hypertensives (DBP ≥90 mmHg) or DBP reduction by 10 mmHg for normotensives	DBP 80-89 mmHg for hypertensives (DBP ≥90 mmHg) or no change for normotensives	Hypertensive group: CCB (Nisoldipine) and ACEI (Enalapril); plus β-blocker (Metoprolol), diuretic (HCTZ), or others but not CCB or ACEI; Normotensive group: CCB (Nisoldipine), ACEI (Enalapril) or placebo	4.7	8
ACCORD[17]	USA and Canada	Age ≥40y years with CVD or ≥50 years with substantial atherosclerosis, T2D, HbA1c ≥7.5%, albuminuria, LVH or ≥2 CVD risk factors (dyslipidaemia, hypertension, smoking, obesity); SBP 130-180 mmHg and taking ≤3 antihypertensive drugs, 24-hour protein excretion rate <1g	Body mass index ≥45 kg/m^2, serum creatinine ≥132.6 μmol/l and other serious illness	Jan to Jun 2001, then Jan 2003 to Oct 2005	4733 (48)	2362 (48)	2371 (48)	SBP <120 mmHg	SBP <140 mmHg	Drug classes available in clinical practice	4.7	20
Cardio-Sis[2 18]	Italy	Age ≥55 years, SBP ≥150 mmHg, taking antihypertensive drug ≥12 weeks, ≥1 CV risk factor (smoking, dyslipidaemia, family history of premature CVD, prior TIA or stroke, established CAD or PAD	Fasting blood glucose ≥7 mmol/l, diabetes, serious conditions, renal disease, valvular heart disease, left ventricular hypertrophy, atrial fibrillation, substance misuse.	Feb 2005 to Feb 2007	1111 (59)	558 (59)	553 (59)	SBP <130 mmHg	SBP <140 mmHg	Diuretic (Furosemide), ACEI (Ramipril), ARB (Telmisartan), CCB (Amlodipine), β -blocker (Bisoprolol), Clonidine	4.7	No data
HDFP[19]	USA	Age 30-69 years, hypertensive with DBP ≥ 90 mmHg	None specified	Feb 1973 to Feb 1974	10,940 (46)	5485 (46)	5455 (46)	≤90 mmHg DBP if baseline DBP ≥100 mmHg or receiving antihypertensive therapy at baseline, or a reduction of 10 mmHg in DBP if baseline DBP 90-90 mmHg	Referred to community physicians for care	Diuretic (Chlorthalidone; plus triamterene or spironolactone if necessary); plus antiadrenergic drug (reserpine, alternatively, methyldopa); plus vasodilator (Hydralazine); plus antiadrenergic drug (Guanethidine); then addition or substitution of other drugs	6.7	5 (all); More intense group=24 (median)
HOMED-BP[20]	Japan	Self-measured SBP 135-179 mmHg or DBP 85-119 mmHg, but not if DBP <65 or SBP <110 mmHg (clinic SBP <220 mmHg and DBP <125 mmHg)	None specified	May 2001 to Oct 2009	3518 (50)	1759 (50)	1759 (50)	SBP <120 and DBP <80 mmHg	BP 125-134/80-84 mmHg	ACEI, ARB or CCB; Diuretic; β-blocker; then other drug class (avoid reaching BP <110/65 mmHg)	4.9	8

SPRINT[21]	USA and Puerto Rico	Age ≥50y years, SBP 130-180 mmHg, increased CVD risk (clinical/subclinical CVD other than stroke, CKD excluding polycystic kidney disease and with eGFR of 20-60 ml/min/1.73m² body surface area, 10-year Framingham CVD risk ≥15%, age ≥75y)	Diabetes or prior stroke	Nov 2010 to Mar 2013	9361 (36)	4678 (36)	4683 (35)	SBP <120 mmHg	SBP <140 mmHg	All major drug classes	3.0	13
UKPDS[22-24]	UK	Age 25-65 years, newly-diagnosed diabetes, and hypertension (untreated: SBP ≥160 mmHg and/or DBP ≥90 mmHg; treated: SBP ≥150 mmHg and/or DBP ≥85 mmHg)	Ketonuria, recent MI, angina, heart failure, >1 major vascular episode, serum creatinine >15 μmol/, retinopathy, malignant hypertension, uncorrected endocrine abnormality, severe concurrent illness	1987 to 1991	1148 (45)	758 (46)	390 (42)	BP <150/85 mmHg	BP <180/105 mmHg	ACEI (Captopril) and β-blocker (Atenolol) for more intense arm; For both arms: diuretic (Furosemide), CCB (Nifedipine), methlydopa then α-blocker (Prazosin)	7.9	5
VALISH[25 26]	Japan	Age ≥70 to <85 years, isolated hypertension (SBP >160 mmHg and DBP <90 mmHg)	Secondary or malignant hypertension, BP ≥200/≥90 mmHg, recent CeVD or MI, recent/planned revascularisation, heart failure, aortic stenosis, valvular heart disease, atrial fibrillation/flutter, serious arrhythmia, renal/liver dysfunction	Feb 2004 to Aug 2005	3079 (62)	1545 (62)	1534 (63)	SBP <140 mmHg	SBP ≥140 to <150 mmHg	ARB (Valsartan) then other antihypertensive agents such as diuretics and CCB but not other ARB drugs	2.6	11

Table S3. Characteristics of trials included in the BPLTTC study (cont'd).

B. Blood pressure-lowering trials comparing treatment with placebo

Trial	Setting	Inclusion criteria	Exclusion criteria	Recruitment period	Randomisation group, No. of participants (% of women)			Drug intervention	Any additional treatment	Treatment goal for blood pressure control	Follow-up duration (years)	No. of follow-up BP measures
					All	Inter-vention	Placebo					
ACTIVE I[27]	Multi-country	Atrial fibrillation, ≥1 risk factor (age ≥75 years, on antihypertensive treatment, history of stroke, TIA or non-CNS embolism, LVEF <45%, PVD, or age 55-74 years with either CAD or diabetes)	Use of anticoagulant, peptic ulcer disease in past 6 months, history of intracerebral haemorrhage, thrombocytopaenia or mitral stenosis	Jun 2003 to May 2006	9016 (39)	4518 (39)	4498 (39)	ARB (Irbesartan)	None	None specified	4.1	9
ADVANCE[28]	Multi-country	Age ≥55 years T2D (diagnosed aged ≥30y), ≥1 major CVD or ≥1 CVD risk factor (microvascular disease, smoking, dyslipidaemia, microalbuminuria, T2D for ≥10 years, age ≥65 years)	HbA1c target (≤6.5%), definite indication for long-term insulin therapy	Jul 2001 to Mar 2003	11,140 (43)	5569 (42)	5571 (43)	ACEI (Perindopril) and diuretics (Indapamide) as fixed dose combination drug	At physician's discretion, but not thiazide diuretics, and only perindopril as ACEI allowed	None specified	4.2	12
ANBP[29 30]	Australia	Age 30-69 years with mild hypertension (DBP 95-110 mmHg and SBP <200 mmHg)	Antihypertensive treatment in past 3 months, recent angina or MI, stroke, hormone therapy, asthma, diabetes, gout, serious disease, tricyclic antidepressant use	1973 to March 1979	3427 (37)	1721 (37)	1706 (36)	Diuretic (Chlorothiazide)	Methyldopa, propranolol, or pindolol, then hydralazine or clonidine	Reduce DBP to ≤90 mmHg (after two years, further reduced to 80 mmHg)	3.6	4
BENEDICT[31 32]	Italy	Age ≥40 years, untreated SBP ≥130 / DBP ≥85 mmHg or needing treatment to attain below these levels, T2D for <25 years, urinary albumin excretion rate <20 μg/min, serum creatinine ≤133 μmol/l	HbA1c ≥11%, nondiabetic renal disease	Around 2000 to 2003	1209 (48)	907 (47)	302 (50)	ACEI (Trandolapril), CCB (Verapamil), both ACEI and CCB	Diuretics (HCTZ or furosemide), doxazosin, prazosin, clonidine, methyldopa or β-blocker, minoxidil, or CCB	Reduce BP to 120/80 mmHg	3.1	14
CAMELOT[33]	Multi-country (US, Canada, Europe)	Age 30-79 years, coronary artery stenosis >20% by angiography, DBP <100 mmHg	Left middle coronary artery obstruction >50%, LVEF <40%, heart failure	Apr 1999 to Apr 2002	1991 (26)	1336 (26)	655 (27)	CCB (Amlodipine), ACEI (Enalapril)	Allowed to continue β-blocker, α-blocker, diuretics	None specified	1.6	7
DIABHYCAR[34 35]	Multi-country	Age ≥50 years, T2D, urinary albumin excretion ≥20 mg/l in two consecutive urine samples	Serum creatinine >150 μmol/l, use of insulin, ACEI or ARB, heart failure, recent MI, urinary tract infection	Feb 1995 to Apr 1998	4912 (30)	2443 (30)	2469 (30)	ACEI (Ramipril)	Usual treatment	None specified	3.9	7
Dutch TIA Trial[36]	The Netherlands	TIA or non-disabling ischaemic stroke (Rankin Scale ≤3) in past 3 months	Cerebral ischaemia from identifiable causes other than arterial thrombosis or embolism	Feb 1986 to Mar 1989	1473 (36)	732 (34)	741 (38)	β-blocker (Atenolol)	None specified	None specified	2.3	6
EUROPA[37 38]	Multi-country (Europe)	Age ≥18 years, documented MI >3 months before screening, revascularisation >6 months before screening, >70% coronary obstruction	Heart failure, hypotension, uncontrolled hypertension, renal insufficiency, serum potassium >5.5 mmol/L	Oct 1997 to Jun 2000	12,218 (15)	6110 (14)	6108 (15)	ACEI (Perindopril)	None specified	None specified	4.2	9
EWPHE[39 40]	Multi-country	Age ≥60 years, BP 160-239/90-119 mmHg	Curable causes of high BP, retinopathy, heart failure, stroke history, hepatitis/cirrhosis, gout, malignancy, diabetes requiring insulin treatment	From 1972	840 (70)	416 (69)	424 (71)	Diuretic (HCTZ or triamterene)	Methyldopa	Reduce BP but at unspecified levels	4.6	11
HOPE[41]	Multi-country	Age ≥55 years, CAD, stroke, PVD or diabetes, plus ≥1 risk factor (hypertension, dyslipidaemia,	Heart failure, left ejection fraction <40%, using ACEI or Vitamin E, uncontrolled	Dec 1993 to Jun 1995	9297 (27)	4656 (28)	4652 (26)	ACEI (Ramipril)	None specified	None specified	4.5	3

		smoking, or documented microalbuminuria)	hypertension, nephropathy, or recent MI or stroke									
HYVET[42]	Multi-country	Age ≥80y years, sustained SBP ≥160 mmHg	Accelerated or secondary hypertension, recent haemorrhagic stroke, heart failure, serum creatinine >150 μmol/L, serum potassium <3.5 or >5.5 mmol/L, gout, and dementia	From 2000	3845 (60)	1933 (61)	1912 (60)	Diuretic (Indapamide)	ACEI (Perindopril)	Reduce BP to 150/80 mmHg	2.1	5
IDNT[1]	USA	Age 30-70 years, T2D, hypertension (BP ≥135/85 mmHg or taking anti-hypertensive drug), proteinuria, serum creatinine (μmol/l): 88 to 265 (women) or 106 to 265 (men)	None specified	Mar 1996 to Feb 1999	1715 (34)	1146 (36)	569 (29)	ARB (Irbesartan) and CCB (Amlodipine)	Others except ACEI, ARB and CCB	SBP <135 (10 mmHg lower if baseline value >145 mm Hg); DBP <85 mmHg	2.6	No data
PART 2[43]	New Zealand	Age ≤75 years, diagnosis (in past 5 year) of MI, documented CAD, TIA or intermittent claudication	Heart failure, serious nonvascular disease, SBP >160 mmHg, DBP >100 mm Hg, DBP <100 mmHg during pre-randomisation run-in period	Not specified; Publication in 2000	617 (18)	308 (18)	309 (18)	ACEI (Ramipril)	None	None specified	4.6	5
PEACE[44]	Multi-country (USA, Puerto Rico, Canada and Italy)	Age ≥50 years, documented CAD	Unstable angina, severe valvular heart disease, recent revascularisation, planned elective revascularisation, limited 5-year survival, serum creatinine >177 μmol/l, serum potassium >5.5 mmol/l	Nov 1996 to Jun 2000	8290 (18)	4158 (19)	4132 (17)	ACEI (Trandolapril)	None	None specified	4.7	7
PREVEND IT[45]	The Netherlands	Microalbuminuria, SBP <160/100 mmHg (no previous antihypertension treatment)	Creatinine clearance <60% of normal age-adjusted value	Apr 1998 to Jun 1999	864 (35)	431 (34)	433 (36)	ACEI (Fosinopril)	None	None specified	3.8	16
PREVENT[46 47]	USA and Canada	Age 30-80 years, documented CAD, DBP <95 mmHg, cholesterol <325 mg/dl, fasting blood glucose <200 mg/dl	Contraindication for dihydropyridines, uncontrolled hypertension, diabetes and other major illness	Nov 1992 to Sep 1994	825 (20)	417 (20)	408 (20)	CCB (Amlodipine)	None	None specified	3.0	3
PRoFESS[48 49]	Multi-country	Age ≥55 years with ischaemic stroke <90 days before randomisation (later modified to include age 50 to 54 years or had stroke 90 to 120 days before randomisation if with ≥2 additional risk factors: diabetes, hypertension, smoker, obesity previous CVD, end-organ damage or hyperlipidaemia) and remained stable[a]	Haemorrhagic stroke, severe disability after the qualifying stroke, contraindication to treatments	Sep 2003 to Jul 2006	19,798 (36)	9873 (35)	9925 (36)	ARB (Telmisartan)	At physician's discretion to control blood pressure: diuretic, then β-blocker or CCB, then ACEI but not ARB.	None specified	2.5	6
PROGRESS[50 51]	Multi-country (Asia, Australasia and Europe)	Stroke or TIA in past 5 years	Indication or contraindication for ACEI	Jun 1995 to Nov 1997	6105 (30)	3051 (30)	3054 (30)	ACEI (Perindopril) and/or diuretic (Indapamide)	None	Intensity of lowering based on clinical intention determined before randomisation	3.9	4
SHEP[52]	USA	Age ≥60 years, isolated systolic hypertension (BP 160-219/<90 mmHg, not on treatment)	Major CVD, cancer, alcoholic liver disease, renal dysfunction, competing risk of SHEP primary endpoint or presence of medical management exclusions	Mar 1985 to Jan 1988	4736 (57)	2365 (56)	2371 (57)	Diuretic (Chlorthalidone) and β-blocker (Atenolol)	None	If baseline SBP >180 mmHg: reduce to <160 mmHg; If baseline SBP 160-179 mmHg, reduce by 20 mmHg	5.0	4

SYST-EUR[53]	Multi-country	Age ≥60 years, sitting SBP 160-219 mmHg, sitting DBP <95 mmHg, and standing SBP ≥140 mmHg	Secondary hypertension, retinal haemorrhage/papilloedema, heart failure, dissecting aortic aneurysm, serum creatinine ≥180 μmol/l, recent severe nosebleeds, stroke or MI, dementia, disorders prohibiting standing position, severe CVD/non-CVD	Dec 1988 to Jan 1997	4695 (67)	2398 (67)	2297 (66)	CCB (Nitrendipine)	Combined with or replaced by ACEI (Enalapril), diuretic (HCTZ), or both	Reduce sitting SBP by ≥20 mmHg to <150 mmHg	2.6	3
TRANSCEND[54 55]	Multi-country	Intolerant to ACEI and with established CAD, PVD, CeVD or diabetes with end-organ damage	Heart failure, valvular/cardiac outflow tract obstruction, pericarditis, congenital heart disease, unexplained syncope, recent revascularisation, SBP >160 mmHg, heart transplantation, subarachnoid haemorrhage, significant renal stenosis, renal or hepatic dysfunction	Nov 2001 to May 2004	5926 (43)	2954 (43)	2972 (43)	ARB (Telmisartan)	None	None specified	4.9	9

Table S3. Characteristics of trials included in the BPLTTC study (cont'd).

C. Trials comparing different drug classes

Trial	Country	Inclusion criteria	Exclusion criteria	Recruitment period	Randomisation groups	No. of participants (% women)	Additional (open-label) treatment	Treatment target for blood pressure control	Follow-up duration (y)	No. of follow-up BP measures
AASK[12 13]	USA	Age 18-70 years, African-American, hypertension, renal disease (GFR=20-65 ml/min per 1.73m²)	DBP <95 mmHg, diabetes, urine protein:creatinine ratio >25, recent malignant or hypertension, non-blood pressure-related CKD, serious systemic disease, heart failure	Feb 1995 to Sept 1998	All<br>ACEI (Ramipril)<br>CCB (Amlodipine)<br>β-blocker (Metoprolol)	1094 (39)<br>436 (39)<br>217 (39)<br>441 (39)	Furosemide, doxazosin, clonidine, hydralazine and minoxidil (sequentially)	None specified	4.8	54
ABCD[14-16]	USA	Age 0-74 years, with T2D, DBP ≥80 mmHg, not on antihypertensive treatment	Recent CAD or CeVD, heart failure, renal disease	Mar 1991 to May 1993	All<br>CCB (Nisoldipine)<br>ACEI (Enalapril)	470 (33)<br>235 (32)<br>235 (33)	β-blocker (Metoprolol), diuretic (HCTZ), or others but not CCB or ACEI	None specified	4.7	8
ALLHAT[56 57]	Multi-country	Age ≥55y years stage 1 or 2 hypertension plus ≥1 risk factor (MI or stroke >6 months previously, left ventricular hypertrophy, T2D, smoking, HDL <0.91 mmol/l), other atherosclerotic CVD	Symptomatic or hospitalisation for heart failure, LVEF <35%	Feb 1994 to Jan 1998	All<br>Diuretic (Chlorthalidone)<br>CCB (Amlodipine)<br>ACEI (Lisinopril)<br>α-blocker (Doxazosin)	42,418 (47)<br>15,255 (47)<br>9048 (47)<br>9054 (46)<br>9061 (46)	Atenolol, clonidine or reserpine	BP <140/90 mmHg	4.8	3
ANBP2[58]	Australia	Age 65-84 years, SBP ≥160 mmHg or DBP ≥90 mmHg (if SBP≥140 mmHg), no recent CVD	Serious illness, plasma creatinine >221 μmol/l, malignant hypertension, dementia	April 1995 to Jun 1998	All<br>ACEI (Enalapril)<br>Diuretic (HCTZ)	6083 (51)<br>3044 (50)<br>3039 (52)	β-blocker, CCB and α-blocker	Lower SBP by 20 mmHg to <160 mmHg (<140 mmHg if tolerated,) and by 10 mmHg DBP to <90 mmHg (<80 mmHg if tolerated)	4.1	9
ASCOT-BPLA[59 60]	Multi-country	Age 40-79 years, untreated (SBP ≥160 or DBP ≥100 mmHg) or treated hypertension (SBP ≥140 or DBP ≥90 mmHg), ≥3 CVD risk factors (documented LVH, abnormal ECG, T2D, PAD, previous stroke or TIA, male sex, age ≥55 years, microalbuminuria or proteinuria, smoking, TC:HDL ≥6, family history of premature coronary heart disease	Previous MI, current treatment for angina, recent CeVD, fasting triglycerides >4.5 mmol/l, heart failure, arrhythmia, haematological or biochemical abnormality at screening	Feb 1998 to May 2000	All<br>CCB (Amlodipine-based)<br>β-blocker (Atenolol-based)	19,257 (23)<br>9639 (23)<br>9618 (23)	For CCB arm: plus ACEI (Perindopril); For β-blocker arm: plus diuretic (bendroflumethiazide) and potassium	With diabetes: BP <140/90 mmHg; Without diabetes: BP <130/80 mmHg	5.3	12
BENEDICT[31 32]	Italy	Age ≥40 years, untreated SBP ≥130 or DBP ≥85 mmHg or needing treatment to attain below these levels, T2D for <25 years, urinary albumin excretion rate <0 μg/min on two consecutive measures, serum creatinine ≤133 μmol/l	HbA1c ≥11%, nondiabetic renal disease	Around 2000 to 2003	All (excluding placebo)<br>ACEI (Trandolapril)<br>CCB (Verapamil)<br>ACEI (Trandolapril) and CCB (Verapamil)	907 (47)<br>302 (48)<br>303 (46)<br>302 (45)	Diuretic (HCTZ or furosemide), then doxazosin, prazosin, clonidine, methyldopa or β-blocker, then minoxidil, or CCB	BP 120/80 mmHg	3.1	14
CAMELOT[33]	Multi-country	Age 30-79 years, coronary artery stenosis >20% by angiography and DBP <100 mmHg	Left middle coronary artery obstruction >50%, LVEF <40%, heart failure	Apr 1999 to Apr 2002	All (excluding placebo)<br>CCB (Amlodipine)<br>ACEI (Enalapril)	1336 (26)<br>663 (24)<br>673 (28)	Allowed to continue β-blocker, α-blocker, diuretic	None specified	1.6	7
CAPPP[61 62]	Sweden and Finland	Age 25-66 years, DBP ≥100 mmHg on two occasions	Secondary hypertension, serum creatinine >150 μmol/, condition requiring β-blocker treatment	Dec 1989 to Apr 1995	All<br>ACEI (Captopril)<br>Conventional: β-blocker (Atenolol or metoprolol) and/or diuretic (HCTZ, bendofluazide)	10,985 (47)<br>5492 (45)<br>5493 (48)	Diuretic and CCB if necessary	Supine DBP <90 mmHg	5.8	6
CASE-J[63 64]	Japan	Age 20-85 years, ≥1 high-risk factor: SBP ≥180 or DBP ≥110 mmHg, T2D, history of angina pectoris, MI, stroke, TIA >6 months	BP ≥200/120 mmHg, T1D, heart failure, ejection fraction <40%, atrial fibrillation, cancer	Sep 2001 to Jan 2003	All<br>ARB (Candesartan)<br>CCB (Amlodipine)	4703 (45)<br>2354 (46)<br>2349 (43)	Allowed to continue background treatment (diuretic, α-blocker, β-	BP (mmHg) by age (years): <60: <130/85; 60-69: <140/90; 70-	3.1	6

Canoy D, *et al. Heart* 2022; 108:1281–1289. doi: 10.1136/heartjnl-2021-320171

		prior to screening, LVH, proteinuria or serum creatinine ≥1.3 mg/100 ml, peripheral artery obstruction					blocker); Can add other treatment except ARB, CCB, ACEI	79: <150/90; ≥80: <160/90		
COLM[65 66]	Japan	Age 65-84 years, hypertension (treated: BP ≥140/90 mmHg; untreated: BP ≥160/100 mmHg), CVD history or CVD risk factors (diabetes, dyslipidaemia)	Secondary/malignant hypertension, recent major CVD, revascularisation, angina pectoris hospitalisation or severe heart failure, atrial fibrillation, hepatic or renal dysfunction	Apr 2007 to Sep 2008	All	5141 (48)	β-blocker, α-blocker, ACEI	BP <140/90 mmHg	3.0	8
					ARB (Olmesartan) and CCB (Amlodipine or azelnidipine)	2568 (48)				
					ARB (Olmesartan) and diuretic (HCTZ, Trichlormethiazide, or indapamide)	2573 (48)				
CONVINCE[67 68]	Multi-country	Age ≥55 years, hypertension, ≥1 CVD risk factor (e.g., diabetes, smoking)	Heart failure, dysrhythmia, secondary hypertension, recent MI or stroke, renal disease, other serious disease, BP ≥190/110 mmHg without treatment	Sep 1996 to Dec 1998	All	16,476 (55)	Additional treatment if necessary	BP <140/90 mmHg	2.8	4
					CCB (Verapamil)	8179 (56)				
					β-blocker (Atenolol) or diuretic (HCTZ)	8297 (56)				
COPE[69]	Japan	Age 40-85 years, BP ≥140/90 mmHg	SBP ≥200 or DBP ≥120 mmHg, secondary hypertension, diabetes, recent CVD or revascularisation, heart failure, atrial fibrillation/flutter, hepatic or renal dysfunction, congenital or rheumatic heart disease, cancer	Jun 2003 to Nov 2006	All	3293 (49)	Additional treatment if necessary	BP <140/90 mmHg	3.6	5
					CCB/ARB (Benidipine/ARB)	1110 (49)				
					CCB/β-blocker (Benidipine/β-blocker)	1089 (49)				
					CCB/Diuretic (Benidipine/Thiazide)	1094 (49)				
E-COST[70]	Japan	Age 35-79 years, BP 140-180/90-110 mmHg	Diabetes, dysglycemia, secondary hypertension, recent MI or stroke, angina pectoris requiring β-blocker treatment, heart failure, left ventricular ejection fraction <40%	Sept to Dec 1999	All	2048 (52)	Additional treatment but not ARB or ACEI	BP <140/90 mmHg	3.1	2
					ARB (Candesartan)	1053 (56)				
					Conventional (mainly CCB and β/α-blocker)	995 (48)				
ELSA[71 72]	Multi-country	Age 45-79 years, BP 150-210/95-115 mmHg	Recent MI or stroke, and T2D	Possibly between 1994 to 1998	All	2334 (45)	Diuretic (HCTZ)	DBP <95 mmHg	3.4	10
					CCB (Lacidipine)	1177 (46)				
					β-blocker (Atenolol)	1157 (45)				
HIJ-CREATE[73]	Japan	Age 20-80 years, CAD hospitalisation and hypertension (BP ≥140/90 mmHg or antihypertensive treatment use)	Secondary hypertension, recent AMI or CeVD, severe aortic valve stenosis, cardiomyopathy, serum creatinine >2 mg/dl, serum potassium >5 mmol/l, hepatic dysfunction, malignancy	Jun 2001 to Apr 2004	All	2049 (20)		BP <130/85 mmHg	4.0	5
					ARB (Candesartan)	1024 (18)				
					Non-ARB (including ACEI)	1025 (21)				
HOMED-BP[20]	Japan	Self-measured SBP 135-179 mmHg or DBP 85-119 mmHg, but not if DBP <65 mmHg or SBP <110 mmHg (clinic SBP <220 mmHg and DBP <125 mmHg)	None specified	May 2001 to Oct 2009	All	3518 (50)	Diuretic; β-blocker; then other drugs (avoid reaching BP <110/65 mmHg)	Either BP <120/80 mmHg or BP 125-134/80-84 mmHg	4.9	8
					ACEI	1172 (50)				
					ARB	1175 (50)				
					CCB	1171 (50)				
IDNT[1]	USA	Age 30-70 years, T2D, hypertension (BP ≥135/85 mmHg or taking antihypertensive drug), proteinuria, serum creatinine of 88 to 265 μmol/l (women) or 106 to 265 μmol/l (men)	None specified	Mar 1996 to Feb 1999	All (except placebo)	1146 (36)	Others except ACEI, ARB and CCB	SBP <135 mmHg (10 mmHg lower if baseline value >145 mmHg); and DBP <85 mmHg	2.6	No data
					ARB (Irbesartan)	579 (35)				
					CCB (Amlodipine)	567 (37)				
INSIGHT[74]	Multi-country	Age 55-80 years, hypertensive (SBP ≥150 or DBP ≥95 mmHg, or SBP ≥160 mmHg), ≥1 other risk factor (TC ≥6.43 mmol/l, smoking, family history of premature MI, CAD, other CVD	None specified	Sep 1994 to Jun 1996	All	6321 (54)	β-blocker or ACEI, then others except CCB or diuretic	SBP/DBP reduction by 20/10 mmHg or SBP/DBP <140/90 mmHg	2.8	15
					CCB (Nifedipine)	3157 (54)				
					Diuretic (Co-amilozide [HCTZ + amiloride])	3164 (53)				
INVEST[75]	Multi-country	Age ≥50 years, documented CAD, essential hypertension requiring drug therapy, heart failure Class I-III[b]	Patients taking β-blocker within two weeks of randomisation or for recent MI	From Jan 1998	All	21,230 (52)	ACEI (Trandolapril) and/or diuretic (HCTZ)	SBP/DBP <140/90 mmHg; <130/85 mmHg with diabetes or renal impairment	2.8	5
					CCB (Verapamil)	10,648 (52)				
					Non-CCB (Atenolol)	10,672 (52)				
JMIC-B[76]	Japan				All	1650 (31)		BP <150/90 mmHg	2.3	5

Canoy D, *et al. Heart* 2022; 108:1281–1289. doi: 10.1136/heartjnl-2021-320171

		Age <75 years, hypertension (BP ≥160/≥95 mmHg or both SBP ≥150 and DBP ≥90 mmHg, or antihypertensive treatment), CAD or meeting both criteria: history of >2 anginal attacks per week with stable frequency and ST-segment depression of ≥1 mm on stress test (or detection of MI with myocardial scintigraphy)	MI, unstable angina, DBP ≥120 mmHg, secondary hypertension, symptomatic CeVD, heart failure, atrial fibrillation/arrhythmias, renal or hepatic dysfunction, uncontrollable diabetes and familial hypercholesterolaemia	Jan 1994 to Jul 1997	CCB (Nifedipine)	828 (32)	α-blocker (doxazosin, bunazosin or prazosin); nitrates or β-blocker for angina if needed			
					ACEI (Enalapril, imidapril or lisinopril)	822 (30)				
LIFE[77,78]	Multi-country	Age 55-80 years, hypertension (SBP 160-200 mmHg; DBP 95-115 mmHg), electrocardiogram signs of LVH	Secondary hypertension, recent MI or stroke, angina pectoris requiring treatment, heart failure or left ejection fraction ≤40%	June 1995 to May 1997	All	9193 (54)	Diuretic (HCTZ) and other antihypertensive treatment except ACEI, ARB and β-blocker	BP 140/90 mmHg	4.9	15
					ARB (Losartan)	4605 (54)				
					β-blocker (Atenolol)	4588 (54)				
MOSES[79]	Germany and Austria	Hypertension requiring treatment, documented TIA, ischaemic stroke or cerebral haemorrhage	Internal carotid artery occlusion or stenosis >70%, heart failure, age >85 years, on anticoagulant for cardiac arrhythmia, high-grade aortic or mitral valve stenosis, unstable angina	Oct 1998 to Feb 2002	All	1352 (46)	Diuretic, β-blocker, α-blocker or centrally-acting drugs; ACEI, ARB or CCB only if clinically necessary	BP <140/90 mmHg	3.3	7
					ARB (Eprosartan)	681 (46)				
					CCB (Nitrendipine)	671 (45)				
NICS-EH[80]	Japan	Age ≥60 years, SBP 160-220 mmHg and DBP <115 mmHg and no cardiovascular complications	None specified	Oct 1989 to Apr 1992	All	414 (67)	Titration but no additional treatment	BP response sufficient as determined by the investigator	3.2	3
					CCB (Nicardipine)	204 (60)				
					Diuretic (Trichlormethiazide)	210 (74)				
NORDIL[81]	Norway and Sweden	Age 50-74 years, untreated hypertension (DBP ≥100 mmHg on two occasions); if previously treated, DBP ≥100 mmHg on two consecutive visits at one week apart during run-in period and no treatment was given	Age <50 or ≥70y, bradycardia, secondary hypertension, atrial fibrillation, recent CeVD or MI, heart failure	Oct 1992 to Oct 1999	All	10,881 (51)	CCB group: plus ACEI, diuretic, α-blocker or any other if necessary; β-blocker group: ACEI or α-blocker or any other drug except CCB if necessary		4.2	8
					CCB (Diltiazem)	5410 (51)				
					β-blocker and/or diuretic (Thiazide)	5471 (51)				
ONTARGET[54,82]	Multi-country	CAD, PAD, CeVD or diabetes with end-organ damage	Heart failure, pericarditis, congenital heart disease, unexplained syncope , planned revascularisation <3 months of consent, uncontrolled hypertension, heart transplant, subarachnoid haemorrhage, renal artery disease, proteinuria, hepatic dysfunction, volume or sodium depletion, primary hyper-aldosteronism, hereditary fructose intolerance, other serious conditions	Jan 2002 to Aug 2003	All	25,620 (27)	None	None specified	4.8	9
					ACEI (Ramipril)	8576 (27)				
					ARB (Telmisartan)	8542 (26)				
					ACEI (Ramipril) and ARB (Telmisartan)	8502 (26)				
STOP Hyper-tension-2[83]	Sweden	Aged 70-84 years, SBP ≥180 mmHg and/or DBP ≥105 mmHg	Not specified	Sep 1992 to Dec 1994 1987 to 1991	All	6614 (67)		BP <160/95 mmHg	4.5	10
					Conventional: β-blocker (Atenolol or metoprolol), diuretic (HCTZ) or both	2213 (68)				
					ACEI (Enalapril or lisinopril	2205 (66)				
					CCB (Felodipine or isradipine)	2196 (66)				
UKPDS[22-24]	UK	Age 25-65 years, newly-diagnosed diabetes, and hypertension (untreated: SBP ≥160 mmHg and/or DBP ≥90 mmHg; treated: SBP ≥150 mmHg and/or DBP ≥85 mmHg)	Ketonuria, recent MI, angina, heart failure, >1 major vascular episode, serum creatinine >175 umol/L, retinopathy, malignant hypertension, uncorrected endocrine abnormality, severe concurrent illness		All	758 (46)	Sequentially: Diuretic (furosemide), CCB (nifedipine), methyldopa, ARB (prazosin)	BP <150/85 mmHg	7.9	5
					ACEI (Captopril)	400 (49)				
					β-blocker (Atenolol)	358 (43)				
VALUE[84,85]	Multi-country	Age ≥50 years, hypertension, CVD, CVD risk factors (male sex, age >50 years, diabetes, current smoking, high	Renal artery stenosis, recent CAD or CeVD, severe hepatic disease or chronic renal failure, heart failure, on	Sept 1997 to Dec 1999	All	15,245 (42)	Diuretic (HCTZ, then other antihypertensive drugs except ARB (ACEI	BP <140/90 mmHg	4.2	12
					ARB (Valsartan-based)	7649 (42)				
					CCB (Amlodipine-based)	7596 (42)				

Canoy D, *et al. Heart* 2022; 108:1281–1289. doi: 10.1136/heartjnl-2021-320171

		cholesterol, LVH, proteinuria, serum creatinine 150 to 265 μmol/l)	monotherapy with β-blocker for CAD and hypertension				or CCB if clinically indicated other than for hypertension)			
VHAS[86]	Italy	Age 40-65 years, BP ≥160/95 mmHg	Secondary hypertension, recent stroke or TIA, CAD, PAD, bradycardia, arrhythmias, heart failure, renal or hepatic dysfunction, hyperuricaemia, hypokalemia, T1D, familial dyslipidemia, serious concomitant disease	Probably early 1990s (publication in 1997)	All	1414 (51)	ACEI (Captopril)	Sitting DBP ≤90 mmHg; ≤95 mmHg if lowered by ≥10% from baseline values	1.7	2
					CCB (Verapamil)	707 (50)				
					Diuretic (Chlorthalidone)	707 (52)				

Trial name acronyms are described in full in the Trial acronym legend in the Supplement; Data on participant numbers and proportions, mean duration of follow-up and mean number of follow-up blood pressure measurements are from the datasets provided to the collaboration; BP – blood pressure; GFR – glomerular filtration rate; DBP – diastolic blood pressure; CKD – chronic kidney disease; T2D – type 2 diabetes; CAD – coronary artery disease; CeVD – cerebrovascular disease; CVD – cardiovascular disease; HbA1c - glycated haemoglobin; SBP – systolic blood pressure; TIA – transient ischaemic attack; PAD – peripheral artery disease; MI – myocardial infarction; CNS – central nervous system; LVEF – low ventricular ejection fraction; HDL – high-density lipoprotein; ECG – electrocardiogram; TC – total cholesterol; LVH – left ventricular hypertrophy; T1D – type 1 diabetes; CCB – calcium channel-blocker; ACEI – angiotensin-converting enzyme inhibitor; ARB – angiotensin II receptor blocker; HCTZ – hydrochlorothiazide; [a]Excludes 534 participants with heart failure at baseline; [b]Excludes 1346 participants with heart failure at baseline.

Table S4. Risk of bias assessment of each trial.

Trial	Risk of bias arising from randomisation	Risk of bias due to effect of assignment to intervention	Risk of bias due to missing outcome data	Risk of bias due to measurement of outcome	Risk of bias due to reporting of result	Overall risk of bias
AASK	Low	Low	Low	Low	Low	Low
ABCD	Low	Low	Low	Low	Low	Low
ACCORD	Low	Some	Low	Low	Low	Low
ACTIVE I	Low	Low	Low	Low	Low	Low
ADVANCE	Low	Low	Low	Low	Low	Low
ALLHAT	Low	Low	Low	Low	Low	Low
ANBP	Low	Low	Low	Low	Low	Low
ANBP2	Low	Some	Low	Low	Low	Low
ASCOT-BPLA	Low	Some	Low	Low	Low	Low
BENEDICT	Low	Low	Low	Low	Low	Low
CAMELOT	Low	Low	Low	Low	Low	Low
CAPPP	Low	Some	Low	Low	Low	Low
Cardio-Sis	Low	Some	Some	Low	Low	Some
CASE-J	Low	Some	Low	Low	Low	Low
COLM	Low	Some	Low	Low	Low	Low
CONVINCE	Low	Low	Low	Low	Low	Low
COPE	Low	Some	Low	Low	Low	Low
DIABHYCAR	Low	Low	Low	Low	Low	Low
Dutch TIA Trial	Low	Low	Low	Low	Low	Low
E-COST	High	Some	Low	Low	Low	High
ELSA	Low	Low	Low	Low	Low	Low
EUROPA	Low	Low	Low	Low	Low	Low
EWPHE	Low	Low	Low	Low	Low	Low
HDFP	Some	Some	Low	Low	Low	Low
HIJ-CREATE	Low	Some	Low	Low	Low	Low
HOMED-BP	Low	Some	Low	Low	Low	Low
HOPE	Low	Some	Low	Low	Low	Low
HYVET	Low	Low	Low	Low	Low	Low
IDNT	Low	Low	Some	Low	Low	Some
INSIGHT	Low	Low	Low	Low	Low	Low
INVEST	Low	Some	Low	Low	Low	Low
JMIC-B	Low	Some	Low	Low	Low	Low
LIFE	Low	Low	Low	Low	Low	Low
MOSES	Low	Some	Low	Low	Low	Low
NICS-EH	Low	Some	Low	Low	Low	Some
NORDIL	Low	Some	Low	Low	Low	Low
ONTARGET	Low	Some	Low	Low	Low	Low
PART 2	Low	Low	Low	Low	Low	Low
PEACE	Low	Low	Low	Low	Low	Low
PREVEND IT	Low	Low	Low	Low	Low	Low
PREVENT	Low	Low	Low	Low	Low	Low
PRoFESS	Low	Low	Low	Low	Low	Low
PROGRESS	Low	Low	Low	Low	Low	Low
SHEP	Low	Low	Low	Low	Low	Low
SPRINT	Low	Some	Low	Low	Low	Low
STOP Hypertension-2	Low	Some	Low	Low	Low	Low
SYST-EUR	Low	Some	Low	Low	Low	Low
TRANSCEND	Low	Low	Low	Low	Low	Low
UKPDS	Low	Some	Low	Low	Low	Low
VALISH	Low	Low	Low	Low	Low	Low
VALUE	Low	Low	Low	Low	Low	Low
VHAS	Low	Low	Low	Low	Low	Low

Trial name acronyms are described in full in the Trial acronym legend in the Supplement.

Table S5. Indicators of numbers of blood pressure-lowering drug classes given and adherence to assigned treatment, separately by trial design.

A. Blood pressure-lowering intensity trials

Trial	Comparison groups	Number of drugs given		Adherence to randomised treatment assignment during follow-up	
		No.	Description	%	Description
**AASK**	Less intense	3	Mean number of drugs given	81.7	On randomised treatment
	More intense	2.4	Mean number of drugs given	80.1	On randomised treatment
**ABCD**[†]	Less intense	-	Not reported	-	Mean achieved SBP during last 4 years of follow-up: 137 mmHg
	More intense	-	Not reported	-	Mean achieved SBP during last 4 years of follow-up: 128 mmHg
**ACCORD**	Less intense	2.1	Mean number of drugs given after year 1 follow-up	-	Mean achieved SBP after year 1 follow-up: 134 mmHg
	More intense	3.4	Mean number of drugs given after year 1 follow-up	-	Mean achieved SBP after year 1 follow-up: 119 mmHg
**Cardio-Sis**	Less intense	2.9	Mean number of drugs given at year 2 follow-up	-	Mean achieved SBP at end of study: 136 mmHg
	More intense	2.9	Mean number of drugs given at year 2 follow-up	-	Mean achieved SBP at end of study: 132 mmHg
**HDFP**	Less intense	58%	Taking antihypertensive drugs at year 5 follow-up	43.5	Achieved at or below DBP goal
	More intense	78%	Taking antihypertensive drugs at year 5 follow-up	64.9	Achieved at or below DBP goal
**HOMED-BP**	Less intense	1.74	Mean defined daily dose at last follow-up	68.3	Meeting target SBP (125 to 134 mmHg) at end of study
	More intense	1.82	Mean defined daily dose at last follow-up	42.6	Meeting target SBP (<125 mmHg) at end of study
**SPRINT**	Less intense	1.8	Mean number of drugs given	-	Mean achieved SBP at end of study: 135 mmHg
	More intense	2.7	Mean number of drugs given	-	Mean achieved SBP at end of study: 122 mmHg
**UKPDS**	Less intense	11%	Taking ≥3 drug classes at year 9 follow-up	43.0	% of total person-years not receiving treatment
	More intense	29%	Taking ≥3 drug classes at year 9 follow-up	77.0	% of total person-years on assigned treatment
**VALISH**	Less intense	1.6	Mean number of drugs given at end of follow-up	-	Mean achieved SBP at end of study: 142 mmHg
	More intense	1.6	Mean number of drugs given at end of follow-up	-	Mean achieved SBP at end of study: 137 mmHg

Canoy D, *et al. Heart* 2022; 108:1281–1289. doi: 10.1136/heartjnl-2021-320171

Table S5. Indicators of numbers of blood pressure-lowering drug classes given and adherence to assigned treatment, separately by trial design (cont'd).

B. Placebo-controlled trials

Trial	Comparison groups	Number of drugs given		Adherence to randomised treatment assignment during follow-up	
		No.	Description	%	Description
**ACTIVE I**	Placebo	2.15	Mean number of non-study drugs at year 2	69.7	Did not discontinue assigned treatment
	ARB	2	Mean number of non-study drugs at year 2	69.7	Did not discontinue assigned treatment
**ADVANCE**	Placebo	1.57	Mean number of non-study drugs by end of follow-up	74.0	On randomised treatment by end of follow-up
	ACEI and diuretic	1.28	Mean number of non-study drugs by end of follow-up	73.0	On randomised treatment by end of follow-up
**ANBP**	Placebo	1.52	Mean number of drugs given (calculated from numbers taking 1, 2 or ≥2 corresponding placebo)	63.3	On randomised treatment by end of study
	Diuretic	1.82	Mean number of drugs given (calculated from numbers taking 1, 2 or ≥2 drugs)	66.1	On randomised treatment by end of study
**BENEDICT**	Placebo	2.18	Mean number of drugs given	-	Not reported
	ACEI	1.99	Mean number of drugs given	-	Not reported
	CCB	2.03	Mean number of drugs given	-	Not reported
	ACEI and CCB	1.87	Mean number of drugs given	-	Not reported
**CAMELOT**	Placebo	-	NA	68.9	Did not discontinue assigned treatment
	CCB	8.6	Mean dose received (g)	70.7	Did not discontinue assigned treatment
	ACEI	17.4	Mean dose received (g)	64.9	Did not discontinue assigned drug
**DIABHYCAR**	Placebo	0.22	Mean number of non-study drugs given	59.0	On randomised treatment by end of study
	ACEI	0.2	Mean number of non-study drugs given	59.3	On randomised treatment by end of study
**Dutch-TIA**	Placebo	-	Not reported	67.7	On randomised treatment at year 3 follow-up
	ß-blocker	-	Not reported	64.2	On randomised treatment at year 3 follow-up
**EUROPA**	Placebo	-	NA	84.0	On randomised treatment at year 3 follow-up
	ACEI	7.05	Maximum mean daily dose (mg)	81.0	On randomised treatment at year 3 follow-up
**EWPHE**	Placebo	0.63	Mean number of non-study drugs given	63.0	Did not discontinue assigned treatment
	Diuretic	0.35	Mean number of non-study drugs given	64.2	Did not discontinue assigned treatment
**HOPE**	Placebo	0.18	Mean number of non-study drugs given	87.7	On randomised treatment at final visit (12.3 % received intervention drug)
	ACEI	0.14	Mean number of non-study drugs given	78.8	On randomised treatment at final visit
**HYVET**	Placebo	-	NA	-	Not reported
	Diuretic	0.73	Mean number of non-study drug given at year 2	-	Not reported
**IDNT**	Placebo	3.3	Mean number of non-study drugs given	-	Not reported
	ARB and CCB	3	Mean number of non-study drugs given	-	Not reported

Canoy D, *et al. Heart* 2022; 108:1281–1289. doi: 10.1136/heartjnl-2021-320171

**PART 2**	Placebo	7	% given non-study drug at year 4 follow-up	75.0	On randomised treatment at year 4 follow-up
	ACEI	2.9	% given non-study drug at year 4 follow-up	72.0	On randomised treatment at year 4 follow-up
**PEACE**	Placebo	8.3	% taking non-study drug at year 3	77.7	On randomised treatment at year 3 follow-up
	ACEI	74.5	% taking study and non-study drug (same class) at year 3	68.6	On randomised treatment at year 3 follow-up
**PREVEND IT**	Placebo	-	Not reported	63.3	Compliance above 75% at year 4 follow-up
	ACEI	-	Not reported	70.7	Compliance above 75% at year 4 follow-up
**PREVENT**	Placebo	0.67	Mean number of non-study drugs given	83.0	Pill count compliance
	CCB	0.56	Mean number of non-study drugs given	79.0	Pill count compliance
**PRoFESS**	Placebo	2.5	% on non-study ARB at penultimate visit (≥30% on non-study BP-lowering drugs by end of study)	70.8	% on randomised treatment at year 3 follow-up
	Telmisartan	2.3	% on non-study ARB at final visit (≥25% on non-study BP-lowering drugs by end of study)	68.3	% on randomised treatment at year 3 follow-up
**PROGRESS**	Placebo	-	Not reported	87.0	On randomised treatment by end of follow-up
	ACEI and/or diuretic	-	Not reported	86.0	On randomised treatment by end of follow-up
**SHEP**	Placebo	44.4	% given study drug at year 5 follow-up	55.6	On randomised treatment at year 5 follow-up
	ß-blocker and diuretic	-	Over two-thirds given step 1 and/or step 2 drug at year 5 follow-up	89.7	On randomised treatment at year 3 follow-up
**SYST-EUR**	Placebo	-	Some given treatment but could not be estimated	47.7	On randomised treatment at year 4 follow-up
	CCB	1.35	Mean number of drugs given	60.4	On randomised treatment at year 4 follow-up
**TRANSCEND**	Placebo	1.6	Mean number of non-study drugs given by end of study	40.7	On randomised treatment by end of study (more may have received non-study drugs)
	ARB	1.4	Mean number of non-study drugs given by end of study	80.8	On randomised treatment by end of study

Canoy D, *et al. Heart* 2022; 108:1281–1289. doi: 10.1136/heartjnl-2021-320171

Table S5. Indicators of numbers of blood pressure-lowering drug classes given and adherence to assigned treatment, separately by trial design (cont'd).

C. Drug class comparison trials

Trial	Comparison groups	Number of drugs given		Adherence to randomised treatment assignment during follow-up	
		No.	Description	%	Description
**AASK**	ACEI	2.7	Mean number of drugs given	76.8	On randomised treatment during follow-up
	CCB	2.7	Mean number of drugs given	83.4	On randomised treatment during follow-up
	β-blocker	2.8	Mean number of drugs given	83.6	On randomised treatment during follow-up
**ABCD**‡	CCB	2.8	Mean number of drugs given	45.1	Did not discontinue assigned treatment
	ACEI	2.9	Mean number of drugs given	39.6	Did not discontinue assigned treatment
**ALLHAT**	Diuretic	1.8	Mean number of drugs given	71.2	On randomised treatment at year 5 follow-up
	CCB	1.9	Mean number of drugs given	72.1	On randomised treatment at year 5 follow-up
	ACEI	2	Mean number of drugs given	61.2	On randomised treatment at year 5 follow-up
	α-blocker[87]	1.2	Mean number of drugs given at year 4 follow-up	75.0	On randomised treatment at year 4 follow-up
**ANBP2**	ACEI	6.0%	Given ≥3 drugs	58.0	On randomised treatment by end of study
	Diuretic	5.0%	Given ≥3 drugs	62.0	On randomised treatment by end of study
**ASCOT-BPLA**	CCB-based	2.2	Mean number of drugs given	50.0	On randomised treatment (with and without other antihypertensive drugs)
	β-blocker-based	2.3	Mean number of drugs given	55.0	On randomised treatment (with and without other antihypertensive drugs)
**BENEDICT**	Placebo	2.18	Mean number of drugs given	-	Not reported
	ACEI	1.99	Mean number of drugs given	-	Not reported
	CCB	2.03	Mean number of drugs given	-	Not reported
	ACEI and CCB	1.87	Mean number of drugs given	-	Not reported
**CAMELOT**	Placebo	-	NA	69.3	Did not discontinue assigned drug
	CCB	8.6	Mean dose received (g)	70.7	Did not discontinue assigned drug
	ACEI	17.4	Mean dose received (g)	64.9	Did not discontinue assigned drug
**CAPPP**	β-blocker and/or diuretic	-	Not reported	-	Not reported
	ACEI	-	No reported	-	Not reported
**CASE-J**	ARB	1.5	Mean number of drugs given	96.5	Receiving >80% of assigned drug during follow-up
	CCB	1.4	Mean number of drugs given	96.0	Receiving >80% of assigned drug during follow-up
**COLM**	ARB and CCB	2.1	Mean number of drugs given	-	Not reported
	ARB and diuretic	2.1	Mean number of drugs given	-	Not reported
**CONVINCE**	CCB	-	Proportion given >1 drug increased over time	60.6	Did not discontinue assigned drug

Canoy D, *et al. Heart* 2022; 108:1281–1289. doi: 10.1136/heartjnl-2021-320171

	β-blocker or diuretic	-	Proportion given >1 drug increased over time	60.3	Did not discontinue assigned drug
**COPE**	CCB and ARB	1.22	Mean number of drugs given	78.6	Did not discontinue assigned drug
	CCB and β-blocker	1.26	Mean number of drugs given	74.1	Did not discontinue assigned drug
	CCB and diuretic	1.30	Mean number of drugs given	76.2	Did not discontinue assigned drug
**E-COST**	Conventional	71.5%	Taking ≥3 drugs	81.9	On randomised treatment by end of study
	ARB	21.5%	Taking ≥3 drugs	77.3	On randomised treatment by end of study
**ELSA**	CCB	31.8%	Taking additional drug (diuretic)	-	Not reported
	β-blocker	35.9%	Taking additional drug (diuretic)	-	Not reported
**HIJ-CREATE**	non-ARB	2.8	Mean number of drugs given	77.0	On randomised treatment (prescribed ARB at the end of study)
	ARB	2	Mean number of drugs given	97.5	On randomised treatment (prescribed ACEI at end of study)
**HOMED-BP**	ACEI	-	Not reported	-	Not reported
	ARB	-	Not reported	-	Not reported
	CCB	-	Not reported	-	Not reported
**IDNT**	ARB	4	Mean number of drugs given	-	Not reported
	CCB	4	Mean number of drugs given	-	Not reported
**INSIGHT**	Diuretic	1.72	Minimum mean number of drugs given at year 3	78.1	On randomised treatment at year 3 follow-up
	CCB	1.65	Minimum mean number of drugs given at year 3	70.7	On randomised treatment at year 3 follow-up
**INVEST**	CCB	1.4	Mean number of drugs given at year 2 follow-up	81.5	On randomised treatment at year 2 follow-up (Step 1 drug only)
	non-CCB	1.4	Mean number of drugs given at year 2 follow-up	77.5	On randomised treatment at year 2 follow-up (Step 1 drug only)
**JMIC-B**	CCB	2.02	Mean number of drugs given	-	Not reported
	ACEI	1.03	Mean number of drugs given	-	Not reported
**LIFE**	ARB	2.15	Minimum mean number of drugs	84.0	On randomised treatment
	β-blocker	2.11	Minimum mean number of drugs	80.0	On randomised treatment
**MOSES**	CCB	1.34	Minimum mean number of drugs	-	Not reported
	ARB	1.44	Minimum mean number of drugs	-	Not reported
**NICS-EH**	Diuretic	1.1	Mean number of drugs given	-	Not reported
	CCB	1.1	Mean number of drugs given	-	Not reported
**NORDIL**	CCB	1.4	Mean number of drugs given	77.0	On randomised treatment
	β-blocker and/or diuretic	1.5	Mean number of drugs given	93.0	On randomised treatment
**ONTARGET**	ARB	1.1	Mean number of drugs given	85.6	On randomised treatment at year 4 follow-up
	ACEI	1	Mean number of drugs given	84.7	On randomised treatment at year 4 follow-up
	ARB and ACEI	1.1	Mean number of drugs given	73.6	On randomised treatment at year 4 follow-up

Canoy D, *et al. Heart* 2022; 108:1281–1289. doi: 10.1136/heartjnl-2021-320171

**STOP Hypertension-2**	β-blocker and/or diuretic	1.6	Mean number of drugs given	62.3	On randomised treatment at final visit
	ACEI	1.6	Mean number of drugs given	61.3	On randomised treatment at final visit
	CCB	1.6	Mean number of drugs given	66.2	On randomised treatment at final visit
**UKPDS**	ACEI	27%	≥3 agents in tight control	78.0	On randomised treatment at final visit
	β-blocker	31%	≥3 agents in tight control	65.1	On randomised treatment at final visit
**VALUE**	ARB-based	2.1	Mean number of drugs given	73.7	On randomised treatment during follow-up
	CCB-based	2	Mean number of drugs given	74.9	On randomised treatment during follow-up
**VHAS**	CCB	1.2	Mean number of drugs given	44.1	On randomised treatment (staying on monotherapy) at year 2 follow-up
	Diuretic	1.3	Mean number of drugs given	38.8	On randomised treatment (staying on monotherapy) at year 2 follow-up

Trial name acronyms are described in full in the Trial acronym legend in the Supplement; SBP – systolic blood pressure; CCB – calcium channel-blocker; ACEI – angiotensin-converting enzyme inhibitor; ARB – angiotensin II receptor blocker; †Normotensive patient cohort only; ‡Hypertensive patient cohort only.

Canoy D, *et al. Heart* 2022; 108:1281–1289. doi: 10.1136/heartjnl-2021-320171

Table S6. Characteristics of participants at baseline for each trial.

Trial	Participants, N (% women)	Age (years), mean (SD)	SBP (mmHg), mean (SD)	DBP (mmHg), mean (SD)	Body mass index (kg/m²), mean (SD)	% Current smokers (N)	% Caucasian / European (N)	% Prevalent disease (N)				
								Cardiovascular disease	Coronary heart disease	Cerebrovascular disease/stroke	Chronic kidney disease	Diabetes
AASK	1094 (39)	54 (11)	150 (24)	95 (10)	30.6 (6.6)	29 (321)	0 (0)	52 (564)	52 (564)	-	100 (1094)	0 (0)
ABCD	950 (39)	58 (8)	155 (17)	98 (10)	31.7 (5.7)	14 (65)	66 (310)	9 (89)	9 (41)	2 (11)	19 (90)	100 (470)
ACCORD	4733 (48)	63 (7)	139 (15)	76 (10)	32.1 (5.5)	13 (626	59 (2781)	34 (1593)	14 (650)	6 (307)	0 (0)	100 (4733)
ACTIVE	9016 (39)	70 (10)	138 (17)	82 (10)	29.1 (5.8)	8 (698)	75 (6769)	35 (3177)	2294 (25.4%)	14 (1230)	0 (0)	20 (1785)
ADVANCE	11,140 (43)	66 (6)	145 (22)	81 (10)	28.3 (5.2)	14 (1550)	60 (6687)	31 (3461)	2380 (21.4%)	13 (1438)	-	100 (11,140)
ALLHAT	42,418 (47)	67 (8)	146 (16)	84 (10)	29.6 (5.9)	22 (9269)	51 (21561)	46 (19,389)	-	-	-	36 (15,283)
ANBP	3427 (37)	50 (9)	157 (15)	100 (10)	26.7 (3.9)	26 (891)	-	0 (16)	0 (16)	0 (0)	7 (240)	0 (0)
ANBP2	6083 (51)	73 (5)	168 (13)	91 (10)	27.1 (4.2)	7 (431)	98 (5984)	8 (474)	4 (229)	5 (276)	0 (6)	7 (441)
ASCOT-BPLA	19,257 (23)	63 (9)	164 (18)	95 (10)	28.7 (4.6)	33 (6277)	95 (18357)	36 (7008)	27 (5284)	11 (2121)	62 (12,017)	27 (5145)
BENEDICT	1204 (47)	62 (8)	151 (14)	88 (10)	29.1 (4.7)	12 (146)	100 (1204)	-	-	-	0 (0)	100 (1209)
CAMELOT	1991 (26)	58 (10)	129 (16)	78 (10)	29.8 (5.3)	26 (528)	89 (1783)	93 (1858)	93 (1857)	4 (81)		18 (364)
CAPPP	10,985 (47)	52 (8)	161 (20)	99 (10)	27.8 (4.4)	22 (2431)	-	3 (351)	2 (201)	1 (160)	0 (28)	5 (572)
Cardio-Sis	1111 (59)	67 (7)	158 (9)	87 (10)	27.8 (4.2)	20 (226)	-	18 (202)	11 (128)	8 (91)	0 (0)	0 (0)
CASE-J	4703 (45)	64 (11)	163 (14)	92 (10)	24.5 (3.7)	22 (1025)	0 (0)	22 (1028)	13 (596)	10 (473)	58 (2720)	43 (2018)
COLM	5141 (48)	74 (5)	158 (13)	87 (10)	24.3 (3.4)	11 (551)	0 (0)	24 (1225)	11 (563)	15 (751)	2 (108)	26 (1362)
CONVINCE	16,476 (56)	66 (7)	150 (16)	87 (10)	-	23 (3795)	84 (13845)	27 (4458)	23 (3820)	6 (1019)	0 (0)	20 (3239)
COPE	3293 (49)	64 (11)	154 (12)	89 (10)	24.5 (3.4)	21 (700)	0 (0)	7 (219)	3 (109)	4 (126)	46 (1530)	0 (0)
DIABHYCAR	4912 (30)	65 (8)	145 (15)	82 (10)	29.2 (4.6)	15 (756)	-	15 (739)	15 (739)	-	-	100 (4912)
Dutch TIA Trial	1473 (36)	64 (10)	157 (25)	91 (10)	-	47 (693)	-	100 (1473)	9 (138)	100 (1473)	100 (1711)	5 (79)
E-COST	2048 (53)	64 (11)	164 (18)	94 (10)	-	-	0 (0)	10 (213)	-	10 (213)	11 (228)	0 (0)
ELSA	2334 (46)	57 (7)	160 (15)	98 (10)	27.2 (3.8)	20 (478)	98 (2294)	13 (305)	13 (305)	-	7 (163)	4 (98)
EUROPA	12,218 (15)	61 (9)	137 (16)	82 (10)	27.4 (3.5)	15 (1862)	99 (12,064)	100 (12,218)	100 (12,218)	2 (222)	0 (0)	-
EWPHE	840 (70)	71 (8)	183 (17)	101 (10)	26.4 (4.5)	17 (143)	-	15 (124)	9 (73)	8 (63)	0 (2)	72 (8.6%)
HDFP	10,940 (45)	51 (10)	159 (23)	101 (9)	-	39 (4248)	55 (6021)	7 (780)	5 (561)	3 (274)	-	7 (772)
HIJ-CREATE	2049 (20)	65 (9)	135 (18)	76 (10)	24.6 (3)	25 (509)	-	100 (2049)	100 (2049)	10 (205)	0 (3)	38 (780)
HOMED-BP	3518 (50)	60 (10)	154 (18)	90 (10)	24.4 (3.5)	21 (743)	0 (0)	3 (106)	2 (61)	1 (46)	-	15 (538)
HOPE	9297 (27)	66 (7)	139 (20)	79 (10)	27.7 (4.4)	14 (1319)	90 (8343)	80 (7477)	80 (7477)	-	-	39 (3577)
HYVET	3845 (60)	84 (3)	173 (9)	91 (10)	24.7 (3.7)	7 (253)	-	10 (374)	3 (121)	7 (261)	-	10 (388)
IDNT	1715 (34)	59 (8)	159 (20)	87 (11)	30.8 (5.8)	-	73 (1241)	-	-	-	100 (1715)	100 (1715)

INSIGHT	6321 (54)	65 (6)	173 (15)	99 (10)	28.2 (4.6)	28 (1793)	-	11 (671)	11 (671)	-	-	21 (1302)
INVEST	21,320 (51)	66 (10)	151 (20)	87 (10)	29.2 (7.1)	12 (2809)	48 (10,925)	100 (21,320)	100 (21,320)	7 (1629)	2 (424)	28 (6400)
JMIC-B	1650 (31)	65 (85)	146 (19)	82 (10)	24 (2.9)	34 (563)	0	100 (1650)	100 (1650)	-	-	23 (372)
LIFE	9193 (54)	67 (7)	174 (14)	98 (10)	28 (4.8)	16 (1499)	92 (8503)	19 (1771)	16 (1469)	4 (401)	-	13 (1195)
MOSES	1352 (46)	68 (10)	151 (18)	87 (10)	27.5 (4.3)	18 (247)	100 (1352)	100 (1352)	26 (355)	100 (1352)	5 (72)	37 (498)
NICS-EH	414 (67)	70 (7)	172 (12)	94 (10)	23.4 (3.1)	9 (38)	0	4 (16)	4 (0.9%)	3 (12)	-	4 (17)
NORDIL	10,881 (51)	60 (7)	173 (18)	106 (10)	27.8 (4.3)	22 (2442)	-	7 (740)	496 (4.6%)	2 (271)	0 (31)	7 (727)
ONTARGET	25,620 (27)	67 (7)	142 (17)	82 (10)	28.2 (4.8)	13 (3225)	73 (18,708)	87 (22,315)	75 (19,102)	70 (17,927)	-	38 (9612)
PART 2	617 (18)	60 (8)	133 (17)	79 (10)	26.8 (3.6)	16 (100)	100 (615)	74 (457)	68 (420)	10 (62)	-	8 (51)
PEACE	8290 (18)	64 (8)	133 (17)	77 (10)	-	14 (1177)	-	100 (8290)	100 (8290)	4 (357)	-	17 (1380)
PREVEND IT	864 (35)	51 (12)	130 (18)	76 (10)	26.4 (4.4)	40 (345)	96 (830)	3 (24)	2 (13)	2 (13)	100 (864)	3 (22)
PREVENT	825 (20)	57 (10)	129 (17)	79 (10)	28 (4.8)	25 (204)	89 (732)	100 (825)	100 (825)	7 (55)	1 (8)	12 (98)
PRoFESS	19,798 (36)	66 (8)	144 (17)	84 (11)	26.8 (5)	21 (4231)	57 (11,200)	100 (19,798)	15 (2933)	100 (19,798)	-	28 (5587)
PROGRESS	6105 (30)	64 (10)	147 (19)	86 (10)	25.7 (3.8)	21 (1279)	0 (9)	100 (6105)	16 (983)	100 (6105)	-	13 (761)
SHEP	4736 (57)	72 (7)	170 (9)	77 (10)	27.1 (4.8)	13 (597)	79 (3731)	6 (284)	5 (232)	0 (66)	0 (0)	10 (476)
SPRINT	9361 (36)	68 (9)	140 (15.6)	78 (10)	29.9 (5.8)	13 (1240)	58 (5399)	20 (1877)	20 (1877)	0 (0)	28 (2646)	0 (0)
STOP Hypertension 2	6614 (67)	76 (4)	194 (15)	98 (10)	26.7 (4)	9 (594)	-	16 (1072)	10 (647)	8 (502)	-	11 (719)
SYST-EUR	4695 (67)	70 (7)	174 (10)	86 (10)	27 (4.1)	7 (343)	-	6 (286)	3 (164)	3 (124)	0 (20)	10 (449)
TRANSCEND	5926 (43)	68 (7)	141 (17)	82 (10)	28.2 (4.8)	10 (582)	61 (3621)	88 (5222)	75 (4418)	71 (4207)	-	36 (2118)
UKPDS	1148 (45)	56 (8)	159 (17)	93 (10)	29.6 (5.5)	23 (256)	87 (1000)	3 (35)	1 (15)	2 (20)	-	100 (1148)
VALISH	3079 (62)	76 (4)	169 (9)	82 (10)	23.5 (3.4)	15 (450)	-	12 (371)	6 (194)	7 (202)	31 (944)	13 (399)
VALUE	15,245 (42)	67 (8)	154 (19)	87 (10)	28.6 (5)	24 (3664)	89 (13,617)	60 (9169)	46 (6981)	20 (3014)	-	32 (4823)
VHAS	1414 (51)	54 (7)	169 (10)	102 (10)	27.1 (4.1)	18 (256)	100 (1414)	12 (164)	-	-	3 (48)	8 (108)

Acronyms are described in full in the Trial acronym legend in the Supplement; SBP – systolic blood pressure; DBP – diastolic blood pressure.

Canoy D, *et al. Heart* 2022; 108:1281–1289. doi: 10.1136/heartjnl-2021-320171

Table S7. Blood pressure measurement methods used in the trials included in the BPLTTC.

Study	Specified method of blood pressure measurement
AASK	Mean of last two readings of three consecutive seated BP readings after five minutes rest. A Hawksley random zero sphygmomanometer was used.
ABCD	Determined at two separate visits.
ACCORD	Average of three measurements in the sitting position, using an automated device (Omron 907), after five minutes rest.
ACTIVE I	Measured in duplicated in five minute intervals.
ADVANCE	Mean of two measurements in the sitting position, taken using a standardized automated sphygmomanometer (Omron HEM-705CP,Tokyo, Japan), after the patient was rested for at least five minutes.
ALLHAT	Mean of four measurements, two taken at two separate visits.
ANBP	Mean of four measurements over two visits, taken using a random zero or London School of Hygiene sphygmomanometer in sitting position after 5 minutes of rest.
ANBP2	Mean of measurements at two visits, taken using a mercury sphygmomanometer in the sitting position.
ASCOT-BPLA	Measured three times using a semi-automated device in the sitting position, after five minutes of rest. Mean of last two readings used.
BENEDICT	Mean of three morning measurements.
CAMELOT	Measured using a manual cuff and stethoscope.
CAPPP	Mean of two measurements taken in supine position using conventional mercury sphygmomanometer.
Cardio-Sis	Mean of three consecutive readings at every visit, taken using standard mercury sphygmomanometers after sitting for ten minutes.
CASE-J	Mean of two measurements, taken in the sitting position.
COLM	Mean of two stable measurements that differed by less than 5 mm Hg, taken at least twice at intervals of one to two minutes.
CONVINCE	Not provided.
COPE	Mean of two stable measurements (difference <5mmHg), taken using the upper arm after five minutes rest in a sitting position.
DIABHYCAR	Measured once using a mercury sphygmomanometer in the sitting position.
Dutch TIA	A single measurement in the sitting position using a mercury manometer.
E-COST	Taken in sitting position.
ELSA	Three measurements taken in sitting position using a mercury manometer.
EUROPA	Two measurements taken with a standard sphygmomanometer in sitting position, after five minutes rest.
EWPHE	Taken in sitting position.
HDFP	Four readings taken in sitting position; First and third reading taken using conventional mercury manometer; Second and fourth reading taken using Hawksley random-zero manometer
HIJ-CREATE	Measured using a standard cuff mercury sphygmomanometer in sitting position after five minutes of rest.
HOMED-BP	Clinic: Mean of two measurements taken using a validated sphygmomanometer (OMRON HEM-907IT, Omron Healthcare, Kyoto, Japan) in the sitting position after two minutes rest; Home: as with clinic but taken in the morning, 1 hour after waking up, before breakfast and before taking BP treatment, and mean of readings over 5 days prior to clinic visit.
HOPE	Mean of two measurements in the sitting position after fifteen minutes rest, taken using a mercury sphygmomanometer.
HYVET	Two measurements taken in the standing position, using a mercury sphygmomanometer or a validated automated device.
IDNT	Taken in the sitting position.
INSIGHT	Mean of three measurements after a five minute rest, taken using a mercury sphygmomanometer.
INVEST	Mean of two measurements taken in the sitting position as described in JNC VI.
JMIC-B	Average of two last measurements of three measurements, taken in sitting or supine position, whichever had been decided upon initially.
LIFE	Sitting blood pressure, measured at the trough.
MOSES	Not provided.

Canoy D, *et al. Heart* 2022; 108:1281–1289. doi: 10.1136/heartjnl-2021-320171

NICS-EH	Not provided.
NORDIL	Measured on two separate occasions.
ONTARGET	Average of two readings was used. Each blood pressure reading consisted of measurement using an automated validated device (OMRON, model HEM-757) after 3 minutes of rest while sitting.
PART 2	Average of two measurements using a standard mercury sphygmomanometer following standard protocol.
PEACE	Not provided.
PREVEND IT	Ten consecutive measurements were taken using an automatic Dinamap XL model; last two measurements were averaged to determine BP measurement.
PREVENT	Not provided.
PRoFESS	Average of two measures taken twice, at least two minutes apart, using a standard and validated Omron sphygmomanometer (Omron Healthcare Inc) with an appropriately sized cuff applied to the upper nondominant arm at heart level.[49 88]
PROGRESS	Two measurements of BP were taken using a mercury sphygmomanometer.
SHEP	Average of four seated BP measurements, taken using a Hawksley random zero manometer.
SPRINT	Measured after rest, preferably using an automated device, but a manual device could be used if necessary.
STOP Hypertension-2	After five minutes of rest, BP was measured in the supine position.
SYST-EUR	Average of six sitting BP measurements and six standing BP measurements, two in each position at each of three baseline visits taken one month apart.
TRANSCEND	Average of two readings was used. Each blood pressure reading consisted of measurement using an automated validated device (OMRON, model HEM-757) after 3 minutes of rest while sitting.
UKPDS	Mean of last three of four BP measurements taken after 5 minutes of rest while sitting at consecutive clinic visits using an automated validated device (Copal UA-251 or a Takeda UA-751).
VALISH	Blood pressure taken in sitting position at two separate visits within 2 to 4 weeks.
VALUE	Measured after sitting for five minutes.
VHAS	Lowest value of three measurements, taken in the sitting position after a 10-minute rest, at consecutive one-minute intervals.

Acronyms are described in full in the Trial acronym legend in the Supplement; BP – blood pressure.

Canoy D, *et al. Heart* 2022; 108:1281–1289. doi: 10.1136/heartjnl-2021-320171

Table S8. **Supplementary data for Figure 1 and online Figure S2, showing estimated mean blood pressure separately for each comparison arm at specific time points during follow-up, by trial design.**

Comparison arm	Follow-up period (months)												
	Baseline	3	6	9	12	18	24	30	36	42	48	54	60
**Blood pressure-lowering intensity trials**													
Systolic blood pressure (mmHg)													
More intense	151	141	138	135	133	130	127	126	126	126	127	128	130
Less intense	151	143	142	141	140	138	137	137	136	136	137	137	138
Diastolic blood pressure (mmHg)													
More intense	88	82	80	79	77	75	74	73	73	72	72	73	73
Less intense	88	83	82	82	81	80	79	79	78	78	78	77	77
**Placebo-controlled trials**													
Systolic blood pressure (mmHg)													
Active	146	140	138	137	136	135	134	134	134	135	136	136	137
Placebo	146	143	143	142	141	141	140	140	140	140	140	141	140
Diastolic blood pressure (mmHg)													
Active	83	80	79	79	78	78	77	77	77	77	77	77	77
Placebo	83	82	82	81	81	80	80	80	79	79	79	79	79
**Blood pressure difference trials**													
Systolic blood pressure (mmHg)													
Active	145	140	138	136	135	133	132	132	132	132	133	135	136
Control	145	143	142	141	141	140	139	139	139	140	140	140	140
Diastolic blood pressure (mmHg)													
Active	84	81	80	79	77	77	76	76	76	76	76	76	76
Control	83	82	82	81	81	80	80	80	79	79	79	79	79
**Drug class comparison trials**													
Systolic blood pressure (mmHg)													
Active	156	150	148	146	144	141	139	139	138	139	139	140	140
Control	155	149	147	145	144	141	140	139	139	139	139	140	140
Diastolic blood pressure (mmHg)													
Active	90	86	85	84	83	81	80	80	79	79	79	79	79
Control	89	86	85	84	83	81	80	80	79	79	79	79	79

Estimates based on separate models for each comparison arm, with random intercepts at individual and trial levels, a random slope for time at the individual level (see Method for details) and adjusted for baseline blood pressure, age and sex; Data concatenated at to five years of follow-up.

Canoy D, *et al. Heart* 2022; 108:1281–1289. doi: 10.1136/heartjnl-2021-320171

Table S9. **Supplementary data for Figure 2 and online Figure S3 showing mean blood pressure difference between comparison groups over follow-up time.**

Follow-up period, months	Systolic blood pressure (mmHg) difference (95% CI)	Diastolic blood pressure (mmHg) difference (95% CI)
Blood pressure-lowering intensity trials (9 trials)		
0 to 6	-4.2 (-4.4 to -4.0)	-2.0 (-2.2 to -1.9)
6 to 12	-10.1 (-10.4 to -9.7)	-5.2 (-5.4 to -5.0)
12 to 24	-11.3 (-11.6 to -11.0)	-5.8 (-6.0 to -5.6)
24 to 36	-11.9 (-12.2 to -11.6)	-6.1 (-6.3 to -5.9)
36 to 48	-12.0 (-12.3 to -11.6)	-6.0 (-6.2 to -5.7)
48 to 60	-10.9 (-11.4 to -10.4)	-5.6 (-5.9 to -5.3)
Placebo-controlled trials (21 trials)		
0 to 6	-3.0 (-3.1 to -2.9)	-1.5 (-1.6 to -1.4)
6 to 12	-5.6 (-5.8 to -5.4)	-2.7 (-2.8 to -2.5)
12 to 24	-5.3 (-5.5 to -5.1)	-2.5 (-2.6 to -2.4)
24 to 36	-5.3 (-5.5 to -5.0)	-2.4 (-2.5 to -2.3)
36 to 48	-4.9 (-5.2 to -4.7)	-2.2 (-2.3 to -2.0)
48 to 60	-4.5 (-4.8 to -4.2)	-1.9 (-2.1 to -1.8)
All blood pressure difference trials (30 trials)		
0 to 6	-3.3 (-3.4 to -3.2)	-1.6 (-1.7 to -1.6)
6 to 12	-6.5 (-6.7 to -6.4)	-3.2 (-3.3 to -3.1)
12 to 24	-6.8 (-7.0 to -6.6)	-3.3 (-3.4 to -3.2)
24 to 36	-7.0 (-7.2 to -6.8)	-3.4 (-3.5 to -3.3)
36 to 48	-6.7 (-6.9 to -6.5)	-3.1 (-3.3 to -3.0)
48 to 60	-6.2 (-6.5 to -6.0)	-2.9 (-3.1 to -2.8)
Drug class comparison trials (29 trials)		
0 to 6	-1.0 (-1.1 to -1.0)	-0.4 (-0.4 to -0.3)
6 to 12	-1.9 (-2.1 to -1.8)	-0.7 (-0.8 to -0.6)
12 to 24	-1.6 (-1.8 to -1.5)	-0.8 (-0.8 to -0.7)
24 to 36	-1.5 (-1.6 to -1.3)	-0.7 (-0.7 to -0.6)
36 to 48	-1.5 (-1.7 to -1.4)	-0.8 (-0.9 to -0.7)
48 to 60	-1.6 (-1.8 to -1.5)	-1.0 (-1.1 to -0.9)

Model based on fixed treatment effect (see Method), and estimates adjusted for baseline blood pressure, age and sex; Negative values indicate lower blood pressure in the active than in the control group.

Table S10. **Supplementary data for Figure 2 and online Figure S3 showing achieved mean blood pressure difference between active and control groups with and without taking into account early follow-up measurements.**

Follow-up period, months	Systolic blood pressure (mmHg) difference (95% CI)	Diastolic blood pressure (mmHg) difference (95% CI)
Blood pressure-lowering intensity trials (9 trials)		
All follow-up	-7.6 (-7.7 to -7.4)	-3.7 (-3.8 to -3.6)
From 12 months to end of follow-up	-11.1 (-11.3 to -10.8)	-5.6 (-5.7 to -5.4)
Placebo-controlled trials (20 trials)		
All follow-up	-4.0 (-4.1 to -3.9)	-1.9 (-2.0 to -1.8)
From 12 months to end of follow-up	-5.1 (-5.3 to -5.0)	-2.3 (-2.4 to -2.2)
Blood pressure difference trials (30 trials)		
All follow-up	-4.8 (-4.9 to -4.7)	-2.3 (-2.4 to -2.3)
From 12 months to end of follow-up	-6.6 (-6.7 to -6.5)	-3.2 (-3.2 to -3.1)
Drug class comparison trials (29 trials)		
All follow-up	-1.4 (-1.5 to -1.3)	-0.5 (-0.6 to -0.5)
From 12 months to end of follow-up	-1.4 (-1.5 to -1.3)	-0.6 (-0.7 to -0.6)

Model based on fixed treatment effect and random intercept for trials (see Method for details) and adjusted for baseline blood pressure, age and sex; Negative values indicate lower blood pressure in the active than in the control group.

Canoy D, *et al. Heart* 2022; 108:1281–1289. doi: 10.1136/heartjnl-2021-320171

Table S11. **Achieved mean blood pressure difference* between active and control groups stratified by time period of conducting the trial.**

Year of end of study	Systolic blood pressure (mmHg) difference (95% CI)	Diastolic blood pressure (mmHg) difference (95% CI)
Blood pressure-lowering intensity trials (9 trials)		
Pre-2000 (3 trials)	-10.1 (-10.5 to -9.6)	-5.3 (-5.5 to -5.0)
2000 or later (6 trials)	-11.4 (-11.6 to -11.1)	-5.6 (-5.8 to -5.4)
Placebo-controlled trials (21 trials)		
Pre-2000 (9 trials)	-7.7 (-8.0 to -7.4)	-3.3 (-3.5 to -3.1)
2000 or later (12 trials)	-4.5 (-4.6 to -4.3)	-2.1 (-2.2 to -2.0)
Blood pressure difference trials (30 trials)		
Pre-2000 (12 trials)	-7.8 (-8.1 to -7.5)	-3.5 (-3.7 to -3.3)
2000 or later (18 trials)	-5.9 (-6.1 to -5.8)	-2.9 (-2.9 to -2.8)
Drug class comparison trials (29 trials)		
Pre-2000 (7 trials)	-1.1 (-1.3 to -0.8)	-0.8 (-0.9 to -0.6)
2000 or later (22 trials)	-1.4 (-1.5 to -1.3)	-0.6 (-0.6 to -0.5)

Model based on fixed treatment effect and random intercept for trials (see Method for details) and adjusted for baseline blood pressure, age and sex; Negative values indicate lower blood pressure in the active than in the control group; *Based on blood pressure measurements from ≥12 months to end of follow-up.

Table S12. Comparison of models estimating mean blood pressure difference between comparison arms.

Model	SBP (mmHg)		DBP (mmHg)	
	Mean difference (95% CI)	AIC	Mean difference (95% CI)	AIC
**BLOOD PRESSURE-LOWERING INTENSITY TRIALS**				
All follow-up				
Model 1: Fixed effect (treatment and time) and random intercept (trial)	-9.7 (-9.8 to -9.6)	3,708,049	-5.0 (-5.1 to -4.9)	3,254,826
Model 2: Random intercepts (trial and individual levels), fixed treatment effect, fixed time effect (cubic term) and random slope for time (individual level)	-7.6 (-7.7 to -7.4)	3,598,296	-3.7 (-3.8 to -3.6)	3,126,973
From 12 months to end of follow-up				
Model 1: Fixed effect (treatment and time) and random intercept (trial)	-11.8 (-11.9 to -11.7)	2,431,042	-6.1 (-6.1 to -6.0)	2,150,542
Model 2: Random intercepts (trial and individual levels), fixed treatment effect, fixed time effect (cubic term) and random slope for time (individual level)	-11.1 (-11.3 to -10.8)	2,359,772	-5.6 (-5.7 to -5.4)	2,059,717
**PLACEBO-CONTROLLED TRIALS**				
All follow-up				
Model 1: Fixed effect (treatment and time) and random intercept (trial)	-4.6 (-4.6 to -4.5)	7,326,530	-2.1 (-2.1 to -2.1)	6,313,968
Model 2: Random intercepts (trial and individual levels), fixed treatment effect, fixed time effect (cubic term) and random slope for time (individual level)	-4.0 (-4.1 to -3.9)	7,101,069	-1.9 (-2.0 to -1.8)	6,114,559
From 12 months to end of follow-up				
Model 1: Fixed effect (treatment and time) and random intercept (trial)	-5.1 (-5.2 to -5.0)	4,224,464	-2.3 (-2.4 to -2.3)	3,642,401
Model 2: Random intercepts (trial and individual levels), fixed treatment effect, fixed time effect (cubic term) and random slope for time (individual level)	-5.1 (-5.3 to -5.0)	4,082,606	-2.3 (-2.4 to -2.2)	3,515,852
**ALL BLOOD PRESSURE DIFFERENCE TRIALS**				
All follow-up				
Model 1: Fixed effect (treatment and time) and random intercept (trial)	-6.0 (-6.1 to -6.0)	12,216,192	-3.0 (-3.0 to -2.9)	10,594,707
Model 2: Random intercepts (trial and individual levels), fixed treatment effect, fixed time effect (cubic term) and random slope for time (individual level)	-4.8 (-4.9 to -4.7)	11,826,496	-2.3 (-2.4 to -2.3)	10,216,192
From 12 months to end of follow-up				
Model 1: Fixed effect (treatment and time) and random intercept (trial)	-7.3 (-7.4 to -7.3)	7,117,093	-3.6 (-3.6 to -3.5)	6,186,156
Model 2: Random intercepts (trial and individual levels), fixed treatment effect, fixed time effect (cubic term) and random slope for time (individual level)	-6.6 (-6.7 to -6.5)	6,854,402	-3.2 (-3.2 to -3.1)	5,926,777
**DRUG COMPARISON TRIALS**				
All follow-up				
Model 1: Fixed effect (treatment and time) and random intercept (trial)	-1.4 (-1.4 to -1.3)	16,539,815	-0.6 (-0.6 to -0.6)	14,228,432
Model 2: Random intercepts (trial and individual levels), fixed treatment effect, fixed time effect (cubic term) and random slope for time (individual level)	-1.4 (-1.5 to -1.3)	16,078,396	-0.5 (-0.6 to -0.5)	13,790,959
From 12 months to end of follow-up				
Model 1: Fixed effect (treatment and time) and random intercept (trial)	-1.3 (-1.4 to -1.3)	10,516,160	-0.7 (-0.7 to -0.6)	9,069,610
Model 2: Random intercepts (trial and individual levels), fixed treatment effect, fixed time effect (cubic term) and random slope for time (individual level)	-1.4 (-1.5 to -1.3)	10,193,302	-0.6 (-0.7 to -0.6)	8,751,146

SBP – systolic blood pressure; DBP – diastolic blood pressure; AIC: Aikeke information criterion; Adjusted for baseline blood pressure, age and sex; Negative values indicate lower blood pressure in the active than in the control group.

Canoy D, *et al. Heart* 2022; 108:1281–1289. doi: 10.1136/heartjnl-2021-320171

CLINICAL PERSPECTIVES

Antihypertensive medications are effective in lowering blood pressure in people with a wide range of characteristics. If clinically indicated, such treatment should be considered in men and women, in relatively young and older adults, with or without a history of cardiovascular disease or diabetes, and even among those whose blood pressure levels are below the usual thresholds for defining hypertension.

Treatment strategies should consider aiming for a substantive reduction in blood pressure. Whilst setting a blood pressure target is practical, such an approach could either mean a modest reduction for some and difficult to achieve for others, depending on their baseline or usual pre-treatment blood pressure levels. The optimal treatment strategy would focus on achieving the greatest long-term blood pressure reduction possible that can be tolerated by the patient and is feasible to implement.

Monitoring of blood pressure of an individual patient is necessary to assess treatment response. However, short-term measurements, such as readings taken a few months since treatment initiation, are unlikely to be informative of the extent to which the blood pressure has been reduced. Moreover, achieved reduction in blood pressure attenuates over time, which is likely due to reduced treatment compliance. Hence, long-term monitoring of blood pressure and addressing issues on treatment adherence should form part of clinical care to maximise the benefits of pharmacologic treatment of raised blood pressure. Longitudinal data of blood pressure reduction reported in this study can also provide some guidance on achievable blood pressure reductions based on trialled regimens.

Canoy D, *et al. Heart* 2022; 108:1281–1289. doi: 10.1136/heartjnl-2021-320171

TRIAL ACRONYM LEGEND

Trial acronym	Full name or description
**AASK**	African American Study of Kidney Disease and Hypertension
**ABCD**	Appropriate Blood Pressure Control in Diabetes
**ACCORD**	Action to Control Cardiovascular Risk in Diabetes blood pressure trial
**ACTIVE I**	Atrial Fibrillation Clopidogrel Trial with Irbesartan for Prevention of Vascular Events
**ADVANCE**	Action in Diabetes and Vascular disease: preterAx and diamicroN-MR Controlled Evaluation
**ALLHAT**	Antihypertensive and Lipid Lowering Treatment to Prevent Heart Attack Trial
**ANBP**	Australian National Blood Pressure Study
**ANBP2**	Second Australian National Blood Pressure Study
**ASCOT-BPLA**	Anglo-Scandinavian Cardiac Outcomes Trial-Blood Pressure Lowering Arm
**BENEDICT**	BErgamo NEphrologic DIabetes Complications Trial
**CAMELOT**	Comparison of Amlodipine vs Enalapril to Limit Occurrences of Thrombosis
**CAPPP**	Captopril Prevention Project
**Cardio-Sis**	Studio Italiano Sugli Effetti Cardiovascolari del Controllo della Pressione Arteriosa Sistolica
**CASE-J**	Candesartan Antihypertensive Survival Evaluation in Japan Trial
**COLM**	Combination of OLMesartan study
**CONVINCE**	Controlled ONset Verapamil INvestigation of Cardiovascular Endpoints trial
**COPE**	Combination Therapy of Hypertension to Prevent Cardiovascular Events
**DIABHYCAR**	Noninsulin-dependent diabetes, hypertension, microalbuminuria or proteinuria, cardiovascular events, and ramipril
**Dutch TIA Trial**	Dutch Transient Ischemic Attack Trial
**E-COST**	Efficacy of Candesartan on Outcome in Saitama Trial
**ELSA**	European Lacidipine Study on Atherosclerosis
**EUROPA**	EUropean trial on Reduction Of cardiac events with Perindopril in patients with stable coronary Artery disease
**EWPHE**	European Working Party on High Blood Pressure in the Elderly
**HDFP**	Hypertension Detection and Follow-up Program
**HIJ-CREATE**	Heart Institute of Japan Candesartan Randomized Trial for Evaluation in Coronary Artery Disease
**HOMED-BP**	Hypertension Objective Treatment Based on Measurement by Electrical Devices of Blood Pressure
**HOPE**	Heart Outcomes Prevention Evaluation
**HYVET**	Hypertension in the Very Elderly Trial
**IDNT**	Irbesartan Diabetic Nephropathy Trial
**INSIGHT**	International Nifedipine GITS study: Intervention as a Goal in Hypertension Treatment
**INVEST**	International Verapamil-Trandolapril Study
**JMIC-B**	Japan Multicenter Investigation for Cardiovascular Diseases-B
**LIFE**	Losartan Intervention For Endpoint reduction
**MOSES**	Morbidity and Mortality After Stroke, Eprosartan Compared With Nitrendipine for Secondary Prevention
**NICS-EH**	National Intervention Cooperative Study in Elderly Hypertensives
**NORDIL**	Nordic Diltiazem Study
**ONTARGET**	Ongoing Telmisartan Alone and in Combination with Ramipril Global Endpoint Trial
**PART 2**	Prevention of Atherosclerosis with Ramipril Trial
**PEACE**	Prevention of Events with Angiotensin Converting Enzyme Inhibition
**PREVEND IT**	Prevention of Renal and Vascular Endstage Disease Intervention Trial
**PREVENT**	Prospective Randomized Evaluation of the Vascular Effects of Norvasc Trial
**PRoFESS**	Prevention Regimen for Effectively Avoiding Second Strokes
**PROGRESS**	Perindopril Protection Against Recurrent Stroke Study
**SHEP**	Systolic Hypertension in the Elderly Program
**SPRINT**	Systolic Blood Pressure Intervention Trial
**STOP Hypertension-2**	Swedish Trial in Old Patients with Hypertension-2
**SYST-EUR**	SYSTolic Hypertension in EURope
**TRANSCEND**	Telmisartan Randomized Assessment Study in ACE Intolerant Subjects with Cardiovascular Disease
**UKPDS**	UK Prospective Diabetes Study
**VALISH**	Valsartan in Elderly Isolated Systolic Hypertension
**VALUE**	Valsartan Antihypertensive Long-term Use Evaluation
**VHAS**	Verapamil in Hypertension and Atherosclerosis Study

Canoy D, *et al. Heart* 2022; 108:1281–1289. doi: 10.1136/heartjnl-2021-320171

REFERENCES

1. Lewis EJ, Hunsicker LG, Clarke WR, Berl T, Pohl MA, Lewis JB, Ritz E, Atkins RC, Rohde R, Raz I, et al. Renoprotective effect of the angiotensin-receptor antagonist irbesartan in patients with nephropathy due to type 2 diabetes. *N Engl J Med* 2001;345:851-60. doi: 10.1056/NEJMoa011303
2. Verdecchia P, Staessen JA, Angeli F, de Simone G, Achilli A, Ganau A, Mureddu G, Pede S, Maggioni AP, Lucci D, et al. Usual versus tight control of systolic blood pressure in non-diabetic patients with hypertension (Cardio-Sis): an open-label randomised trial. *Lancet* 2009;374:525-33. doi: 10.1016/S0140-6736(09)61340-4
3. Debray TP, Schuit E, Efthimiou O, Reitsma JB, Ioannidis JP, Salanti G, Moons KG, GetReal W. An overview of methods for network meta-analysis using individual participant data: when do benefits arise? *Stat Methods Med Res* 2018;27:1351-64. doi: 10.1177/0962280216660741
4. Legha A, Riley RD, Ensor J, Snell KIE, Morris TP, Burke DL. Individual participant data meta-analysis of continuous outcomes: A comparison of approaches for specifying and estimating one-stage models. *Stat Med* 2018;37:4404-20. doi: 10.1002/sim.7930
5. Higgins JP, Whitehead A, Turner RM, Omar RZ, Thompson SG. Meta-analysis of continuous outcome data from individual patients. *Stat Med* 2001;20:2219-41. doi: 10.1002/sim.918
6. Thompson SG, Sharp SJ. Explaining heterogeneity in meta-analysis: a comparison of methods. *Stat Med* 1999;18:2693-708. doi: 10.1002/(sici)1097-0258(19991030)18:20<2693::aid-sim235>3.0.co;2-v
7. Ahmed I, Sutton AJ, Riley RD. Assessment of publication bias, selection bias, and unavailable data in meta-analyses using individual participant data: a database survey. *BMJ* 2012;344:d7762. doi: 10.1136/bmj.d7762
8. Czernichow S, Zanchetti A, Turnbull F, Barzi F, Ninomiya T, Kengne AP, Lambers Heerspink HJ, Perkovic V, Huxley R, Arima H, et al. The effects of blood pressure reduction and of different blood pressure-lowering regimens on major cardiovascular events according to baseline blood pressure: meta-analysis of randomized trials. *J Hypertens* 2011;29:4-16. doi: 10.1097/HJH.0b013e32834000be
9. Blood Pressure Lowering Treatment Trialists' Collaboration, Ninomiya T, Perkovic V, Turnbull F, Neal B, Barzi F, Cass A, Baigent C, Chalmers J, Li N, et al. Blood pressure lowering and major cardiovascular events in people with and without chronic kidney disease: meta-analysis of randomised controlled trials. *BMJ* 2013;347:f5680. doi: 10.1136/bmj.f5680
10. Thomopoulos C, Parati G, Zanchetti A. Effects of blood pressure lowering on outcome incidence in hypertension: 2. Effects at different baseline and achieved blood pressure levels--overview and meta-analyses of randomized trials. *J Hypertens* 2014;32:2296-304. doi: 10.1097/HJH.0000000000000379
11. Wang JG, Staessen JA, Franklin SS, Fagard R, Gueyffier F. Systolic and diastolic blood pressure lowering as determinants of cardiovascular outcome. *Hypertension* 2005;45:907-13. doi: 10.1161/01.HYP.0000165020.14745.79
12. Wright JT, Jr., Bakris G, Greene T, Agodoa LY, Appel LJ, Charleston J, Cheek D, Douglas-Baltimore JG, Gassman J, Glassock R, et al. Effect of blood pressure lowering and antihypertensive drug class on progression of hypertensive kidney disease: results from the AASK trial. *JAMA* 2002;288:2421-31. doi: 10.1001/jama.288.19.2421
13. Gassman JJ, Greene T, Wright JT, Jr., Agodoa L, Bakris G, Beck GJ, Douglas J, Jamerson K, Lewis J, Kutner M, et al. Design and statistical aspects of the African American Study of Kidney Disease and Hypertension (AASK). *J Am Soc Nephrol* 2003;14:S154-65. doi: 10.1097/01.asn.0000070080.21680.cb
14. Estacio RO, Savage S, Nagel NJ, Schrier RW. Baseline characteristics of participants in the Appropriate Blood Pressure Control in Diabetes trial. *Control Clin Trials* 1996;17:242-57. doi: 10.1016/0197-2456(95)00154-9
15. Estacio RO, Jeffers BW, Hiatt WR, Biggerstaff SL, Gifford N, Schrier RW. The effect of nisoldipine as compared with enalapril on cardiovascular outcomes in patients with non-insulin-dependent diabetes and hypertension. *N Engl J Med* 1998;338:645-52. doi: 10.1056/NEJM199803053381003
16. Schrier RW, Estacio RO, Esler A, Mehler P. Effects of aggressive blood pressure control in normotensive type 2 diabetic patients on albuminuria, retinopathy and strokes. *Kidney Int* 2002;61:1086-97. doi: 10.1046/j.1523-1755.2002.00213.x
17. ACCORD Study Group, Cushman WC, Evans GW, Byington RP, Goff DC, Jr., Grimm RH, Jr., Cutler JA, Simons-Morton DG, Basile JN, Corson MA, et al. Effects of intensive blood-pressure control in type 2 diabetes mellitus. *N Engl J Med* 2010;362:1575-85. doi: 10.1056/NEJMoa1001286
18. Cardio-Sis Study Group. Randomized study of traditional versus aggressive systolic blood pressure control (Cardio-Sis): rationale, design and characteristics of the study population. *J Hum Hypertens* 2008;22:243-51. doi: 10.1038/sj.jhh.1002313
19. Five-year findings of the hypertension detection and follow-up program. I. Reduction in mortality of persons with high blood pressure, including mild hypertension. Hypertension Detection and Follow-up Program Cooperative Group. *JAMA* 1979;242:2562-71.
20. Asayama K, Ohkubo T, Metoki H, Obara T, Inoue R, Kikuya M, Thijs L, Staessen JA, Imai Y, Hypertension Objective Treatment Based on Measurement by Electrical Devices of Blood P. Cardiovascular outcomes in the first trial of antihypertensive therapy guided by self-measured home blood pressure. *Hypertens Res* 2012;35:1102-10. doi: 10.1038/hr.2012.125

21. SPRINT Research Group, Wright JT, Jr., Williamson JD, Whelton PK, Snyder JK, Sink KM, Rocco MV, Reboussin DM, Rahman M, Oparil S, et al. A Randomized Trial of Intensive versus Standard Blood-Pressure Control. *N Engl J Med* 2015;373:2103-16. doi: 10.1056/NEJMoa1511939
22. Hypertension in Diabetes Study Group. Hypertension in Diabetes Study. III. Prospective study of therapy of hypertension in type 2 diabetic patients: efficacy of ACE inhibition and beta-blockade. *Diabet Med* 1994;11:773-82.
23. UK Prospective Diabetes Study Group. Tight blood pressure control and risk of macrovascular and microvascular complications in type 2 diabetes: UKPDS 38. *BMJ* 1998;317:703-13.
24. UK Prospective Diabetes Study Group. Efficacy of atenolol and captopril in reducing risk of macrovascular and microvascular complications in type 2 diabetes: UKPDS 39. . *BMJ* 1998;317:713-20.
25. Ogihara T, Saruta T, Matsuoka H, Shimamoto K, Fujita T, Shimada K, Imai Y, Nishigaki M. Valsartan in elderly isolated systolic hypertension (VALISH) study: rationale and design. *Hypertens Res* 2004;27:657-61. doi: 10.1291/hypres.27.657
26. Ogihara T, Saruta T, Rakugi H, Matsuoka H, Shimamoto K, Shimada K, Imai Y, Kikuchi K, Ito S, Eto T, et al. Target blood pressure for treatment of isolated systolic hypertension in the elderly: valsartan in elderly isolated systolic hypertension study. *Hypertension* 2010;56:196-202. doi: 10.1161/HYPERTENSIONAHA.109.146035
27. ACTIVE I Investigators, Yusuf S, Healey JS, Pogue J, Chrolavicius S, Flather M, Hart RG, Hohnloser SH, Joyner CD, Pfeffer MA, et al. Irbesartan in patients with atrial fibrillation. *N Engl J Med* 2011;364:928-38. doi: 10.1056/NEJMoa1008816
28. Patel A, ADVANCE Collaborative Group, MacMahon S, Chalmers J, Neal B, Woodward M, Billot L, Harrap S, Poulter N, Marre M, et al. Effects of a fixed combination of perindopril and indapamide on macrovascular and microvascular outcomes in patients with type 2 diabetes mellitus (the ADVANCE trial): a randomised controlled trial. *Lancet* 2007;370:829-40. doi: 10.1016/S0140-6736(07)61303-8
29. The Australian therapeutic trial in mild hypertension. Report by the Management Committee. *Lancet* 1980;1:1261-7.
30. Doyle AE. The Australian National Blood Pressure Study. *Trends Pharmacol Sci* 1981;2:293-96.
31. BENEDICT Group. The BErgamo NEphrologic DIabetes Complications Trial (BENEDICT): design and baseline characteristics. *Control Clin Trials* 2003;24:442-61. doi: 10.1016/s0197-2456(03)00028-x
32. Ruggenenti P, Fassi A, Ilieva AP, Bruno S, Iliev IP, Brusegan V, Rubis N, Gherardi G, Arnoldi F, Ganeva M, et al. Preventing microalbuminuria in type 2 diabetes. *N Engl J Med* 2004;351:1941-51. doi: 10.1056/NEJMoa042167
33. Nissen SE, Tuzcu EM, Libby P, Thompson PD, Ghali M, Garza D, Berman L, Shi H, Buebendorf E, Topol EJ, et al. Effect of antihypertensive agents on cardiovascular events in patients with coronary disease and normal blood pressure: the CAMELOT study: a randomized controlled trial. *JAMA* 2004;292:2217-25. doi: 10.1001/jama.292.18.2217
34. Lievre M, Marre M, Chatellier G, Plouin P, Reglier J, Richardson L, Bugnard F, Vasmant D. The non-insulin-dependent diabetes, hypertension, microalbuminuria or proteinuria, cardiovascular events, and ramipril (DIABHYCAR) study: design, organization, and patient recruitment. DIABHYCAR Study Group. *Control Clin Trials* 2000;21:383-96. doi: 10.1016/s0197-2456(00)00060-x
35. Marre M, Lievre M, Chatellier G, Mann JF, Passa P, Menard J, Investigators DS. Effects of low dose ramipril on cardiovascular and renal outcomes in patients with type 2 diabetes and raised excretion of urinary albumin: randomised, double blind, placebo controlled trial (the DIABHYCAR study). *BMJ* 2004;328:495. doi: 10.1136/bmj.37970.629537.0D
36. Dutch TIA Trial Study Group. Trial of secondary prevention with atenolol after transient ischemic attack or nondisabling ischemic stroke. . *Stroke* 1993;24:543-8. doi: 10.1161/01.str.24.4.543
37. Gomma AH, Fox KM. The EUROPA trial: design, baseline demography and status of the substudies. *Cardiovasc Drugs Ther* 2001;15:169-79. doi: 10.1023/a:1011131130922
38. Fox KM, EURopean trial On reduction of cardiac events with Perindopril in stable coronary Artery disease Investigators. Efficacy of perindopril in reduction of cardiovascular events among patients with stable coronary artery disease: randomised, double-blind, placebo-controlled, multicentre trial (the EUROPA study). *Lancet* 2003;362:782-8. doi: 10.1016/s0140-6736(03)14286-9
39. An international trial of antihypertensive therapy in elderly patients. Objectives, protocol and organization. European Working Party on High Blood Pressure in the Elderly (EWPHE). *Arch Int Pharmacodyn Ther* 1985;275:300-34.
40. Amery A, Birkenhager W, Brixko P, Bulpitt C, Clement D, Deruyttere M, De Schaepdryver A, Dollery C, Fagard R, Forette F, et al. Mortality and morbidity results from the European Working Party on High Blood Pressure in the Elderly trial. *Lancet* 1985;1:1349-54. doi: 10.1016/s0140-6736(85)91783-0
41. Heart Outcomes Prevention Evaluation Study Investigators, Yusuf S, Sleight P, Pogue J, Bosch J, Davies R, Dagenais G. Effects of an angiotensin-converting-enzyme inhibitor, ramipril, on cardiovascular events in high-risk patients. *N Engl J Med* 2000;342:145-53. doi: 10.1056/NEJM200001203420301
42. Beckett NS, Peters R, Fletcher AE, Staessen JA, Liu L, Dumitrascu D, Stoyanovsky V, Antikainen RL, Nikitin Y, Anderson C, et al. Treatment of hypertension in patients 80 years of age or older. *N Engl J Med* 2008;358:1887-98. doi: 10.1056/NEJMoa0801369

43. MacMahon S, Sharpe N, Gamble G, Clague A, Mhurchu CN, Clark T, Hart H, Scott J, White H. Randomized, placebo-controlled trial of the angiotensin-converting enzyme inhibitor, ramipril, in patients with coronary or other occlusive arterial disease. PART-2 Collaborative Research Group. Prevention of Atherosclerosis with Ramipril. *J Am Coll Cardiol* 2000;36:438-43. doi: 10.1016/s0735-1097(00)00736-1
44. Braunwald E, Domanski MJ, Fowler SE, Geller NL, Gersh BJ, Hsia J, Pfeffer MA, Rice MM, Rosenberg YD, Rouleau JL, et al. Angiotensin-converting-enzyme inhibition in stable coronary artery disease. *N Engl J Med* 2004;351:2058-68. doi: 10.1056/NEJMoa042739
45. Asselbergs FW, Diercks GF, Hillege HL, van Boven AJ, Janssen WM, Voors AA, de Zeeuw D, de Jong PE, van Veldhuisen DJ, van Gilst WH, et al. Effects of fosinopril and pravastatin on cardiovascular events in subjects with microalbuminuria. *Circulation* 2004;110:2809-16. doi: 10.1161/01.CIR.0000146378.65439.7A
46. Byington RP, Miller ME, Herrington D, Riley W, Pitt B, Furberg CD, Hunninghake DB, Mancini GB. Rationale, design, and baseline characteristics of the Prospective Randomized Evaluation of the Vascular Effects of Norvasc Trial (PREVENT). *Am J Cardiol* 1997;80:1087-90. doi: 10.1016/s0002-9149(97)00611-5
47. Pitt B, Byington RP, Furberg CD, Hunninghake DB, Mancini GB, Miller ME, Riley W. Effect of amlodipine on the progression of atherosclerosis and the occurrence of clinical events. PREVENT Investigators. *Circulation* 2000;102:1503-10. doi: 10.1161/01.cir.102.13.1503
48. Yusuf S, Diener HC, Sacco RL, Cotton D, Ounpuu S, Lawton WA, Palesch Y, Martin RH, Albers GW, Bath P, et al. Telmisartan to prevent recurrent stroke and cardiovascular events. *N Engl J Med* 2008;359:1225-37. doi: 10.1056/NEJMoa0804593
49. Diener HC, Sacco R, Yusuf S, Steering Committee, PRoFESS Study Group. Rationale, design and baseline data of a randomized, double-blind, controlled trial comparing two antithrombotic regimens (a fixed-dose combination of extended-release dipyridamole plus ASA with clopidogrel) and telmisartan versus placebo in patients with strokes: the Prevention Regimen for Effectively Avoiding Second Strokes Trial (PRoFESS). *Cerebrovasc Dis* 2007;23:368-80. doi: 10.1159/000100105
50. PROGRESS Management Committee. PROGRESS - Perindopril Protection Against Recurrent Stroke Study: characteristics of the study population at baseline. Progress Management Committee. *J Hypertens* 1999;17:1647-55.
51. PROGRESS Collaborative Group. Randomised trial of a perindopril-based blood-pressure-lowering regimen among 6,105 individuals with previous stroke or transient ischaemic attack. *Lancet* 2001;358:1033-41. doi: 10.1016/S0140-6736(01)06178-5
52. SHEP Cooperative Research Group. Prevention of stroke by antihypertensive drug treatment in older persons with isolated systolic hypertension. Final results of the Systolic Hypertension in the Elderly Program (SHEP). *JAMA* 1991;265:3255-64.
53. Staessen JA, Fagard R, Thijs L, Celis H, Arabidze GG, Birkenhager WH, Bulpitt CJ, de Leeuw PW, Dollery CT, Fletcher AE, et al. Randomised double-blind comparison of placebo and active treatment for older patients with isolated systolic hypertension. The Systolic Hypertension in Europe (Syst-Eur) Trial Investigators. *Lancet* 1997;350:757-64. doi: 10.1016/s0140-6736(97)05381-6
54. Teo K, Yusuf S, Sleight P, Anderson C, Mookadam F, Ramos B, Hilbrich L, Pogue J, Schumacher H, Ontarget/Transcend Investigators. Rationale, design, and baseline characteristics of 2 large, simple, randomized trials evaluating telmisartan, ramipril, and their combination in high-risk patients: the Ongoing Telmisartan Alone and in Combination with Ramipril Global Endpoint Trial/Telmisartan Randomized Assessment Study in ACE Intolerant Subjects with Cardiovascular Disease (ONTARGET/TRANSCEND) trials. *Am Heart J* 2004;148:52-61. doi: 10.1016/j.ahj.2004.03.020
55. Telmisartan Randomised AssessmeNt Study in ACE iNtolerant subjects with cardiovascular Disease (TRANSCEND) Investigators, Yusuf S, Teo K, Anderson C, Pogue J, Dyal L, Copland I, Schumacher H, Dagenais G, Sleight P. Effects of the angiotensin-receptor blocker telmisartan on cardiovascular events in high-risk patients intolerant to angiotensin-converting enzyme inhibitors: a randomised controlled trial. *Lancet* 2008;372:1174-83. doi: 10.1016/S0140-6736(08)61242-8
56. Davis BR, Cutler JA, Gordon DJ, Furberg CD, Wright JT, Jr., Cushman WC, Grimm RH, LaRosa J, Whelton PK, Perry HM, et al. Rationale and design for the Antihypertensive and Lipid Lowering Treatment to Prevent Heart Attack Trial (ALLHAT). ALLHAT Research Group. *Am J Hypertens* 1996;9:342-60. doi: 10.1016/0895-7061(96)00037-4
57. ALLHAT Officers and Coordinators for the ALLHAT Collaborative Research Group. The Antihypertensive Lipid-Lowering Treatment to Prevent Heart Attack Trial. Major outcomes in high-risk hypertensive patients randomized to angiotensin-converting enzyme inhibitor or calcium channel blocker vs diuretic: The Antihypertensive and Lipid-Lowering Treatment to Prevent Heart Attack Trial (ALLHAT). *JAMA* 2002;288:2981-97. doi: 10.1001/jama.288.23.2981
58. Wing LM, Reid CM, Ryan P, Beilin LJ, Brown MA, Jennings GL, Johnston CI, McNeil JJ, Macdonald GJ, Marley JE, et al. A comparison of outcomes with angiotensin-converting--enzyme inhibitors and diuretics for hypertension in the elderly. *N Engl J Med* 2003;348:583-92. doi: 10.1056/NEJMoa021716

59. Sever PS, Dahlof B, Poulter NR, Wedel H, Beevers G, Caulfield M, Collins R, Kjeldsen SE, McInnes GT, Mehlsen J, et al. Rationale, design, methods and baseline demography of participants of the Anglo-Scandinavian Cardiac Outcomes Trial. ASCOT investigators. *J Hypertens* 2001;19:1139-47. doi: 10.1097/00004872-200106000-00020
60. Dahlof B, Sever PS, Poulter NR, Wedel H, Beevers DG, Caulfield M, Collins R, Kjeldsen SE, Kristinsson A, McInnes GT, et al. Prevention of cardiovascular events with an antihypertensive regimen of amlodipine adding perindopril as required versus atenolol adding bendroflumethiazide as required, in the Anglo-Scandinavian Cardiac Outcomes Trial-Blood Pressure Lowering Arm (ASCOT-BPLA): a multicentre randomised controlled trial. *Lancet* 2005;366:895-906. doi: 10.1016/S0140-6736(05)67185-1
61. The Captopril Prevention Project: a prospective intervention trial of angiotensin converting enzyme inhibition in the treatment of hypertension. The CAPPP Group. *J Hypertens* 1990;8:985-90. doi: 10.1097/00004872-199011000-00002
62. Hansson L, Lindholm LH, Niskanen L, Lanke J, Hedner T, Niklason A, Luomanmaki K, Dahlof B, de Faire U, Morlin C, et al. Effect of angiotensin-converting-enzyme inhibition compared with conventional therapy on cardiovascular morbidity and mortality in hypertension: the Captopril Prevention Project (CAPPP) randomised trial. *Lancet* 1999;353:611-6. doi: 10.1016/s0140-6736(98)05012-0
63. Ogihara T, Nakao K, Fukui T, Fukiyama K, Ueshima K, Oba K, Sato T, Saruta T, Candesartan Antihypertensive Survival Evaluation in Japan Trial Group. Effects of candesartan compared with amlodipine in hypertensive patients with high cardiovascular risks: candesartan antihypertensive survival evaluation in Japan trial. *Hypertension* 2008;51:393-8. doi: 10.1161/HYPERTENSIONAHA.107.098475
64. Fukui T, Rahman M, Hayashi K, Takeda K, Higaki J, Sato T, Fukushima M, Sakamoto J, Morita S, Ogihara T, et al. Candesartan Antihypertensive Survival Evaluation in Japan (CASE-J) trial of cardiovascular events in high-risk hypertensive patients: rationale, design, and methods. *Hypertens Res* 2003;26:979-90. doi: 10.1291/hypres.26.979
65. Ogihara T, Saruta T, Rakugi H, Shimamoto K, Ito S, Matsuoka H, Horiuchi M, Imaizumi T, Takishita S, Higaki J, et al. Rationale, study design and implementation of the COLM study: the combination of OLMesartan and calcium channel blocker or diuretic in high-risk elderly hypertensive patients. *Hypertens Res* 2009;32:163-7. doi: 10.1038/hr.2008.31
66. Ogihara T, Saruta T, Rakugi H, Saito I, Shimamoto K, Matsuoka H, Shimada K, Ito S, Horiuchi M, Imaizumi T, et al. Combinations of olmesartan and a calcium channel blocker or a diuretic in elderly hypertensive patients: a randomized, controlled trial. *J Hypertens* 2014;32:2054-63; discussiom 63. doi: 10.1097/HJH.0000000000000281
67. Black HR, Elliott WJ, Neaton JD, Grandits G, Grambsch P, Grimm RH, Jr., Hansson L, Lacouciere Y, Muller J, Sleight P, et al. Rationale and design for the Controlled ONset Verapamil INvestigation of Cardiovascular Endpoints (CONVINCE) Trial. *Control Clin Trials* 1998;19:370-90. doi: 10.1016/s0197-2456(98)00013-0
68. Black HR, Elliott WJ, Grandits G, Grambsch P, Lucente T, White WB, Neaton JD, Grimm RH, Jr., Hansson L, Lacourciere Y, et al. Principal results of the Controlled Onset Verapamil Investigation of Cardiovascular End Points (CONVINCE) trial. *JAMA* 2003;289:2073-82. doi: 10.1001/jama.289.16.2073
69. Matsuzaki M, Ogihara T, Umemoto S, Rakugi H, Matsuoka H, Shimada K, Abe K, Suzuki N, Eto T, Higaki J, et al. Prevention of cardiovascular events with calcium channel blocker-based combination therapies in patients with hypertension: a randomized controlled trial. *J Hypertens* 2011;29:1649-59. doi: 10.1097/HJH.0b013e328348345d
70. Suzuki H, Kanno Y, Efficacy of Candesartan on Outcome in Saitama Trial G. Effects of candesartan on cardiovascular outcomes in Japanese hypertensive patients. *Hypertens Res* 2005;28:307-14. doi: 10.1291/hypres.28.307
71. Bond G, Dal Palu C, Hansson L, Magnani B, Mancia G, Neiss A, Rahn KH, Reid JL, Rodicio JL, Safar M, et al. The E.L.S.A. trial: protocol of a randomized trial to explore the differential effect of antihypertensive drugs on atherosclerosis in hypertension. *J Cardiovasc Pharmacol* 1994;23 Suppl 5:S85-7.
72. Zanchetti A, Bond MG, Hennig M, Neiss A, Mancia G, Dal Palu C, Hansson L, Magnani B, Rahn KH, Reid JL, et al. Calcium antagonist lacidipine slows down progression of asymptomatic carotid atherosclerosis: principal results of the European Lacidipine Study on Atherosclerosis (ELSA), a randomized, double-blind, long-term trial. *Circulation* 2002;106:2422-7. doi: 10.1161/01.cir.0000039288.86470.dd
73. Kasanuki H, Hagiwara N, Hosoda S, Sumiyoshi T, Honda T, Haze K, Nagashima M, Yamaguchi J, Origasa H, Urashima M, et al. Angiotensin II receptor blocker-based vs. non-angiotensin II receptor blocker-based therapy in patients with angiographically documented coronary artery disease and hypertension: the Heart Institute of Japan Candesartan Randomized Trial for Evaluation in Coronary Artery Disease (HIJ-CREATE). *Eur Heart J* 2009;30:1203-12. doi: 10.1093/eurheartj/ehp101
74. Brown MJ, Palmer CR, Castaigne A, de Leeuw PW, Mancia G, Rosenthal T, Ruilope LM. Morbidity and mortality in patients randomised to double-blind treatment with a long-acting calcium-channel blocker or diuretic in the International Nifedipine GITS study: Intervention as a Goal in Hypertension Treatment (INSIGHT). *Lancet* 2000;356:366-72. doi: 10.1016/S0140-6736(00)02527-7
75. Pepine CJ, Handberg EM, Cooper-DeHoff RM, Marks RG, Kowey P, Messerli FH, Mancia G, Cangiano JL, Garcia-Barreto D, Keltai M, et al. A calcium antagonist vs a non-calcium antagonist hypertension treatment strategy for patients with coronary artery disease. The International Verapamil-Trandolapril Study (INVEST): a randomized controlled trial. *JAMA* 2003;290:2805-16. doi: 10.1001/jama.290.21.2805

76. Yui Y, Sumiyoshi T, Kodama K, Hirayama A, Nonogi H, Kanmatsuse K, Origasa H, Iimura O, Ishii M, Saruta T, et al. Comparison of nifedipine retard with angiotensin converting enzyme inhibitors in Japanese hypertensive patients with coronary artery disease: the Japan Multicenter Investigation for Cardiovascular Diseases-B (JMIC-B) randomized trial. *Hypertens Res* 2004;27:181-91. doi: 10.1291/hypres.27.181
77. Dahlof B, Devereux RB, Julius S, Kjeldsen SE, Beevers G, de Faire U, Fyhrquist F, Hedner T, Ibsen H, Kristianson K, et al. Characteristics of 9194 patients with left ventricular hypertrophy: the LIFE study. Losartan Intervention For Endpoint Reduction in Hypertension. *Hypertension* 1998;32:989-97. doi: 10.1161/01.hyp.32.6.989
78. Dahlof B, Devereux RB, Kjeldsen SE, Julius S, Beevers G, de Faire U, Fyhrquist F, Ibsen H, Kristiansson K, Lederballe-Pedersen O, et al. Cardiovascular morbidity and mortality in the Losartan Intervention For Endpoint reduction in hypertension study (LIFE): a randomised trial against atenolol. *Lancet* 2002;359:995-1003. doi: 10.1016/S0140-6736(02)08089-3
79. Schrader J, Luders S, Kulschewski A, Hammersen F, Plate K, Berger J, Zidek W, Dominiak P, Diener HC, MOSES Study Group. Morbidity and Mortality After Stroke, Eprosartan Compared with Nitrendipine for Secondary Prevention: principal results of a prospective randomized controlled study (MOSES). *Stroke* 2005;36:1218-26. doi: 10.1161/01.STR.0000166048.35740.a9
80. National Intervention Cooperative Study in Elderly Hypertensives Study Group. Randomized double-blind comparison of a calcium antagonist and a diuretic in elderly hypertensives. . *Hypertension* 1999;34:1129-33.
81. The NORDIL Group. The Nordic Diltiazem Study (NORDIL). A prospective intervention trial of calcium antagonist therapy in hypertension. *Blood Press* 1993;2:312-21. doi: 10.3109/08037059309077174
82. ONTARGET Investigators, Yusuf S, Teo KK, Pogue J, Dyal L, Copland I, Schumacher H, Dagenais G, Sleight P, Anderson C. Telmisartan, ramipril, or both in patients at high risk for vascular events. *N Engl J Med* 2008;358:1547-59. doi: 10.1056/NEJMoa0801317
83. Hansson L, Lindholm LH, Ekbom T, Dahlof B, Lanke J, Schersten B, Wester PO, Hedner T, de Faire U. Randomised trial of old and new antihypertensive drugs in elderly patients: cardiovascular mortality and morbidity the Swedish Trial in Old Patients with Hypertension-2 study. *Lancet* 1999;354:1751-6. doi: 10.1016/s0140-6736(99)10327-1
84. Mann J, Julius S. The Valsartan Antihypertensive Long-term Use Evaluation (VALUE) trial of cardiovascular events in hypertension. Rationale and design. *Blood Press* 1998;7:176-83. doi: 10.1080/080370598437394
85. Julius S, Kjeldsen SE, Weber M, Brunner HR, Ekman S, Hansson L, Hua T, Laragh J, McInnes GT, Mitchell L, et al. Outcomes in hypertensive patients at high cardiovascular risk treated with regimens based on valsartan or amlodipine: the VALUE randomised trial. *Lancet* 2004;363:2022-31. doi: 10.1016/S0140-6736(04)16451-9
86. Rosei EA, Dal Palu C, Leonetti G, Magnani B, Pessina A, Zanchetti A, VHAS Investigators. Clinical results of the Verapamil in Hypertension and Atherosclerosis Study. *J Hypertens* 1997;15:1337-44. doi: 10.1097/00004872-199715110-00019
87. Major cardiovascular events in hypertensive patients randomized to doxazosin vs chlorthalidone: the antihypertensive and lipid-lowering treatment to prevent heart attack trial (ALLHAT). ALLHAT Collaborative Research Group. *JAMA* 2000;283:1967-75.
88. Ovbiagele B, Diener HC, Yusuf S, Martin RH, Cotton D, Vinisko R, Donnan GA, Bath PM, PRoFESS Investigators. Level of systolic blood pressure within the normal range and risk of recurrent stroke. *JAMA* 2011;306:2137-44. doi: 10.1001/jama.2011.1650
